# Causal link between mental disorders and gastrointestinal diseases: a Mendelian randomization study

**DOI:** 10.3389/fendo.2025.1288619

**Published:** 2025-04-22

**Authors:** Wenjing Ding, Liangliang Chen, Bei Pei, Dahong Gao, Jianguo Xia, Xuejun Li, Yougang Wang

**Affiliations:** ^1^ The Second Clinical Medical School, Anhui University of Chinese Medicine, Hefei, China; ^2^ Department of Gastroenterology, The Second Affiliated Hospital of Anhui University of Chinese Medicine, Hefei, China; ^3^ Department of Research, The Second Affiliated Hospital of Anhui University of Chinese Medicine, Hefei, China; ^4^ Department of Brain Disorders III, The Second Affiliated Hospital of Anhui University of Chinese Medicine, Hefei, China

**Keywords:** mental diseases, gastrointestinal diseases, Mendelian randomization, genome-wide association research, causality

## Abstract

**Background:**

Observational research suggests that mental diseases may increase the risk of gastrointestinal diseases. However, the causal link between these conditions remains unclear. In this study, we conducted a two-sample Mendelian randomization (MR) analysis to investigate the causal associations between common mental diseases and the risk of gastrointestinal diseases.

**Methods:**

First, a series of parameters were set to select single-nucleotide polymorphisms (SNPs) from genome-wide association studies (GWAS). Second, A two-sample Mendelian randomization analysis was conducted to investigate the causal link between mental diseases (Alzheimer’s disease, depression, major depressive disorder, Parkinson’s disease, schizophrenia) and gastrointestinal diseases (gastritis and duodenitis, gastric cancer) while removing outliers using MR-PRESSO. Finally, eight methods of MR analysis were used to generate forest plots, including inverse variance weighted (IVW), inverse variance weighted (fixed effects) (IVW fixed effects), maximum likelihood (ML), MR-Egger, weighted median, penalized weighted median, simple mode, and weighted mode, with IVW considered the primary method.

**Results:**

The result demonstrated that most MDs have no evidence of a causal link between gastrointestinal diseases except Parkinson’s disease and gastric cancer based on the IVW method (OR = 0.929 [95% CI = 0.869–0.992], *p* = 0.029). Subsequently, we performed a robustness analysis to ensure consistency.

**Conclusions:**

Our method provided evidence supporting a causal link between Parkinson’s disease and the risk of gastric cancer. However, no evidence was found for other mental diseases influencing the risk of gastrointestinal diseases. Further research is warranted to explore how mental diseases affect the development of gastrointestinal diseases.

## 1 Introduction

Gastrointestinal disease (GD) (1) is a condition affecting the esophagus, stomach, and duodenum. The most common forms of GDs include chronic atrophic gastritis (2), gastritis and duodenitis (3), gastric cancer (4) irritable bowel syndrome (5), and others. Gastritis and duodenitis (3) are chronic inflammatory diseases characterized by chronic, unexplained, moderate gastrointestinal symptoms. Gastric cancer is a globally significant disease and the third leading cause of cancer-related death (4). The common risk factors for gastric cancer include *Helicobacter pylori* infection, psychological stress, a high salt diet, and a lack of dietary fiber.

Mental diseases (MDs) are chronic psychiatric conditions characterized by behavioral and cognitive disorders worldwide. Individuals with MDs may experience severe physiological, psychological, and social consequences. In recent years, the potential link between MDs and GDs has garnered considerable attention from researchers (6, 7). A recent study reported that psychological factors, including anxiety and depression, are significantly associated with functional gastrointestinal disorders, particularly irritable bowel syndrome and functional dyspepsia (8). Based on a random community phone survey, the study demonstrated that individuals with irritable bowel syndrome have an increased risk of MDs (9). An epidemiological study confirmed a significant association between gastritis and the likelihood of MDs, with no gender differences observed (10). Several studies suggest two main mechanisms linking MDs and GDs. First, a bidirectional causal association between MDs and GDs is possible. For instance, patients with severe pain and/or functional limitations related to gastritis may experience increased anxiety or depression, and vice versa (11). Second, a shared genetic or environmental risk factor may exist for both GDs and MDs. A causal link between emotional or psychological stress and GDs has been recognized, particularly based on common genetic variants. On a biochemical level, neurotransmitters that influence the brain are also active in the gastrointestinal tract. For instance, serotonin, a neurotransmitter involved in many MDs, is also well-known to play a significant role in certain GDs (12). Although the causal link between MDs and GDs has not been definitively established, this issue can be addressed through Mendelian randomization (MR) analysis. Additionally, MDs and GDs have bidirectional relationships. Chronic gastrointestinal inflammation, including gastritis and duodenitis, disrupts gut microbiota balance and intestinal barrier integrity, triggering systemic inflammation and immune dysregulation (13). Proinflammatory cytokines, such as IL-6, TNF-α, and bacterial metabolites, including lipopolysaccharides, may cross the blood–brain barrier, promoting neuroinflammation and oxidative stress, which are considered key factors in neurodegeneration in both Alzheimer’s disease and Parkinson’s disease (14, 15).

MR analysis is a method that uses parameterized single-nucleotide polymorphisms (SNPs), known as genetic instrumental variants (IVs), to investigate the causal relationship between exposure data and outcome data, free from confounders. Previous research has revealed a causal link between depression and GDs (16). Chen et al. (17) analyzed the association between MDD and GDs. Other research has investigated the causal link between common MDs, including schizophrenia, depression, Alzheimer’s disease, Parkinson’s disease, epilepsy, and osteoporosis (18). However, it remains unclear whether, and to what extent, there is a causal link between MDs and GDs. Therefore, our study first explored the underlying relationship between MDs and the risk of GDs using a two-sample MR approach.

## 2 Materials and methods

### 2.1 Data available

**Figure 1:**
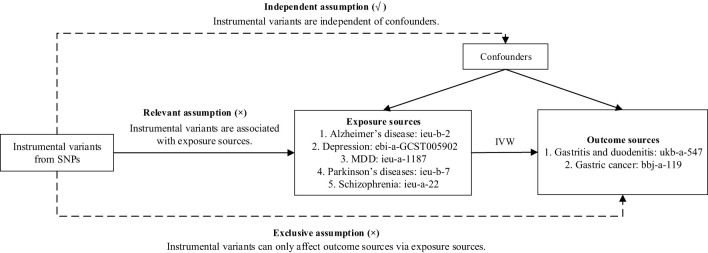
Flowchart of our Mendelian randomization analysis. IVW, inverse variance weighted; MDD, major depressive disorder; MR, Mendelian randomization; SNP, single-nucleotide polymorphism.

In this paper, we obtain two common GDs—(1) gastritis and duodenitis and (2) gastric cancer—as outcome datasets obtained from genome-wide association studies (GWAS) summary data (portal: https://gwas.mrcieu.ac.uk/). The selected outcome data information is as follows: For gastritis and duodenitis, the dataset ID is ukb-a-547, comprising 337,199 sample sizes (case = 8,080, control = 329,119) from the Neale Lab Consortium. For gastric cancer, the dataset ID is bbj-a-119, consisting of 202,308 sample sizes (case = 6,563, control = 195,745). We directly utilized these outcome datasets for further assessment. To establish the relationship between GDs and MDs, we selected MDs—including Alzheimer’s disease, depression, MDD, Parkinson’s disease, and schizophrenia—as exposure datasets. We chose from large public GWAS data, including Alzheimer’s disease (ieu-b-2) with 63,926 sample sizes (case = 21,982, control = 41,944) from Alzheimer Disease Genetics Consortium (ADGC), European Alzheimer’s Disease Initiative (EADI), Cohorts for Heart and Aging Research in Genomic Epidemiology Consortium (CHARGE), Genetic and Environmental Risk in AD/Defining Genetic, Polygenic and Environmental Risk for Alzheimer’s Disease Consortium (GERAD/PERADES), depression (ebi-a-GCST005902) with 322,580 sample sizes (case = 113,769, control = 208,811) from UK Biobank, MDD (ieu-a-1187) with 480,359 sample sizes (case = 135,458, control = 344,901) from Psychiatric Genomics Consortium, Parkinson’s diseases (ieu-b-7) with 482,730 sample sizes (case = 33,674, control = 449,056) from International Parkinson’s Disease Genomics Consortium. Schizophrenia (ieu-a-22) with 82,315 sample sizes (case = 135,458, control = 344,901) from Psychiatric Genomics Consortium. The flowchart of our MR study is depicted in **Figure 1**.

### 2.2 Genetic instrumental variants selection

We screened SNPs as genetic IVs based on Alzheimer’s disease, depression, MDD, Parkinson’s disease, and schizophrenia. SNPs were selected using a significance threshold of *p*-value < 5 × 10^−8^, and linkage disequilibrium (LD) was removed with an *r* ^2^ threshold of 0.001 and a distance threshold of 10,000 kb. The clumping selection was applied. Meanwhile, the *F*-statistic was calculated to quantify the intensity of genetic variation using the formula: $F=\frac{N-K-1}{K}\times \frac{1-R^{2}}{R^{2}}$ , where *N* is the sample size of exposure datasets, and *K* represents the number of SNPs. *R* ^2^ represents the genetic variant explanation of the exposure variance. *R* ^2^ can be calculated in two scenarios: When the value of effect allele frequency (EAF) exists, *R* ^2^ = 2 × EAF × (1 − EAF) × *β* ^2^. When the value of effect allele frequency (EAF) is not equal to NA, $R^{2}=\frac{2\;\times \text{ EAF }\times \;\left(1\;-\text{ EAF}\right)\;\times \;\beta^{2}}{2\;\times \text{ EAF }\times \;\left(1\;-\text{ EAF}\right)\times \;\beta^{2}+2\;\times {\text{SE}}^{2}\times \text{N}\times \text{ EAF }\times \;\left(1\;-\text{ EAF}\right)\;}$ , to avoid the EAF value participating in the calculation, where *β* is the effect of SNP on exposure, SE is the standard error of *β* (19, 20). In this paper, we define *F* > 10 as the genetic IVs’ strong correlation standards.

### 2.3 Two-sample MR statistical analysis

For MR analysis, three core assumptions regarding IVs (21, 22) must be addressed. First, the relevant assumption states that SNPs must be associated with exposure data (MDs). Second, the independent assumption requires that SNPs are not associated with the outcome data (GDs). Finally, the exclusion restriction assumption specifies that the outcome data (GDs) influence the exposure data (MDs) only through the selected SNPs. In particular, the MR-PRESSO package (23) was utilized to detect the horizontal pleiotropy by removing potential outliers. The significance threshold parameter of MR-PRESSO was set at 0.05, and the number of seeds was set to 1234. The detailed methods of MR analysis included inverse variance weighted (IVW), inverse variance weighted (fixed effects) (IVW fixed effects), maximum likelihood (ML), MR-Egger, weighted median, penalized weighted median, simple mode, and weighted mode, all of which were used to estimate the effects (24–26). Among these, IVW was the primary regression method (27). All analyses were conducted using R v.4.3.1 with the TwoSampleMR package (28), and a *p*-value < 0.05 was considered statistically significant.

## 3 Results

### 3.1 Information on instrumental variables

For the exposure dataset selection, we obtained 21 SNPs in Alzheimer’s disease, four SNPs in depression, 36 SNPs in MDD, 23 SNPs in Parkinson’s disease, and 83 SNPs in schizophrenia (selection conditions: *p* < 5 × 10^−8^, the threshold of r2 and kb are 0.001 and 10,000, respectively, *F* > 10). We then removed SNPs that were palindromic with intermediate allele frequencies. For gastritis and duodenitis, the removed SNPs were as follows: two SNPs (rs11257242, rs114812713) for Alzheimer’s disease, two SNPs (rs34215985, rs62099069) in MDD, one SNPs (rs10451230) in Parkinson’s disease, and nine SNPs (rs11139497, rs11191419, rs11740474, rs12325245, rs215411, rs2332700, rs281768, rs2851447, rs9607782) in schizophrenia. For gastric cancer, the removed SNPs are as follows: one SNPs (rs11257242) in Alzheimer’s disease, three SNPs (rs1363104, rs34215985, rs62099069) in MDD, two SNPs (rs10451230, rs823106) in Parkinson’s disease, and nine SNPs (rs11139497, rs11191419, rs11740474, rs12325245, rs215411, rs2332700, rs281768, rs2851447, rs9607782) in schizophrenia. The results of our MR analysis are provided in **Supplementary Materials 1, 2**.

### 3.2 Two-sample MR analysis for GWAS between MDs and gastrointestinal disease

#### 3.2.1 Causal link of Alzheimer’s disease with gastrointestinal disease

**Figure 2:**
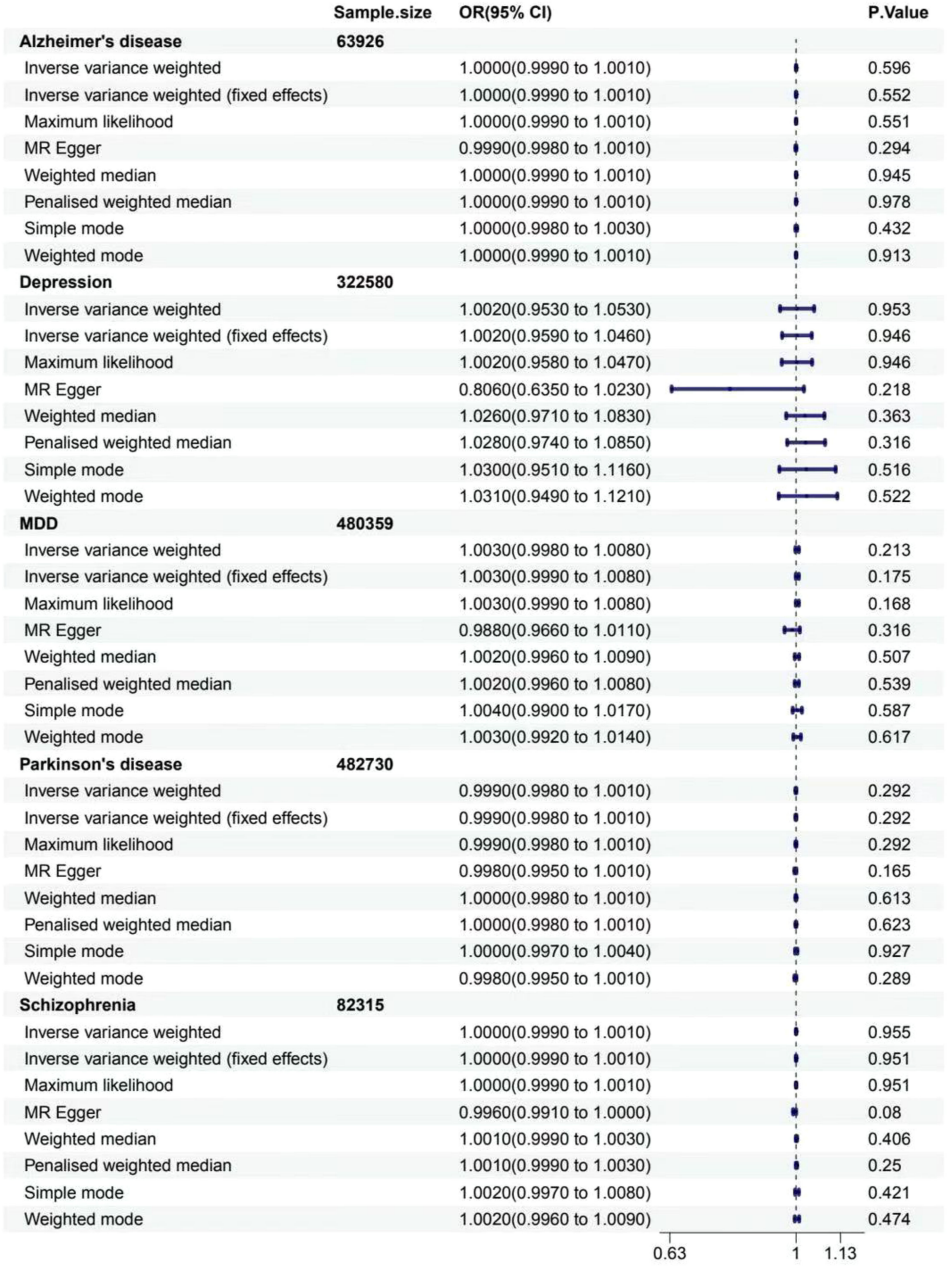
Associations between genetically predicted MDs and gastritis and duodenitis. The forest plot illustrates the causal relationship based on eight MR methods. OR, odds ratio; CI, confidence interval.

**Figure 3:**
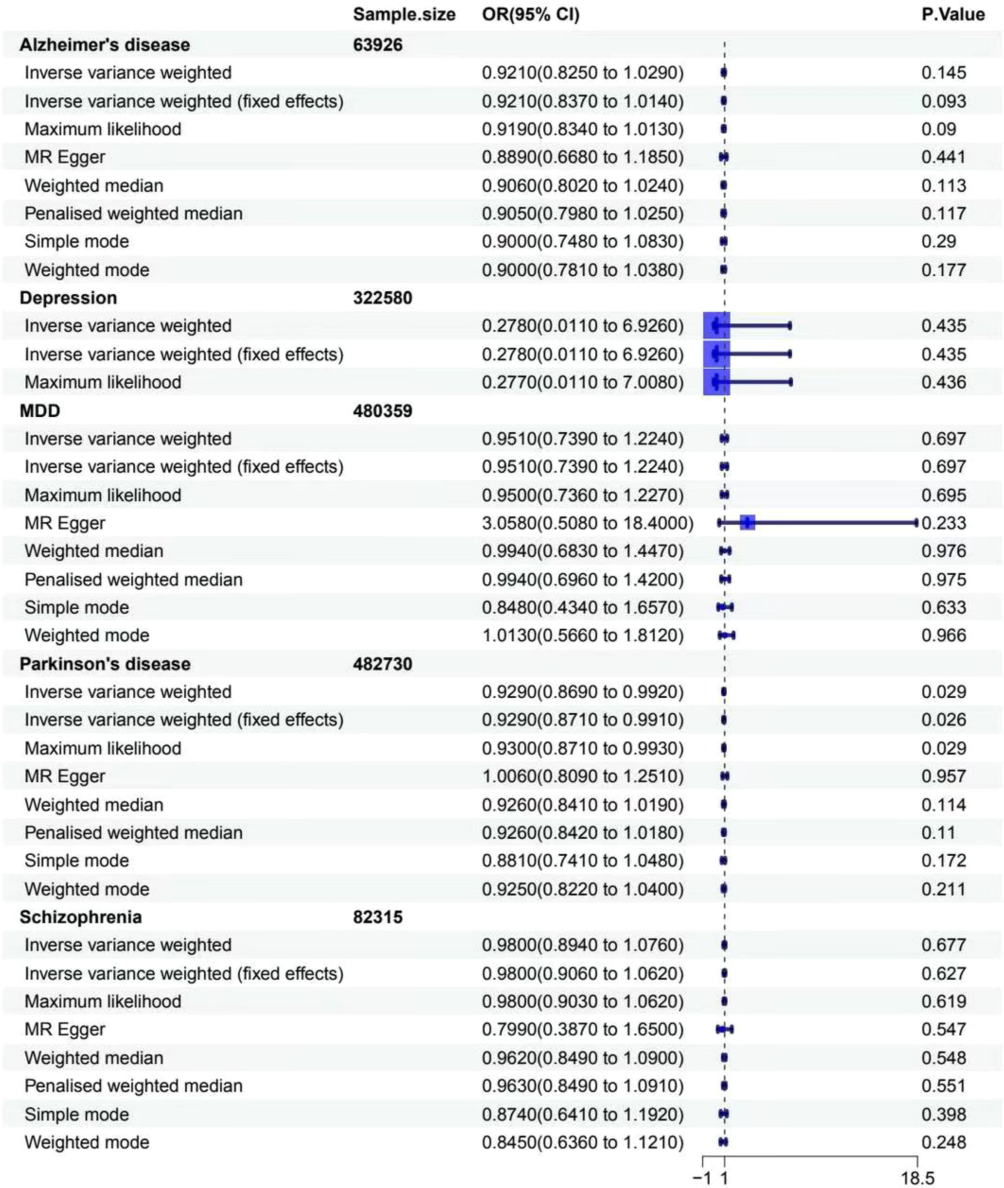
Associations between genetically predicted MDs and gastritis cancer. The forest plot illustrates the causal relationship based on eight MR methods. OR, odds ratio; CI, confidence interval.

For gastritis and duodenitis, Alzheimer’s disease showed no MR association, as shown in **Figure 2** (IVW: OR = 1.000 [95% CI = 0.999-1.001], *p* = 0.596; IVW (fixed effects): OR = 1.000 [95% CI = 0.999–1.001], *p* = 0.552; ML: OR = 1.000 [95% CI = 0.999–1.001], *p* = 0.551; MR-Egger: 0.999 [0.998–1.001], *p* = 0.294; weighted median: OR = 1.000 [95% CI = 0.999–1.001], *p* = 0.945; penalized weighted median: OR = 1.000 [95% CI = 0.999–1.001], *p* = 0.978; simple mode: OR = 1.000 [95% CI = 0.998–1.003], *p* = 0.432; weighted mode: OR = 1.000 [95% CI = 0.999–1.001], *p* = 0.913). For the gastric cancer, Alzheimer’s disease showed no MR association, as shown in **Figure 3** (IVW: OR = 0.921 [95% CI = 0.825–1.029], *p* = 0.145; IVW (fixed effects): OR = 0.921 [95% CI = 0.837–1.014], *p* = 0.093; ML: OR = 0.919 [95% CI = 0.834–1.013], *p* = 0.090; MR-Egger: 0.889 [0.668–1.185], *p* = 0.441; weighted median: OR = 0.906 [95% CI = 0.802–1.024], *p* = 0.113; penalized weighted median: OR = 0.905 [95% CI = 0.798–1.025], *p* = 0.117; simple mode: OR = 0.900 [95% CI = 0.748–1.083], *p* = 0.290; weighted mode: OR = 0.900 [95% CI = 0.781–1.038], *p* = 0.177).

#### 3.2.2 Causal link of depression with gastrointestinal disease

For gastritis and duodenitis, depression showed no MR association, as shown in **Figure 2** (IVW: OR = 1.002 [95% CI = 0.953–1.053], *p* = 0.953; IVW (fixed effects): OR = 1.002 [95% CI = 0.959–1.046], *p* = 0.946; ML: OR = 1.002 [95% CI = 0.958–1.047], *p* = 0.946; MR-Egger: 0.806 [0.635–1.023], *p* = 0.218; weighted median: OR = 1.026 [95% CI = 0.971–1.083], *p* = 0.363; penalized weighted median: OR = 1.028 [95% CI = 0.974–1.085], *p* = 0.316; simple mode: OR = 1.030 [95% CI = 0.951–1.116], *p* = 0.516; weighted mode: OR = 1.031 [95% CI = 0.949–1.121], *p* = 0.522). For gastric cancer, depression showed no MR association, as shown in **Figure 3** (IVW: OR = 0.278 [95% CI = 0.011–6.926], *p* = 0.435; IVW (fixed effects): OR = 0.278 [95% CI = 0.011–6.926], *p* = 0.435; ML: OR = 0.277 [95% CI = 0.011–7.008], *p* = 0.436).

#### 3.2.3 Causal link of MDD with gastrointestinal disease

For gastritis and duodenitis, MDD showed no MR association, as shown in **Figure 2** (IVW: OR = 1.003 [95% CI = 0.998–1.008], *p* = 0.213; IVW (fixed effects): OR = 1.003 [95% CI = 0.999–1.008], *p* = 0.175; ML: OR = 1.003 [95% CI = 0.999–1.008], *p* = 0.168; MR-Egger: 0.988 [0.966–1.011], *p* = 0.316; weighted median: OR = 1.002 [95% CI = 0.996–1.009], *p* = 0.507; penalized weighted median: OR = 1.002 [95% CI = 0.996–1.008], *p* = 0.539; simple mode: OR = 1.004 [95% CI = 0.990–1.017], *p* = 0.587; weighted mode: OR = 1.003 [95% CI = 0.992–1.014], *p* = 0.617). For gastric cancer, MDD showed no MR association, as shown in **Figure 3** (IVW: OR = 0.951 [95% CI = 0.739–1.224], *p* = 0.697; IVW (fixed effects): OR = 0.951 [95% CI = 0.739–1.224], *p* = 0.697; ML: OR = 0.950 [95% CI = 0.736–1.227], *p* = 0.695; MR-Egger: 3.058 [0.508–18.407], *p* = 0.233; weighted median: OR = 0.994 [95% CI = 0.683–1.447], *p* = 0.976; penalized weighted median: OR = 0.994 [95% CI = 0.696–1.420], *p* = 0.975; simple mode: OR = 0.848 [95% CI = 0.434–1.657], *p* = 0.633; weighted mode: OR = 1.013 [95% CI = 0.566–1.812], *p* = 0.966).

#### 3.2.4 Causal link of Parkinson’s disease with gastrointestinal disease

For gastritis and duodenitis, Parkinson’s disease showed no MR association, as shown in **Figure 2** (IVW: OR = 0.999 [95% CI = 0.998–1.001], *p* = 0.292; IVW (fixed effects): OR = 0.999 [95% CI = 0.998–1.001], *p* = 0.292; ML: OR = 0.999 [95% CI = 0.998–1.001], *p* = 0.292; MR-Egger: 0.998 [0.995–1.001], *p* = 0.165; weighted median: OR = 1.000 [95% CI = 0.998–1.001], *p* = 0.613; penalized weighted median: OR = 1.000 [95% CI = 0.998–1.001], *p* = 0.623; simple mode: OR = 1.000 [95% CI = 0.997–1.004], *p* = 0.927; weighted mode: OR = 0.998 [95% CI = 0.995–1.001], *p* = 0.289). For gastric cancer, interestingly, the results of IVW analysis method show that the causal link of Parkinson’s disease and the risk of gastric cancer is statistically significant in IVW method, as shown in **Figure 3** (*p* < 0.05) (IVW: OR = 0.929 [95% CI = 0.869–0.992], *p* = 0.029; IVW (fixed effects): OR = 0.929 [95% CI = 0.871–0.991], *p* = 0.026; ML: OR = 0.930 [95% CI = 0.871–0.993], *p* = 0.029; MR-Egger: 1.006 [0.809–1.251], *p* = 0.957; weighted median: OR = 0.926 [95% CI = 0.841–1.019], *p* = 0.114; penalized weighted median: OR = 0.926 [95% CI = 0.842–1.018], *p* = 0.110; simple mode: OR = 0.881 [95% CI = 0.741–1.048], *p* = 0.172; weighted mode: OR = 0.925 [95% CI = 0.822–1.040], *p* = 0.211).

#### 3.2.5 Causal link of schizophrenia with gastrointestinal disease

For gastritis and duodenitis, schizophrenia showed no MR association, as demonstrated in **Figure 2** (IVW: OR = 1.000 [95% CI = 0.999–1.001], *p* = 0.955; IVW (fixed effects): OR = 1.000 [95% CI = 0.999–1.001], *p* = 0.951; ML: OR = 1.000 [95% CI = 0.999–1.001], *p* = 0.951; MR-Egger: 0.996 [0.991–1.000], *p* = 0.080; weighted median: OR = 1.001 [95% CI = 0.999–1.003], *p* = 0.406; penalized weighted median: OR = 1.001 [95% CI = 0.999–1.003], *p* = 0.250; simple mode: OR = 1.002 [95% CI = 0.997–1.008], *p* = 0.421; weighted mode: OR = 1.002 [95% CI = 0.996–1.009], *p* = 0.474). For gastric cancer, schizophrenia showed no MR association, as demonstrated in **Figure 3** (IVW: OR = 0.980 [95% CI = 0.894–1.076], *p* = 0.677; IVW (fixed effects): OR = 0.980 [95% CI = 0.906–1.062], *p* = 0.627; ML: OR = 0.980 [95% CI = 0.903–1.062], *p* = 0.619; MR-Egger: 0.799 [0.387–1.650], *p* = 0.547; weighted median: OR = 0.962 [95% CI = 0.849–1.090], *p* = 0.548; penalized weighted median: OR = 0.963 [95% CI = 0.849–1.091], *p* = 0.551; simple mode: OR = 0.874 [95% CI = 0.641–1.192], *p* = 0.398; weighted mode: OR = 0.845 [95% CI = 0.636–1.121], *p* = 0.248).

#### 3.2.6 Robustness analysis

Cochran’s *Q* test was employed to analyze the heterogeneity based on IVW, as shown in **Table 1**. The results indicated no significant heterogeneity (29), with a *p*-value > 0.05, except for schizophrenia and the causality of gastric cancer (*p* = 0.03298014). Furthermore, the MR-Egger pleiotropy test showed no evidence of horizontal pleiotropy between MDs and GDs (*p-*value > 0.05) (30). Subsequent MR-PRESSO, leave-one-out analysis, and funnel plot analysis did not calculate any influential SNPs between MDs and the risk of GDs, as shown in **Supplementary Materials 3, 4**. For depression and gastric cancer, no intercept values or *p*-values were calculated, as the number of SNPs in depression is only 4, as depicted in **Table 1**, which is too small. The results of the MR-PRESSO global test (*p* > 0.05) are shown in **Supplementary Material 5**.

**Table 1:** IVW heterogeneity test and MR-Egger pleiotropy test between mental diseases and gastrointestinal diseases.

Exposure	Outcome	IVW heterogeneity test		MR-Egger pleiotropy test	
		*Q*	*p*-Value	Intercept	*p*-Value
Alzheimer’s disease	Gastritis and duodenitis	20.125	0.215	0.0002	0.323
	Gastric cancer	14.640	0.200	0.005	0.799
Depression	Gastritis and duodenitis	3.848	0.278	0.002	0.211
	Gastric cancer	0.220	0.639	NA	NA
MDD	Gastritis and duodenitis	34.341	0.227	0.0005	0.197
	Gastric cancer	23.635	0.651	-0.037	0.209
Parkinson’s disease	Gastritis and duodenitis	20.541	0.487	0.000	0.278
	Gastric cancer	16.698	0.405	-0.013	0.461
Schizophrenia	Gastritis and duodenitis	82.001	0.118	0.0003	0.067
	Gastric cancer	80.489	0.033	0.015	0.580

IVW, inverse variance weighted; MR, Mendelian randomization; MDD, major depressive disorder.

## 4 Discussion

It is particularly notorious that our study is the first to investigate the association between five MDs and the risk of two GDs. Using the largest publicly available dataset, we conducted a two-sample MR analysis to assess the causal link between five MDs (Alzheimer’s disease, depression, MDD, Parkinson’s disease, schizophrenia) as exposure data and two GDs (gastritis and duodenitis, gastric cancer). Our findings demonstrated no significant association between MDs and GDs, except for Parkinson’s disease and gastric cancer (IVW: OR = 0.929 [95% CI = 0.869–0.992], *p* = 0.029).

In recent years, various studies have explored the causal relationship between MDs and GDs. Epidemiological research suggests a significant link between Alzheimer’s disease/Parkinson’s disease and gastric cancer. Some findings indicate that the risk of certain carcinogenic processes may be reduced after developing Alzheimer’s disease/Parkinson’s disease (31). A recent study using an Alzheimer’s disease mouse model demonstrated improvements in short-term memory and cognitive function, which were associated with specific gut microbiota, including *Proteobacteria*, *Verrucomicrobia*, and *Akkermansia*, and their potential link to GDs (32). Meanwhile, some studies suggest that diverse diets can alter the gut microbiome, which in turn may influence the incidence of Parkinson’s disease through its impact on GDs (33). Compared with healthy individuals, patients diagnosed with Parkinson’s disease exhibit significant differences in gut microbiome composition, including an increase in specific microbial populations (34). A meta-analysis conducted by Fu et al. (35) explored relationships between Alzheimer’s disease/Parkinson’s disease and intestinal disorders. These studies indicate causal relationships between Alzheimer’s disease/Parkinson’s disease and gut microbiome, which are closely associated with CDs (36, 37). Although Parkinson’s disease and gastric cancer have distinct primary pathologies, they may share overlapping mechanisms or risk factors. Emerging evidence suggests potential connections through genetic, molecular, and environmental pathways. Notably, the *LRRK2* gene, which is linked to familial Parkinson’s disease, has also been implicated in cancer pathways (38). Parkinson’s disease is increasingly associated with gastrointestinal dysfunction and altered gut microbiota, which often precede motor symptoms (39). Chronic gut inflammation or dysbiosis—particularly involving *H. pylori*, a well-known risk factor for gastric cancer—might create a proinflammatory environment that exacerbates Parkinson’s disease pathology while promoting gastric carcinogenesis (40).

A recent study suggested that patients diagnosed with *H. pylori* infection-related gastritis are at significantly increased risk of experiencing mental distress, which may lead to depression (41). Another study revealed that anxiety and depression are more prevalent in patients with gastritis who also suffer from postprandial dyspepsia (42). Doctors are advised to be conscious of the possibility of neuropsychiatric symptoms, including depression and anxiety when treating gastritis (43). A prospective study discovered the prevalence of *H. pylori* and depression in patients with GDs and assessed the outcome after certain mental interventions. This result indicated that *H. pylori* eradication therapy of GDs and mental interventions are beneficial (44).

For MDD, a case-controlled investigation involving 36 subjects found that MDD was significantly more common in patients with acute duodenitis compared to their respective controls (45). Meanwhile, individuals with gastritis often experience malabsorption of nutrients, such as iron and B vitamins (including folic acid), which are essential for maintaining brain function. A deficiency in these nutrients is associated with the development of MDD (46). Additionally, socioeconomic factors may influence dietary habits and mental status. Long-term exposure to chronic stress may contribute to the progression of both chronic gastritis and MDD (47).

Autoimmune diseases can be triggered by dietary ingredients and antigens from the gastrointestinal tract, with both genetic and environmental factors interacting. Additionally, the causal relationship between autoimmune diseases and schizophrenia has been explored for some time (48). Previous studies have used biomarkers of physiological processes and behavioral indices to better understand the effects of the gut microbiome on the brain in individuals with schizophrenia, which could be used as inclusion criteria in clinical trials (49–51).

Genetic factors play a variable, but generally modest, role in the development of gastritis, duodenitis, and gastric cancer. For gastritis and duodenitis, hereditary influences account for approximately 5%–10% of cases (52), primarily through polymorphisms in immune-related genes, such as IL-1β and TNF-α, which modulate inflammatory responses to triggers like *H. pylori* infection (53). In contrast, gastric cancer has a stronger genetic component, with approximately 10%–15% of cases linked to inherited predisposition (54). Nonetheless, environmental factors, including *H. pylori* infection, daily diet, and smoking, remain dominant drivers across all three aforementioned GDs (55), and the utility of our MR analysis may be limited. Therefore, further research is needed to explore additional links between MDs and GDs.

Our MR analysis has several advantages. First, this is the first MR analysis to explore the causal link between MDs and GDs. Furthermore, the publicly available GWAS datasets were of high quality and reliability. Finally, the time spent on our MR research was reasonable. Nevertheless, our research also had some limitations. A major shortcoming is that the types of GDs are limited. We only utilized two diseases (gastritis and duodenitis, gastric cancer) for the two-sample MR analysis, and the results may not be extensive. More GDs should be selected to explore the causal link in future studies. Second, we did not employ a reverse MR analysis (56, 57) to further explore the relationship between MDs and GDs. A bidirectional MR should be used to investigate the causal relationships between MDs and GDs. Third, our two-sample MR analysis did not address the issue of sample overlap between the exposure (MDs) and outcome (GDs) datasets, which could affect the accuracy of the results with high overlap. Fourth, our study relied on a single GWAS database for each disease, which may lead to unreliable results. Further analyses, including the use of multiple databases and conducting a meta-analysis, should be performed.

## 5 Conclusion

In conclusion, our two-sample MR study revealed underlying causal associations between Parkinson’s disease and the risk of gastric cancer. Further research is essential to explore whether MDs contribute to the risk of GDs. Additional studies are needed to confirm these causal links and examine the potential mechanisms between MDs and GDs.

## Data Availability

The original contributions presented in the study are included in the article/**Supplementary Material**. Further inquiries can be directed to the corresponding author.

## References

[1] Wang WW Gu WL He C Zhang T Shen Y Pu YQ. Bioactive components of Banxia Xiexin Decoction for the treatment of gastrointestinal diseases based on flavor-oriented analysis. J Ethnopharmacol. (2022) 291:115085. doi: 10.1016/j.jep.2022.115085 35150814

[2] Holleczek B Schottker B Brenner H. Helicobacter pylori infection, chronic atrophic gastritis and risk of stomach and esophagus cancer: results from the prospective population-based ESTHER cohort study. Int J Cancer. (2020) 146:2773–83. doi: 10.1002/ijc.32610 31376284

[3] Chehade M Tan JW Gehman LT. Gastroenterology practice patterns contribute to missed diagnoses of eosinophilic gastritis and duodenitis. Gastro Hep Adv. (2023) 3:334–42. doi: 10.1016/j.gastha.2022.11.010 PMC11308756 39132645

[4] Smyth EC Nilsson M Grabsch HI Grieken NC Lordick F. Gastric cancer. Lancet. (2020) 396:635–48. doi: 10.1016/S0140-6736(20)31288-5 32861308

[5] Oka P Parr H Barberio B Black CJ Savarino EV Ford AC. Global prevalence of irritable bowel syndrome according to Rome III or IV criteria: a systematic review and meta-analysis. Lancet Gastroenterol Hepatol. (2020) 5:908–17. doi: 10.1016/S2468-1253(20)30217-X 32702295

[6] Goodwin RD Cox BJ Clara I. Neuroticism and physical disorders among adults in the community: results from the National Comorbidity Survey. J Behav Med. (2006) 29:229–38. doi: 10.1007/s10865-006-9048-5 16724279

[7] Walker JR Ediger JP Graff LA Greenfeld JM Clara I Lix L. The Manitoba IBD cohort study: a population-based study of the prevalence of lifetime and 12-month anxiety and mood disorders. Am J Gastroenterol. (2008) 103:1989–97. doi: 10.1111/j.1572-0241.2008.01980.x 18796096

[8] Koloski N Holtmann G Talley NJ. Is there a causal link between psychological disorders and functional gastrointestinal disorders? Expert Rev Gastroenterol Hepatol. (2020) 14:1047–59. doi: 10.1080/17474124.2020.1801414 32715790

[9] Lee S Wu J Ma YL Tsang A Guo WJ Sung J. Irritable bowel syndrome is strongly associated with generalized anxiety disorder: a community study. Aliment Pharmacol Ther. (2009) 30:643–51. doi: 10.1080/17474124.2020.1801414 19552631

[10] Goodwin RD Cowles RA Galea S Jacobi F. Gastritis and mental disorders. J Psychiatr Res. (2013) 47:128–32. doi: 10.1016/j.jpsychires.2012.09.016 23073472

[11] Konturek PC Brzozowski T Konturek SJ. Stress and the gut: pathophysiology, clinical consequences, diagnostic approach, and treatment options. J Physiol Pharmacol. (2011) 62:591–9. 22314561

[12] Sikander A Rana SV Prasad KK. Role of serotonin in gastrointestinal motility and irritable bowel syndrome. Clin Chim Acta. (2009) 403:47–55. doi: 10.1016/j.cca.2009.01.028 19361459

[13] Rudzki L Szulc A. Immune gate” of psychopathology-The role of gut derived immune activation in major psychiatric disorders. Front Psychiatry. (2018) 29:205. doi: 10.3389/fpsyt.2018.00205 PMC5987016 29896124

[14] Kearns R. Gut-Brain Axis and Neuroinflammation: The role of gut permeability and the Kynurenine pathway in neurological disorders. Cell Mol Neurobiol. (2024) 44:64. doi: 10.1007/s10571-024-01496-z 39377830 PMC11461658

[15] Monda A La Torre ME Messina A Di Maio G Monda V Moscatelli F. Exploring the ketogenic diet’s potential in reducing neuroinflammation and modulating immune responses. Front Immunol. (2024) 15:1425816. doi: 10.3389/fimmu.2024.1425816 39188713 PMC11345202

[16] Ruan XX Chen J Sun YH Zhang Y Zhao JH Wang XY. Depression and 24 gastrointestinal diseases: a Mendelian randomization study. Transl Psychiatry. (2023) 13:146. doi: 10.1038/s41398-023-02459-6 37142593 PMC10160129

[17] Chen DZ Zhang YL Huang T Jia JZ. Depression and risk of gastrointestinal disorders: a comprehensive two-sample Mendelian randomization study of European ancestry. Psychol Med. (2023) 53:7309–21. doi: 10.1017/S0033291723000867 37183395

[18] Tang F Wang S Zhao HX Xia DM Dong X. Mendelian randomization analysis does not reveal a causal influence of mental diseases on osteoporosis. Front Endocrinol. (2023) 14:1125427. doi: 10.3389/fendo.2023.1125427 PMC10157183 37152964

[19] Gill D Efstathiadou A Cawood K Tzoulaki I Dehghan A. Education protects against coronary heart disease and stroke independently of cognitive function: evidence from Mendelian randomization. Int J Epidemiol. (2019) 48:1468–77. doi: 10.1093/ije/dyz200 PMC6857750 31562522

[20] Levin MG Judy R Gill D Vujkovic M Verma SS Bradford Y. Genetics of height and risk of atrial fibrillation: A Mendelian randomization study. PloS Med. (2020) 17:e1003288. doi: 10.1371/journal.pmed.1003288 33031386 PMC7544133

[21] Haycock PC Burgess S Wade KH Bowden J Relton C Davey Smith G. Best (but oft-forgotten) practices: the design, analysis, and interpretation of Mendelian randomization studies. Am J Clin Nutr. (2016) 103:965–78. doi: 10.3945/ajcn.115.118216 PMC4807699 26961927

[22] Freuer D Meisinger C. Causal link between thyroid function and schizophrenia: a two-sample Mendelian randomization study. Eur J Epidemiol. (2023) 38:1081–8. doi: 10.1007/s10654-023-01034-z PMC10570193 37589836

[23] Schmidt AF Finan C Gordillo-Maranon M Asselbergs FW Freitag DF Patel RS. Genetic drug target validation using Mendelian randomisation. Nat Commun. (2020) 11:3255. doi: 10.1038/s41467-020-16969-0 32591531 PMC7320010

[24] Burgess S Butterworth A Thompson SG. Mendelian randomization analysis with multiple genetic variants using summarized data. Genet Epidemiol. (2013) 37:658–65. doi: 10.1002/gepi.21758 PMC4377079 24114802

[25] Hartwig FP Davey SG Bowden J. Robust inference in summary data Mendelian randomization via the zero modal pleiotropy assumption. Int J Epidemiol. (2017) 46:1985–98. doi: 10.1093/ije/dyx102 PMC5837715 29040600

[26] Bowden J Davey SG Burgess S. Mendelian randomization with invalid instruments: effect estimation and bias detection through Egger regression. Int J Epidemiol. (2015) 44:512–25. doi: 10.1093/ije/dyv080 PMC4469799 26050253

[27] Bowden J Davey SG Haycock PC Burgess S. Consistent estimation in Mendelian randomization with some invalid instruments using a weighted median estimator. Genet Epidemiol. (2016) 40:304–14. doi: 10.1002/gepi.21965 PMC4849733 27061298

[28] Hemani G Zheng J Elsworth B Wade KH Haberland V Baird D. The MR-Base platform supports systematic causal inference across the human phenome. Elife. (2018) 7:e34408. doi: 10.7554/eLife.34408 29846171 PMC5976434

[29] Papadimitriou N Dimou N Tsilidis KK Banbury B Martin RM Lewis S. Physical activity and risks of breast and colorectal cancer: a Mendelian randomisation analysis. Nat Commun. (2020) 11:597. doi: 10.1038/s41467-020-14389-8 32001714 PMC6992637

[30] Long YW Tang LH Zhou YY Zhao SS Zhu H. Causal relationship between gut microbiota and cancers: a two-sample Mendelian randomisation study. BMC Med. (2023) 21:66. doi: 10.1186/s12916-023-02761-6 36810112 PMC9945666

[31] Hang ZC Lei T Zeng ZH Cai SL Bi WY Du HW. Composition of intestinal flora affects the risk relationship between Alzheimer’s disease/Parkinson’s disease and cancer. BioMed Pharmacother. (2022) 145:112343. doi: 10.1016/j.biopha.2021.112343 34864312

[32] Sun J Xu JX Ling Y Wang FY Gong TY Yang CW. Fecal microbiota transplantation alleviated Alzheimer’s disease-like pathogenesis in APP/PS1 transgenic mice. Transl Psychiatry. (2019) 9:189. doi: 10.1038/s41398-019-0525-3 31383855 PMC6683152

[33] Heiman ML Greenway FL. A healthy gastrointestinal microbiome is dependent on dietary diversity. Mol Metab. (2016) 5:317–20. doi: 10.1016/j.molmet.2016.02.005 PMC4837298 27110483

[34] Jackson A Forsyth CB Shaikh M Voigt RM Engen PA Ramirez V. Diet in Parkinson’s Disease: critical role for the microbiome. Front Neurol. (2019) 10:1245. doi: 10.3389/fneur.2019.01245 31920905 PMC6915094

[35] Fu PF Gao M Yung KKL. Association of intestinal disorders with Parkinson’s disease and Alzheimer’s disease: A systematic review and meta-analysis. ACS Chem Neurosci. (2020) 11:395–405. doi: 10.1021/acschemneuro.9b00607 31876406

[36] Gorkiewicz G Moschen A. Gut microbiome: a new player in gastrointestinal disease. Virchows Arch. (2018) 472:159–72. doi: 10.1007/s00428-017-2277-x PMC5849673 29243124

[37] Trakman GL Fehily S Basnayake C Hamilton AL Russell E Wilson-O'Brien A. Diet and gut microbiome in gastrointestinal disease. J Gastroenterol Hepatol. (2022) 37:237–45. doi: 10.1111/jgh.15728 34716949

[38] Zhang XY Guarin D Mohammadzadehhonarvar N Chen XQ Gao X. Parkinson’s disease and cancer: a systematic review and meta-analysis of over 17 million participants. BMJ Open. (2021) 11:e046329. doi: 10.1136/bmjopen-2020-046329 PMC8256737 34215604

[39] Metta V Leta V Mrudula KR Prashanth LK Goyal V Borgohain R. Gastrointestinal dysfunction in Parkinson’s disease: molecular pathology and implications of gut microbiome, probiotics, and fecal microbiota transplantation. J Neurol. (2022) 269:1154–63. doi: 10.1007/s00415-021-10567-w 33881598

[40] Alfonsetti M Castelli V d’Angelo M. Are we what we eat? Impact of diet on the gut-brain axis in Parkinson’s disease. Nutrients. (2022) 14:380. doi: 10.3390/nu14020380 35057561 PMC8780419

[41] Takeoka A Tayama J Kobayashi M Sagara I Ogawa S Saigo T. Psychological effects of Helicobacter pylori-associated atrophic gastritis in patients under 50 years: A cross-sectional study. Helicobacter. (2017) 22:e12445. doi: 10.1111/hel.12445 29034535

[42] Piriyapong K Tangaroonsanti A Mahachai V Vilaichone RK. Helicobacter pylori infection impacts on functional dyspepsia in Thailand. Asian Pac J Cancer Prevent. (2014) 15:10887–91. doi: 10.7314/apjcp.2014.15.24.10887 25605196

[43] Neufeld N Mohamed N Grujich N Shulman K. Acute neuropsychiatric symptoms associated with antibiotic treatment of helicobacter pylori infections: a review. J Psychiatr Prac. (2017) 23:25–35. doi: 10.1097/PRA.0000000000000205 28072642

[44] Kabeer KK Ananthakrishnan N Anand C Balasundaram S. Prevalence of Helicobacter pylori infection and stress, anxiety or depression in functional dyspepsia and outcome after appropriate intervention. J Clin Diagn Res. (2017) 11:VC11–5. doi: 10.7860/JCDR/2017/26745.10486 PMC5620891 28969250

[45] Magni G Salmi A Paterlini A Merlo A. Psychological distress in duodenal ulcer and acute gastroduodenitis. A Controlled study Dig Dis Sci. (1982) 27:1081–4. doi: 10.1007/BF01391444 7172957

[46] Franceschi F Annalisa T Teresa DR Giovanna D Ianiro G Franco S. Role of Helicobacter pylori infection on nutrition and metabolism. World J Gastroenterol. (2014) 20:12809–17. doi: 10.3748/wjg.v20.i36.12809 PMC4177464 25278679

[47] Ochi M Fujiwara T Mizuki R Kawakami N. Association of socioeconomic status in childhood with major depression and generalized anxiety disorder: results from the World Mental Health Japan survey 2002-2006. BMC Public Health. (2014) 14:359. doi: 10.1186/1471-2458-14-359 24735450 PMC3991871

[48] Severance EG Yolken RH Eaton WW. Autoimmune diseases, gastrointestinal disorders and the microbiome in schizophrenia: more than a gut feeling. Schizophr Res. (2016) 176:23–35. doi: 10.1016/j.schres.2014.06.027 25034760 PMC4294997

[49] Elder JH. The gluten-free, casein-free diet in autism: an overview with clinical implications. Nutr Clin Pract. (2008) 23:583–8. doi: 10.1177/0884533608326061 19033217

[50] Marcason W. What is the current status of research concerning use of a gluten-free, casein-free diet for children diagnosed with autism? J Am Diet Assoc. (2009) 109:572. doi: 10.1016/j.jada.2009.01.013 19248872

[51] Whiteley P Haracopos D Knivsberg AM Reichelt KL Parlar S Jacobsen J. The ScanBrit randomised, controlled, single-blind study of a gluten- and casein-free dietary intervention for children with autism spectrum disorders. Nutr Neurosci. (2010) 13:87–100. doi: 10.1179/147683010X12611460763922 20406576

[52] Balakrishnan M George R Sharma A Graham DY. Changing trends in stomach cancer throughout the world. Curr Gastroenterol Rep. (2017) 19:36. doi: 10.1007/s11894-017-0575-8 28730504 PMC6918953

[53] Bergamo A Gerdol M Pallavicini A Greco S Schepens I Hamelin R. Lysozyme-induced transcriptional regulation of TNF-α pathway genes in cells of the monocyte lineage. Int J Mol Sci. (2019) 20:5502. doi: 10.3390/ijms20215502 31694163 PMC6862675

[54] Mocellin S Verdi D Pooley KA Nitti D. Genetic variation and gastric cancer risk: a field synopsis and meta-analysis. Gut. (2015) 64:1209–19. doi: 10.1136/gutjnl-2015-309168 25731870

[55] Richard ML Sokol H. The gut mycobiota: insights into analysis, environmental interactions and role in gastrointestinal diseases. Nat Rev Gastroenterol Hepatol. (2019) 16:331–45. doi: 10.1038/s41575-019-0121-2 30824884

[56] Burgess S Swanson SA Labrecque JA. Are Mendelian randomization investigations immune from bias due to reverse causation? Eur J Epidemiol. (2021) 36:253–7. doi: 10.1007/s10654-021-00726-8 PMC8032609 33611685

[57] Lutz SM Wu AC Hokanson JE Vansteelandt S Lange C. Caution against examining the role of reverse causality in Mendelian Randomization. Genet Epidemiol. (2021) 45:445–54. doi: 10.1002/gepi.22385 PMC8222166 34008876

