# Supplementary material for: Causal link between mental disorders and gastrointestinal diseases: a Mendelian randomization study
Source: Front Endocrinol (Lausanne). 2025 Apr 22;16:1288619. doi: 10.3389/fendo.2025.1288619 (PMC12052545; doi:10.3389/fendo.2025.1288619)

	Sample.size	OR(95% CI)	P.Value
**Alzheimer's disease**	**63926**		
Inverse variance weighted		1.0000(0.9990 to 1.0010)	0.596
Inverse variance weighted (fixed effects)		1.0000(0.9990 to 1.0010)	0.552
Maximum likelihood		1.0000(0.9990 to 1.0010)	0.551
MR Egger		0.9990(0.9980 to 1.0010)	0.294
Weighted median		1.0000(0.9990 to 1.0010)	0.945
Penalised weighted median		1.0000(0.9990 to 1.0010)	0.978
Simple mode		1.0000(0.9980 to 1.0030)	0.432
Weighted mode		1.0000(0.9990 to 1.0010)	0.913
**Depression**	**322580**		
Inverse variance weighted		1.0020(0.9530 to 1.0530)	0.953
Inverse variance weighted (fixed effects)		1.0020(0.9590 to 1.0460)	0.946
Maximum likelihood		1.0020(0.9580 to 1.0470)	0.946
MR Egger		0.8060(0.6350 to 1.0230)	0.218
Weighted median		1.0260(0.9710 to 1.0830)	0.363
Penalised weighted median		1.0280(0.9740 to 1.0850)	0.316
Simple mode		1.0300(0.9510 to 1.1160)	0.516
Weighted mode		1.0310(0.9490 to 1.1210)	0.522
**MDD**	**480359**		
Inverse variance weighted		1.0030(0.9980 to 1.0080)	0.213
Inverse variance weighted (fixed effects)		1.0030(0.9990 to 1.0080)	0.175
Maximum likelihood		1.0030(0.9990 to 1.0080)	0.168
MR Egger		0.9880(0.9660 to 1.0110)	0.316
Weighted median		1.0020(0.9960 to 1.0090)	0.507
Penalised weighted median		1.0020(0.9960 to 1.0080)	0.539
Simple mode		1.0040(0.9900 to 1.0170)	0.587
Weighted mode		1.0030(0.9920 to 1.0140)	0.617
**Parkinson's disease**	**482730**		
Inverse variance weighted		0.9990(0.9980 to 1.0010)	0.292
Inverse variance weighted (fixed effects)		0.9990(0.9980 to 1.0010)	0.292
Maximum likelihood		0.9990(0.9980 to 1.0010)	0.292
MR Egger		0.9980(0.9950 to 1.0010)	0.165
Weighted median		1.0000(0.9980 to 1.0010)	0.613
Penalised weighted median		1.0000(0.9980 to 1.0010)	0.623
Simple mode		1.0000(0.9970 to 1.0040)	0.927
Weighted mode		0.9980(0.9950 to 1.0010)	0.289
**Schizophrenia**	**82315**		
Inverse variance weighted		1.0000(0.9990 to 1.0010)	0.955
Inverse variance weighted (fixed effects)		1.0000(0.9990 to 1.0010)	0.951
Maximum likelihood		1.0000(0.9990 to 1.0010)	0.951
MR Egger		0.9960(0.9910 to 1.0000)	0.08
Weighted median		1.0010(0.9990 to 1.0030)	0.406
Penalised weighted median		1.0010(0.9990 to 1.0030)	0.25
Simple mode		1.0020(0.9970 to 1.0080)	0.421
Weighted mode		1.0020(0.9960 to 1.0090)	0.474

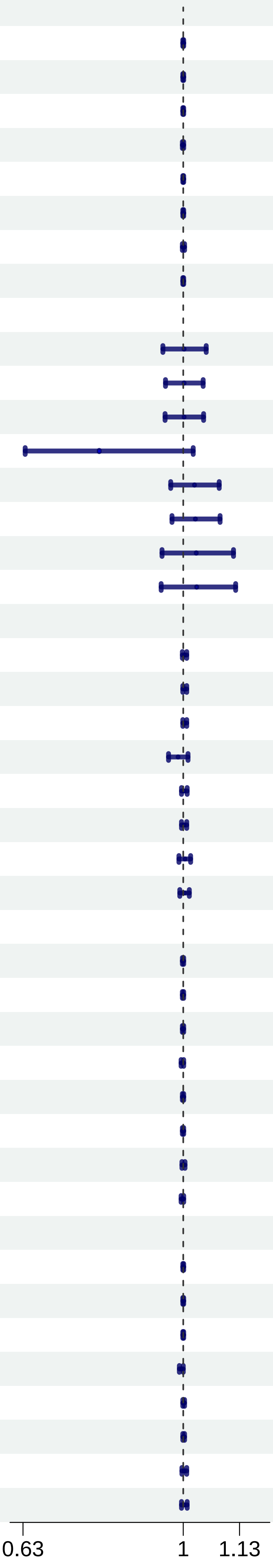

Supplement: Supplementary file 1 [file DataSheet1.pdf]
