# Supplementary figures and images for: Causal link between mental disorders and gastrointestinal diseases: a Mendelian randomization study
Source: Front Endocrinol (Lausanne). 2025 Apr 22;16:1288619. doi: 10.3389/fendo.2025.1288619 (PMC12052545; doi:10.3389/fendo.2025.1288619)

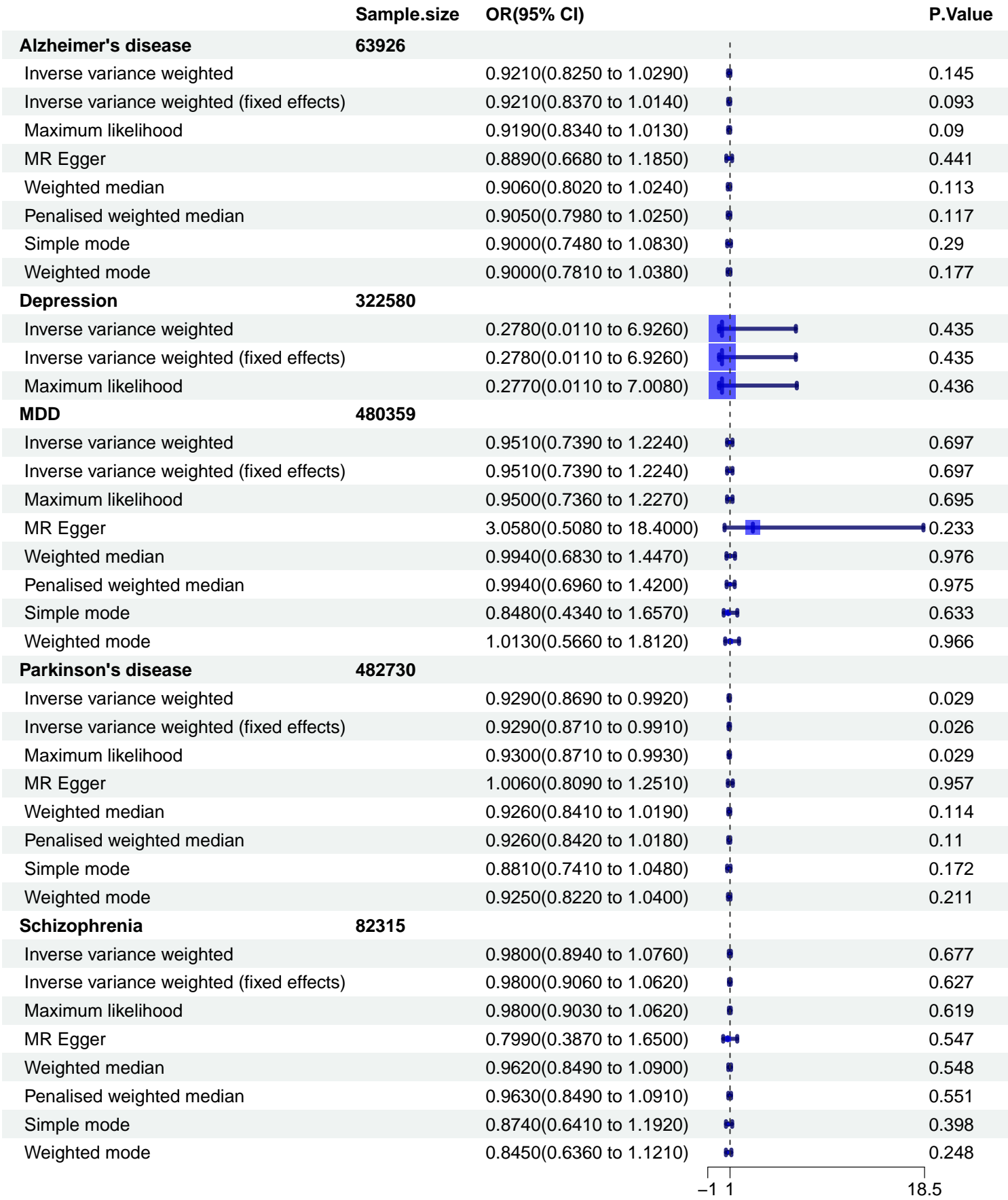

Supplement: Supplementary file 2 [file DataSheet2.pdf]

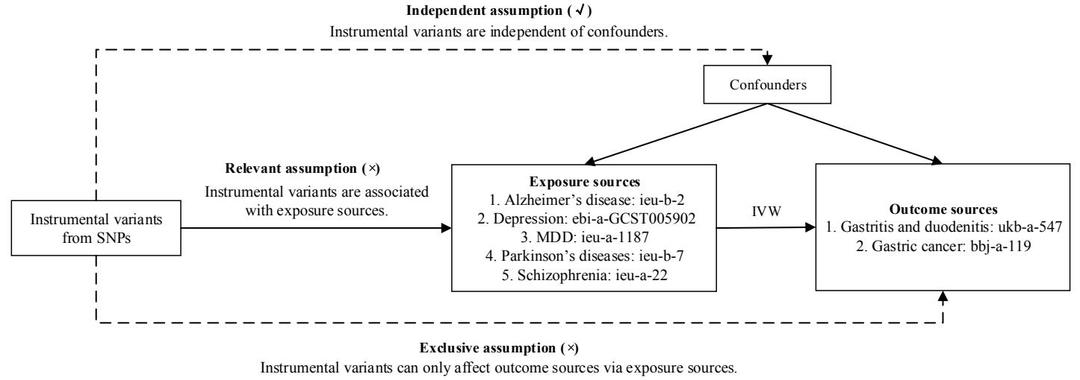

Supplement: Supplementary file 8 [file Image1.jpeg]
