# Supplementary material for: Causal link between mental disorders and gastrointestinal diseases: a Mendelian randomization study
Source: Front Endocrinol (Lausanne). 2025 Apr 22;16:1288619. doi: 10.3389/fendo.2025.1288619 (PMC12052545; doi:10.3389/fendo.2025.1288619)
Supplement: Supplementary file 3 [file DataSheet3.pdf]

Figure S1 Forest plot, scatter plot, leave-one-out analysis and funnel plot for MDs on gastritis and duodenitis.

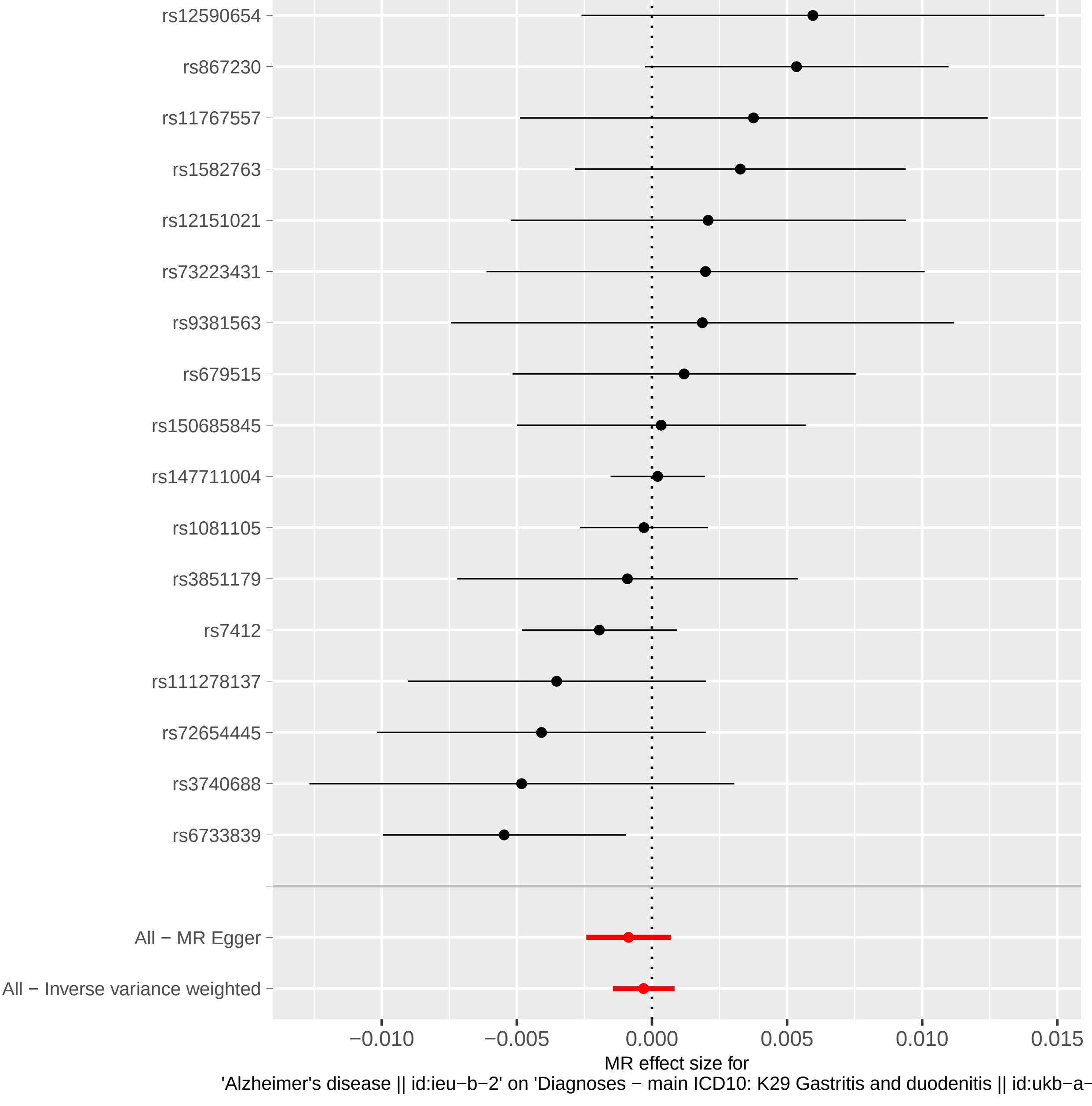

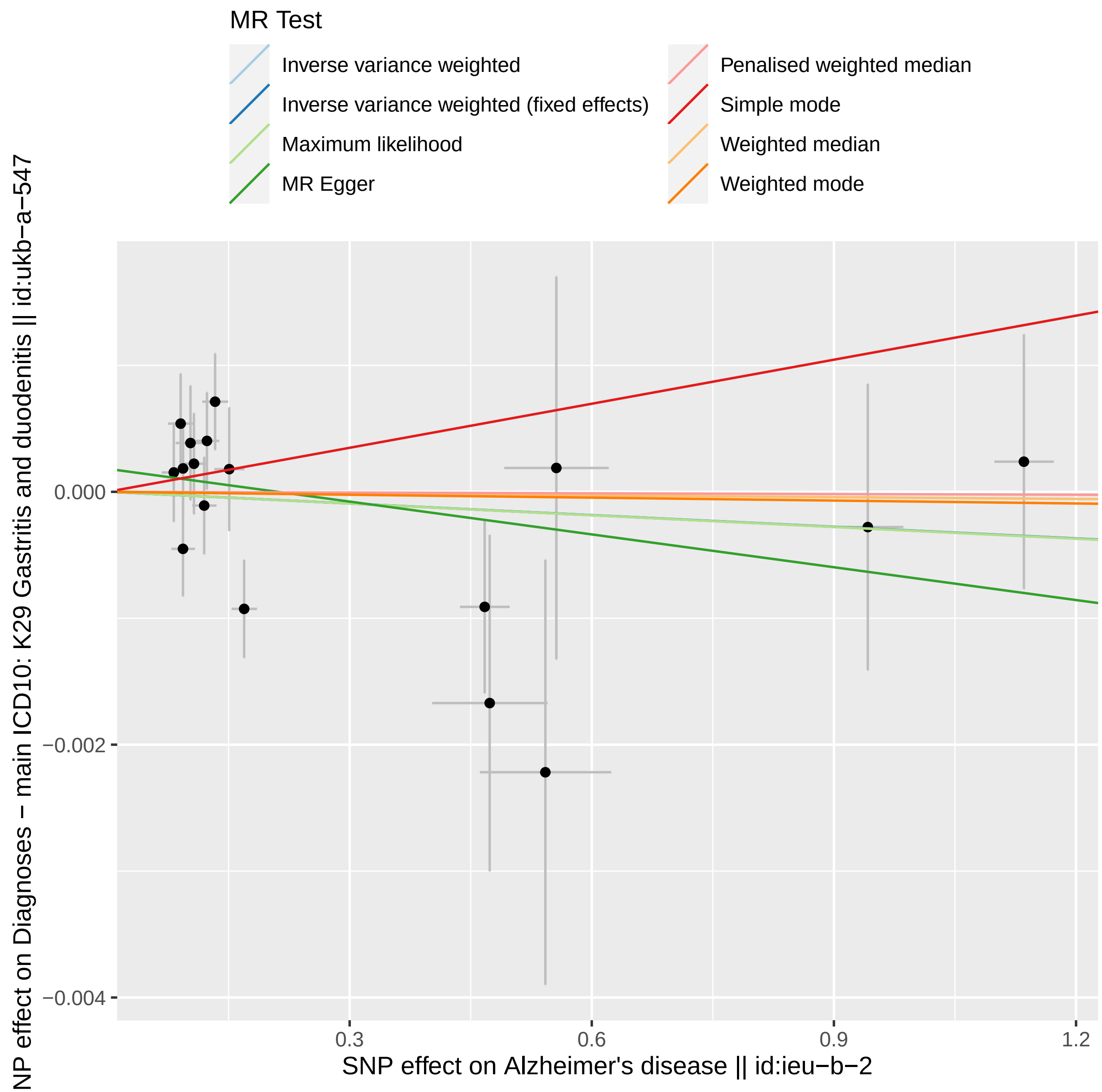

MR Test
Inverse variance weighted
Inverse variance weighted (fixed effects)
Maximum likelihood
MR Egger
Penalised weighted median
Simple mode
Weighted median
Weighted mode
0.000
−0.002
−0.004
0.3
0.6
0.9
1.2
SNP effect on Alzheimer's disease || id:ieu−b−2
NP effect on Diagnoses − main ICD10: K29 Gastritis and duodenitis || id:ukb−a−547

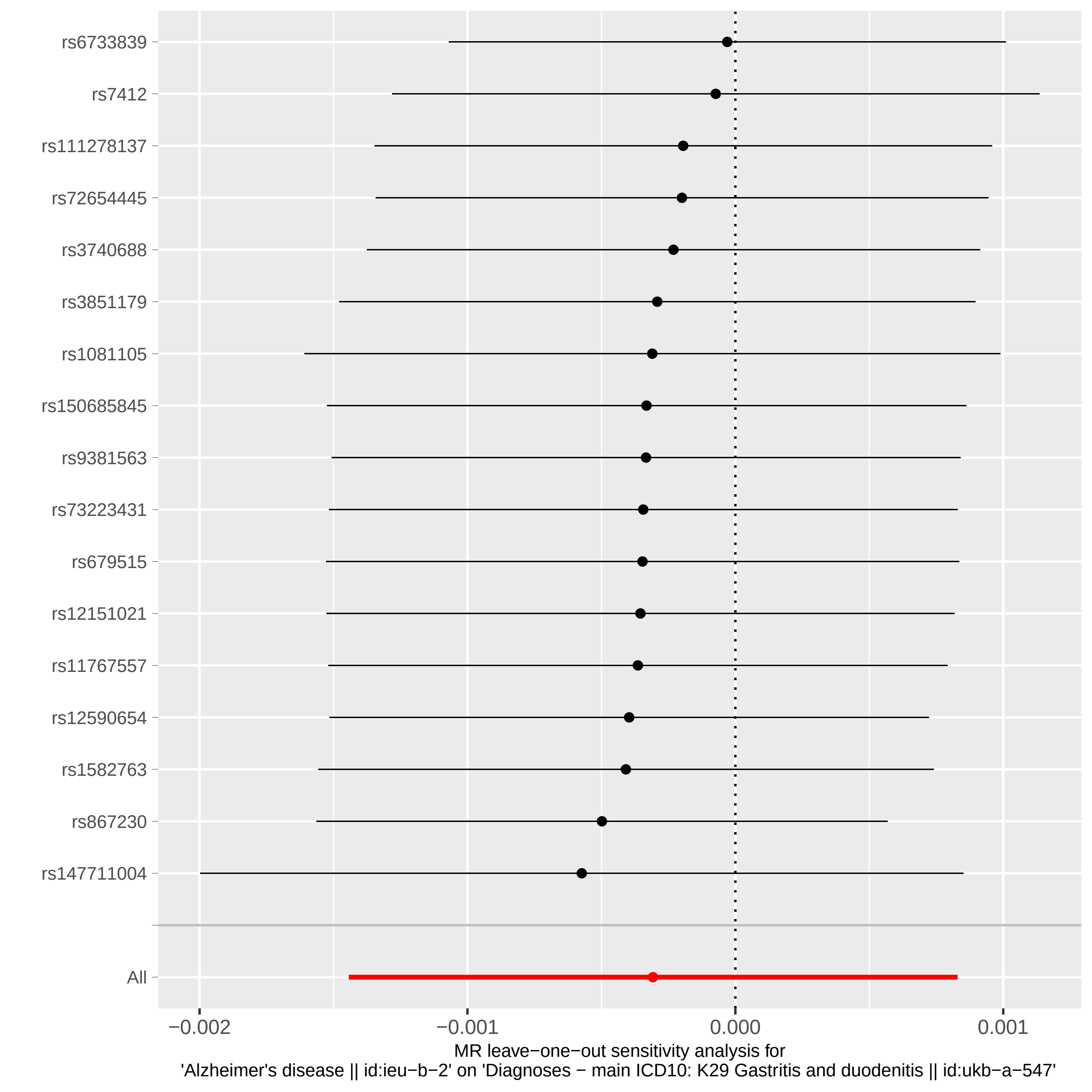
rs6733839
rs7412
rs111278137
rs72654445
rs3740688
rs3851179
rs1081105
rs150685845
rs9381563
rs73223431
rs679515
rs12151021
rs11767557
rs12590654
rs1582763
rs867230
rs147711004
All
−0.002
−0.001
0.000
0.001
MR leave−one−out sensitivity analysis for
'Alzheimer's disease || id:ieu−b−2' on 'Diagnoses − main ICD10: K29 Gastritis and duodenitis || id:ukb−a−547'

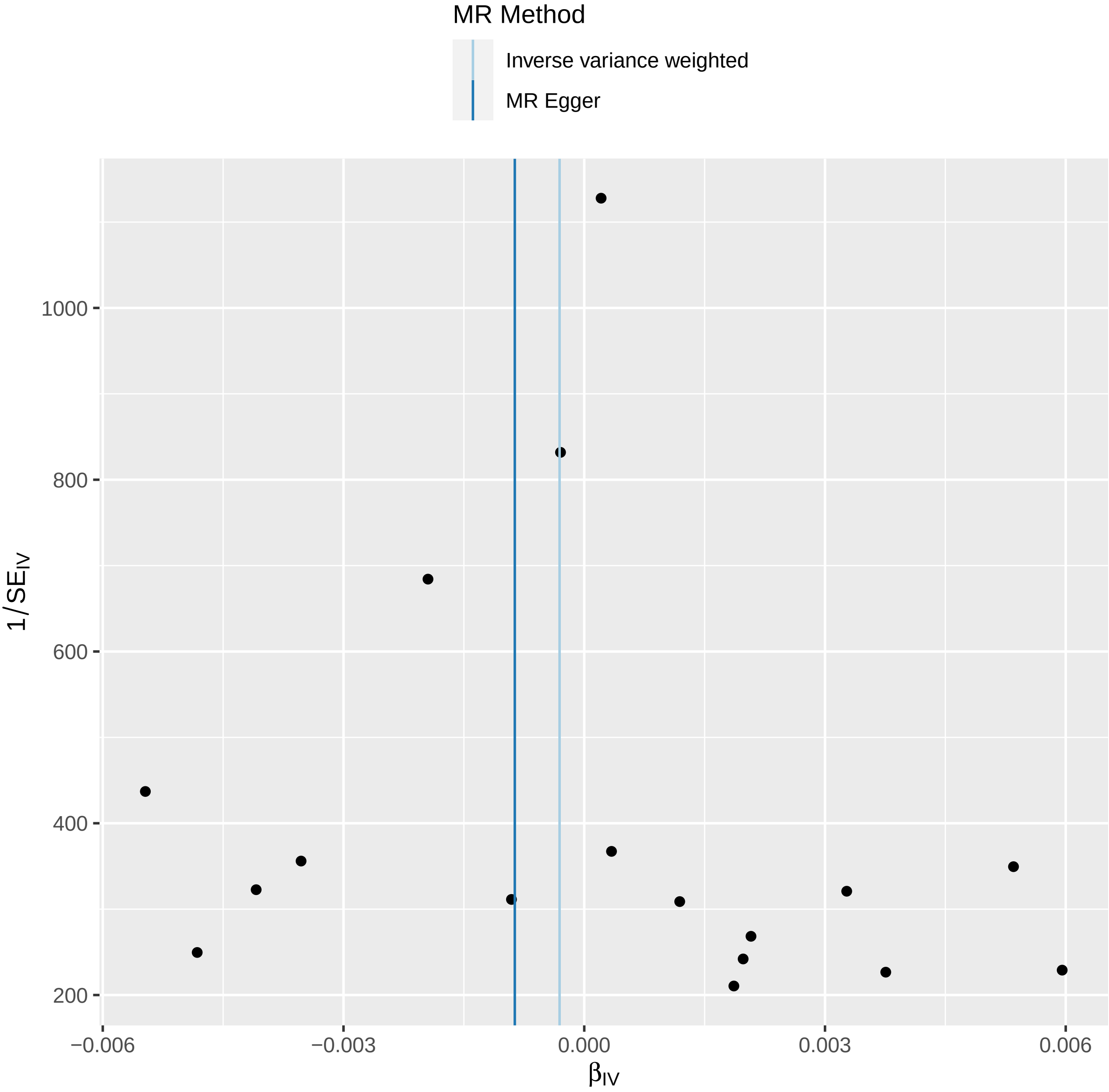
MR Method
Inverse variance weighted
MR Egger
1000
800
600
400
200
1/SE_IV
−0.006
−0.003
0.000
0.003
0.006
β_IV

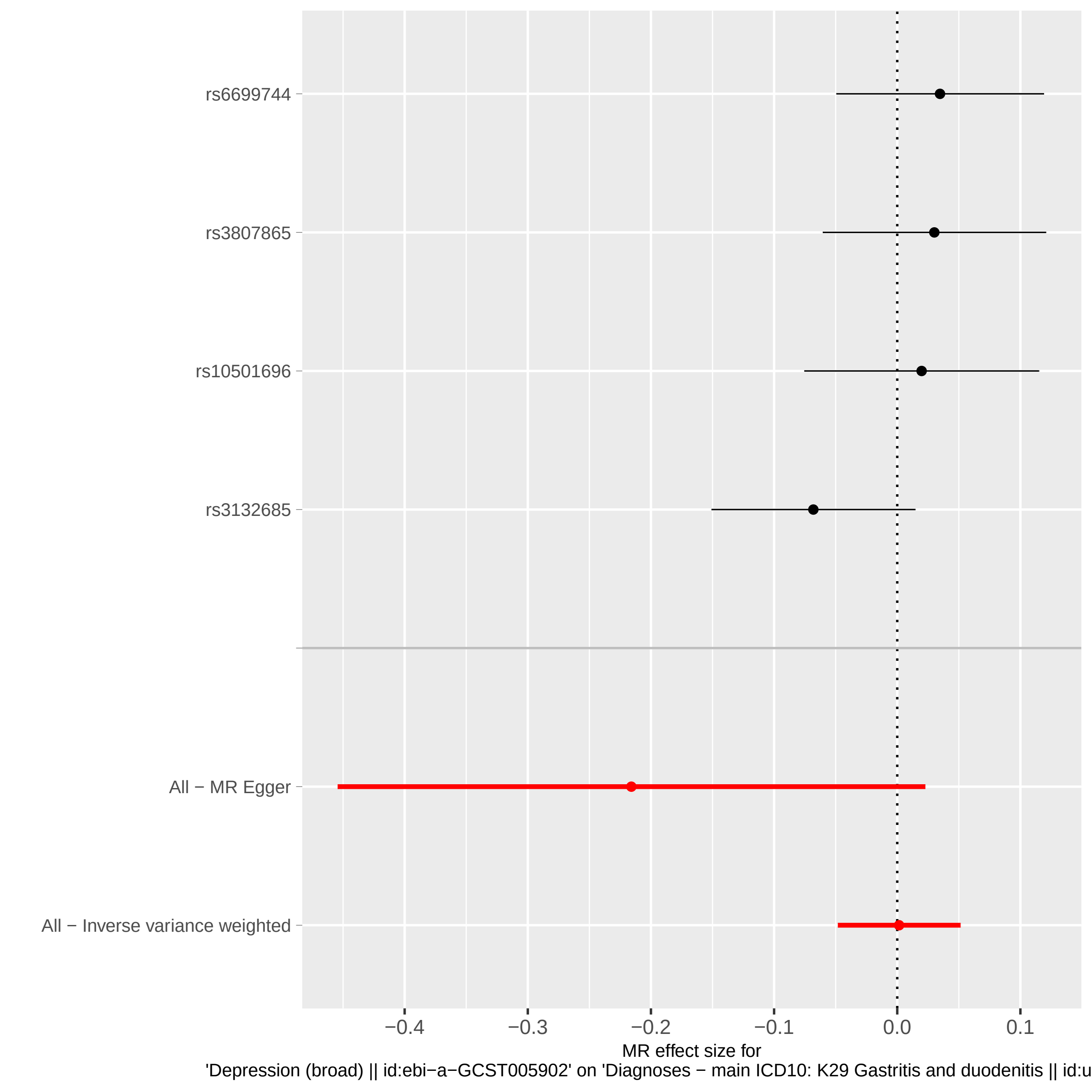
rs6699744
rs3807865
rs10501696
rs3132685
All − MR Egger
All − Inverse variance weighted
−0.4
−0.3
−0.2
−0.1
0.0
0.1
MR effect size for
'Depression (broad) || id:ebi−a−GCST005902' on 'Diagnoses − main ICD10: K29 Gastritis and duodenitis || id:u

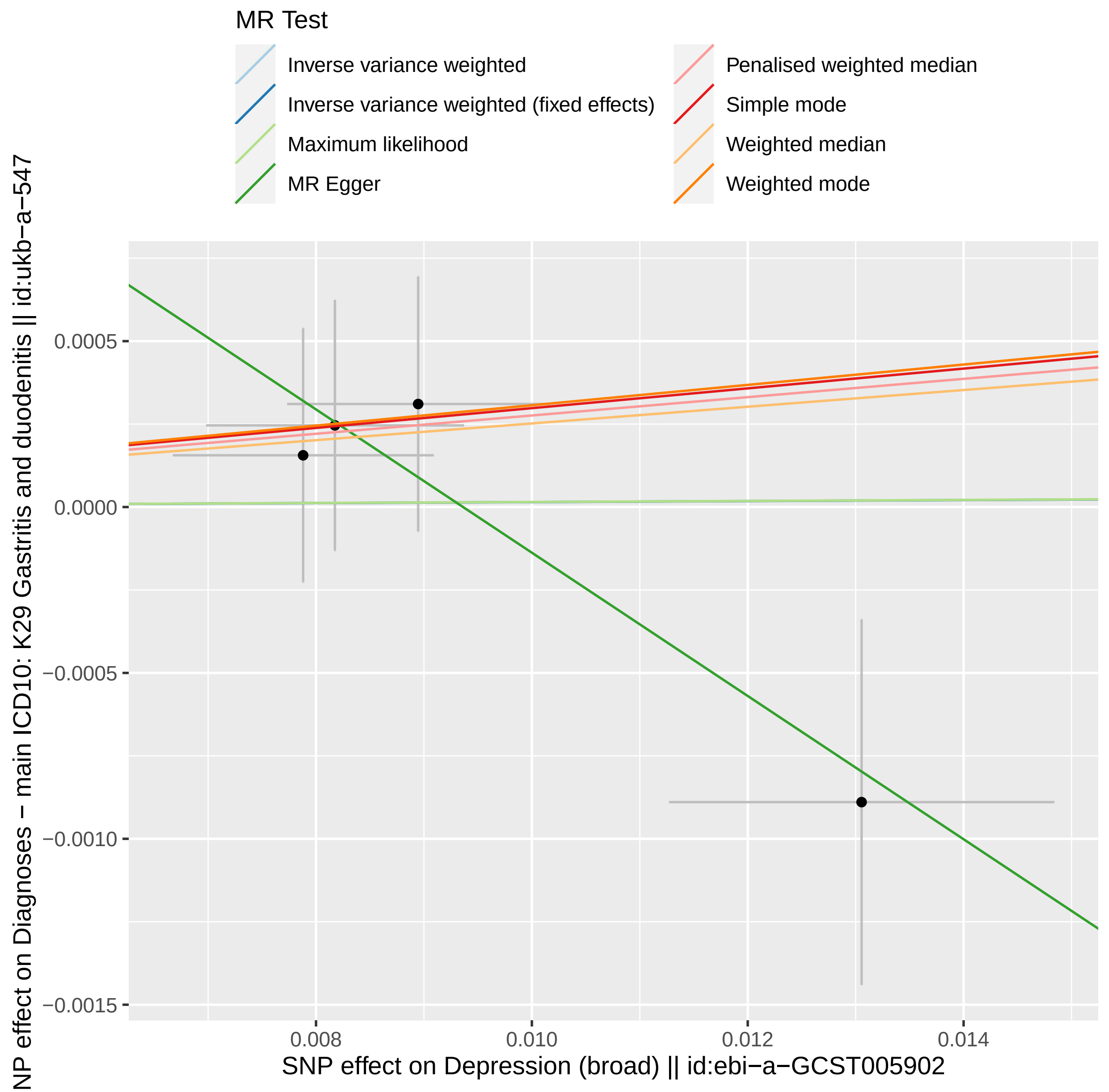

MR Test
Inverse variance weighted
Inverse variance weighted (fixed effects)
Maximum likelihood
MR Egger
Penalised weighted median
Simple mode
Weighted median
Weighted mode
0.0005
0.0000
−0.0005
−0.0010
−0.0015
0.008
0.010
0.012
0.014
SNP effect on Depression (broad) || id:ebi−a−GCST005902
NP effect on Diagnoses − main ICD10: K29 Gastritis and duodenitis || id:ukb−a−547

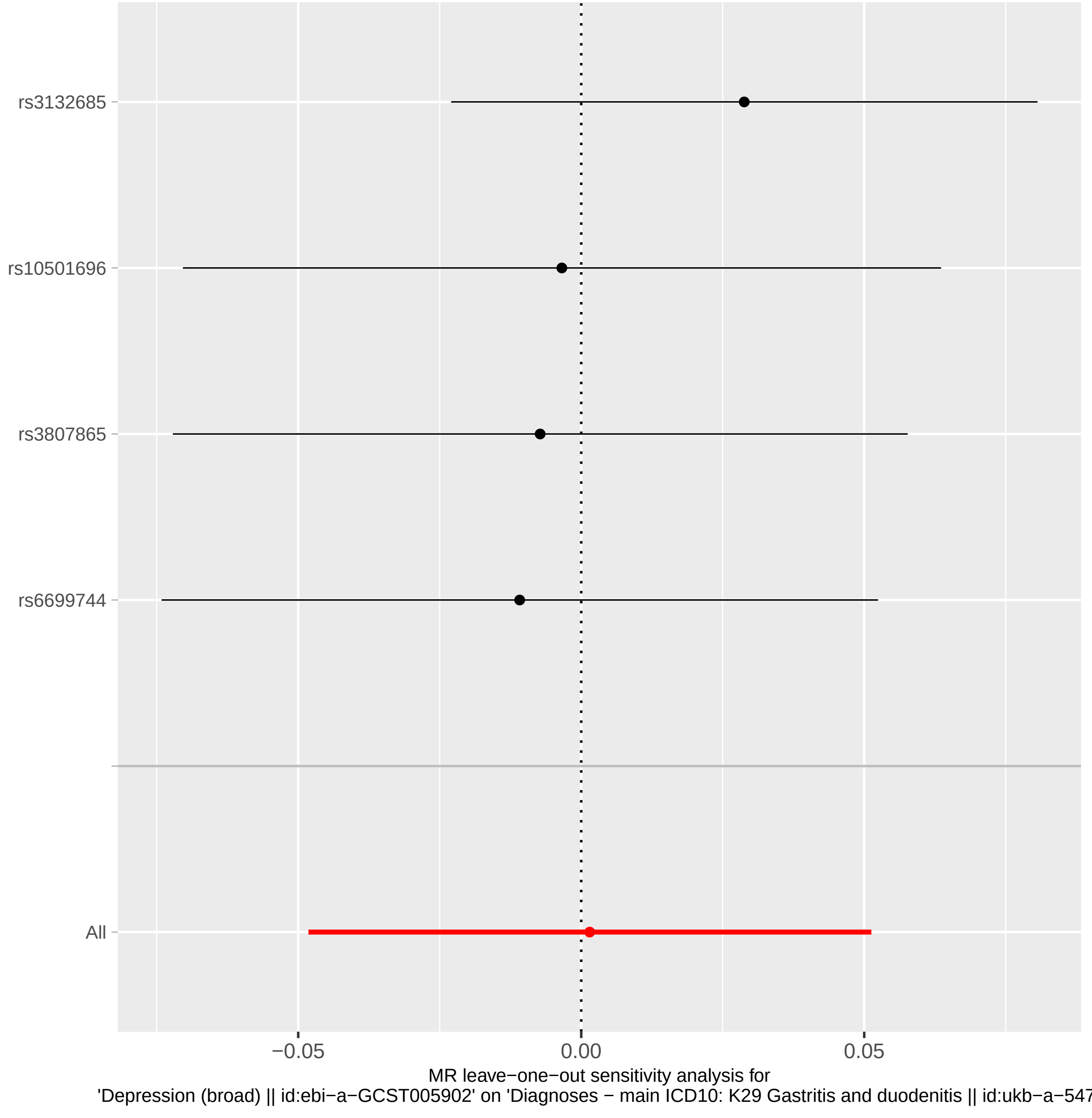

rs3132685
rs10501696
rs3807865
rs6699744
All
−0.05
0.00
0.05
MR leave−one−out sensitivity analysis for
'Depression (broad) || id:ebi−a−GCST005902' on 'Diagnoses − main ICD10: K29 Gastritis and duodenitis || id:ukb−a−547

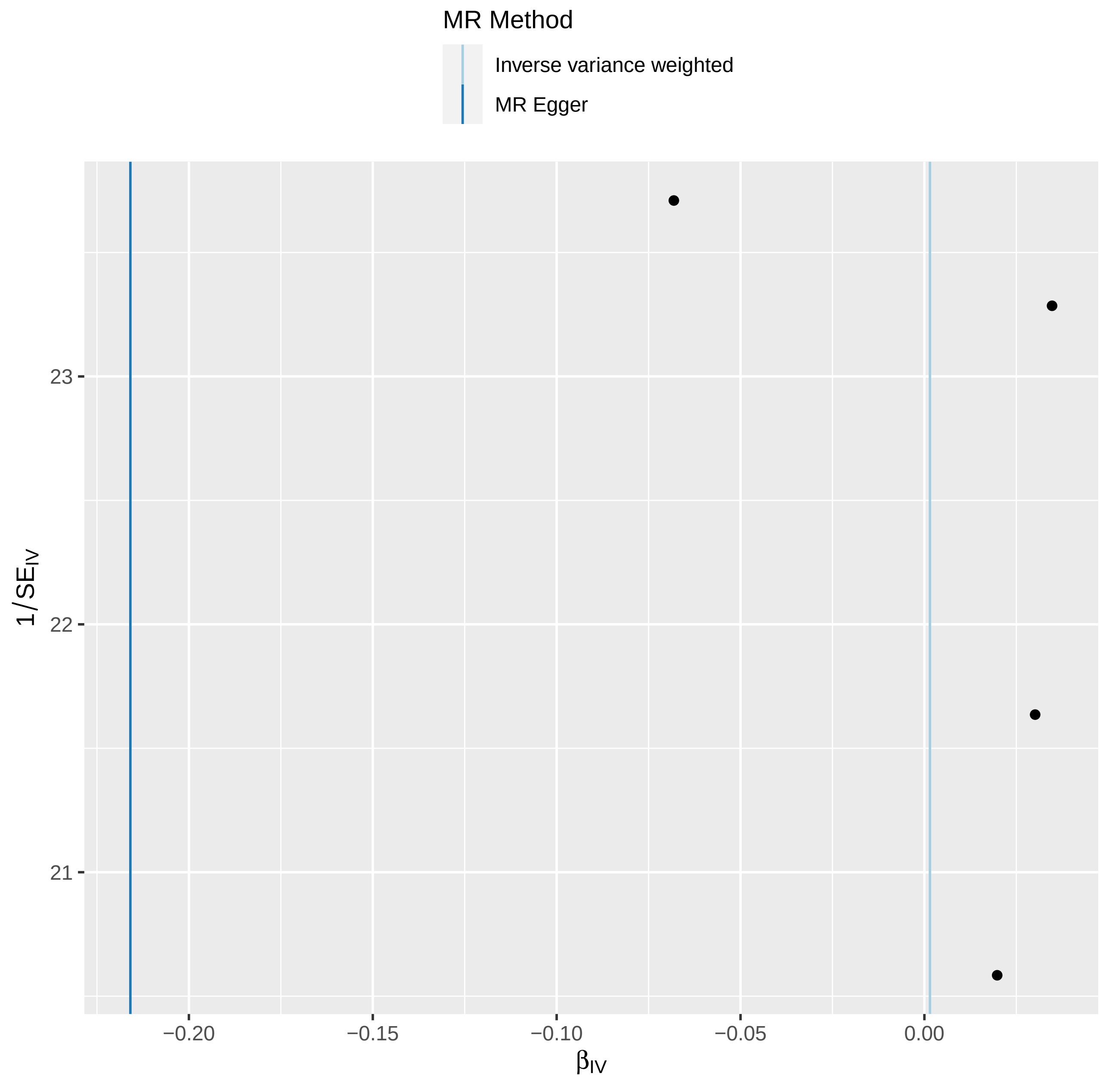

MR Method
Inverse variance weighted
MR Egger
23
22
21
1/SE_IV
−0.20
−0.15
−0.10
−0.05
0.00
β_IV

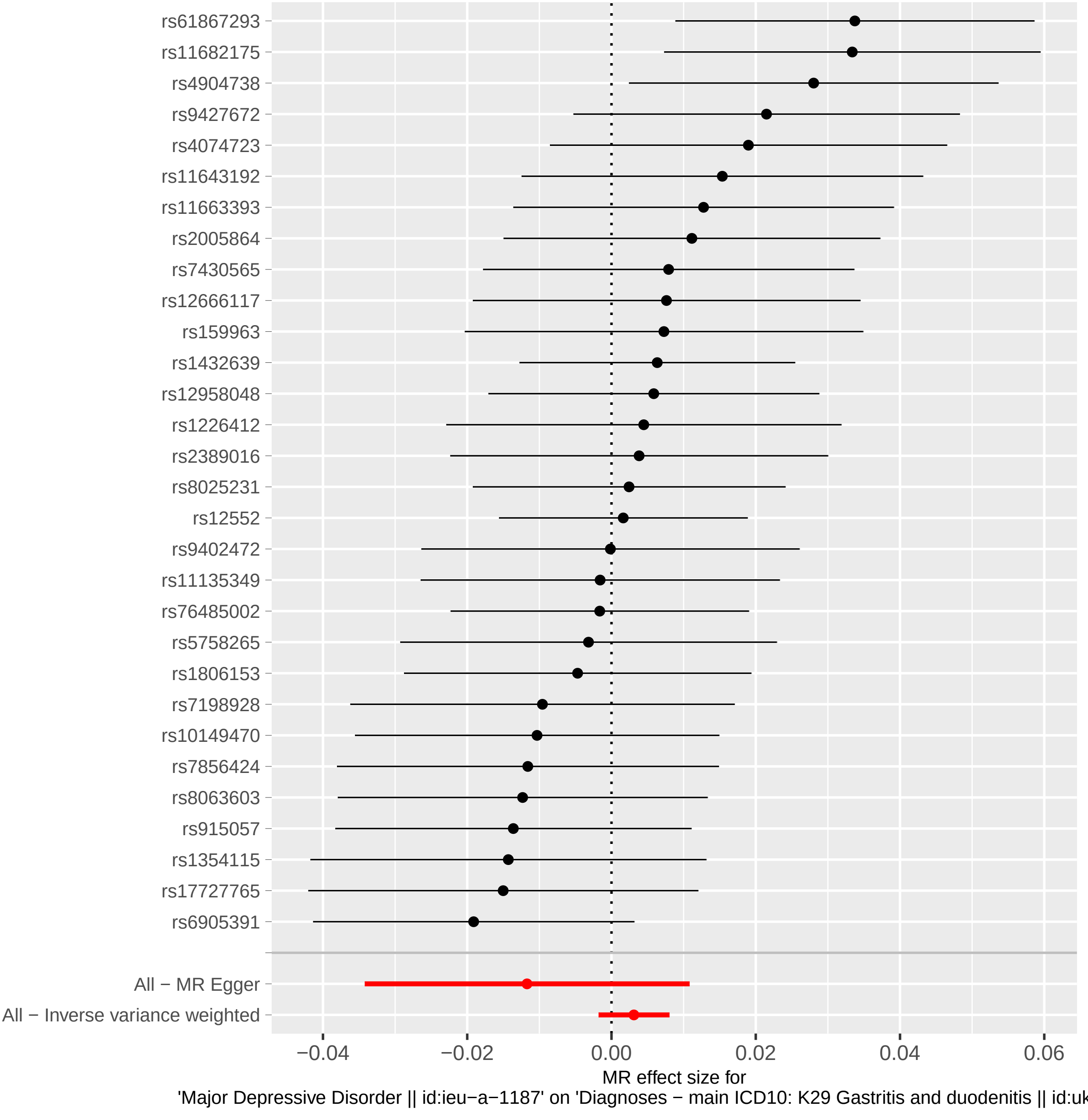

rs61867293
rs11682175
rs4904738
rs9427672
rs4074723
rs11643192
rs11663393
rs2005864
rs7430565
rs12666117
rs159963
rs1432639
rs12958048
rs1226412
rs2389016
rs8025231
rs12552
rs9402472
rs11135349
rs76485002
rs5758265
rs1806153
rs7198928
rs10149470
rs7856424
rs8063603
rs915057
rs1354115
rs17727765
rs6905391
All − MR Egger
All − Inverse variance weighted
−0.04
−0.02
0.00
0.02
0.04
0.06
MR effect size for
'Major Depressive Disorder || id:ieu−a−1187' on 'Diagnoses − main ICD10: K29 Gastritis and duodenitis || id:uk

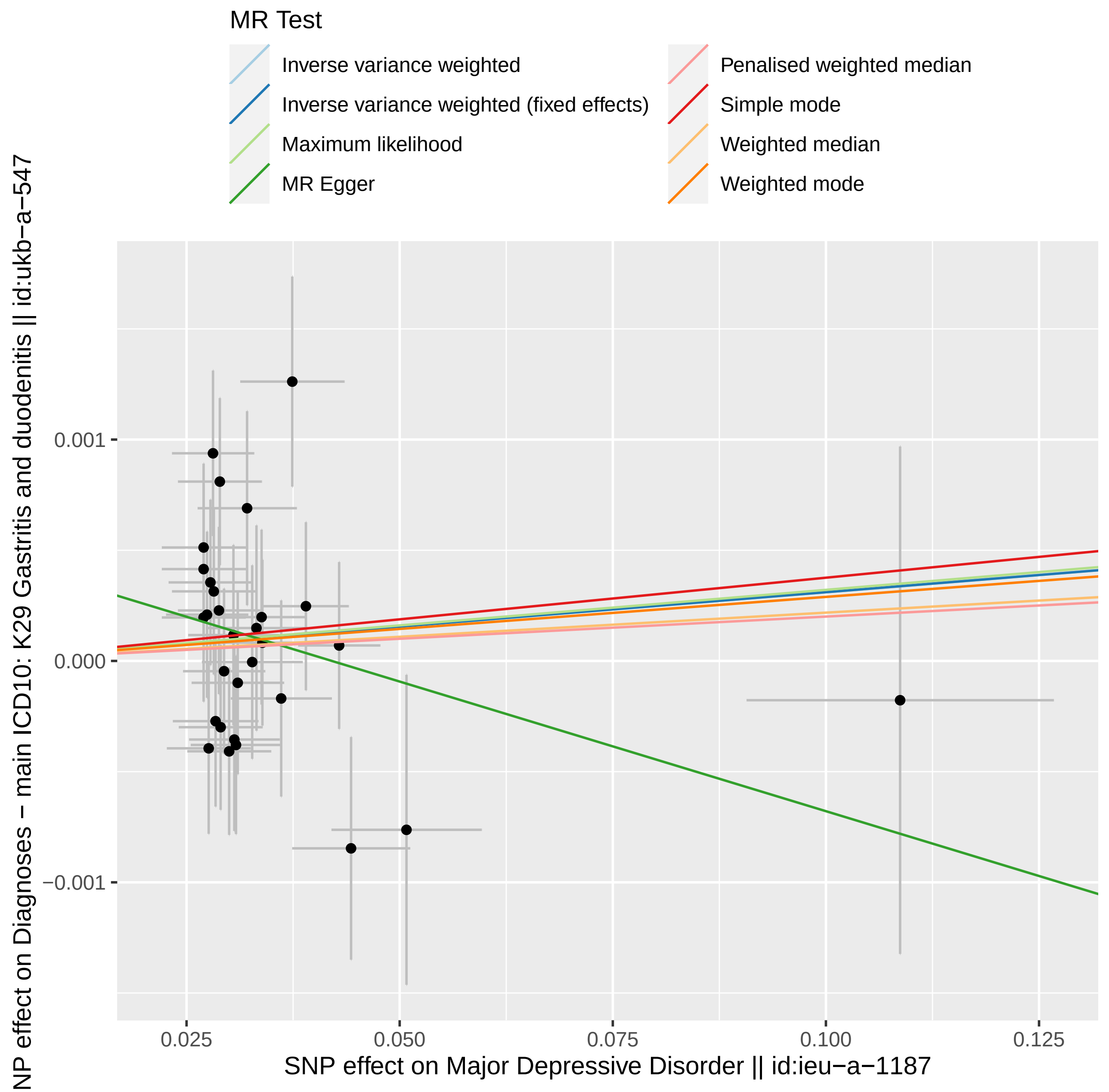

MR Test
Inverse variance weighted
Inverse variance weighted (fixed effects)
Maximum likelihood
MR Egger
Penalised weighted median
Simple mode
Weighted median
Weighted mode
0.001
0.000
−0.001
0.025
0.050
0.075
0.100
0.125
SNP effect on Major Depressive Disorder || id:ieu−a−1187
NP effect on Diagnoses − main ICD10: K29 Gastritis and duodenitis || id:ukb−a−547

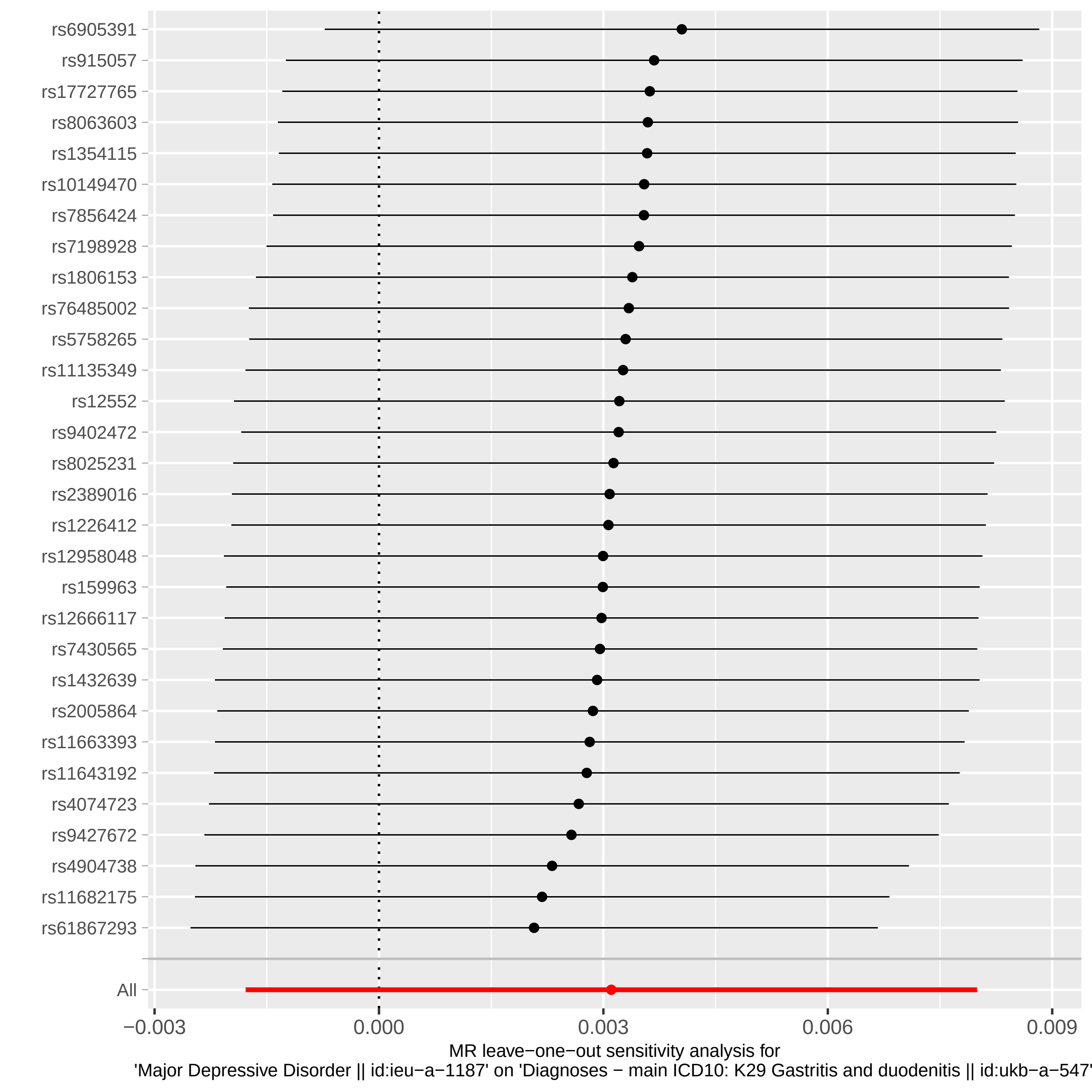
rs6905391
rs915057
rs17727765
rs8063603
rs1354115
rs10149470
rs7856424
rs7198928
rs1806153
rs76485002
rs5758265
rs11135349
rs12552
rs9402472
rs8025231
rs2389016
rs1226412
rs12958048
rs159963
rs12666117
rs7430565
rs1432639
rs2005864
rs11663393
rs11643192
rs4074723
rs9427672
rs4904738
rs11682175
rs61867293
All
−0.003
0.000
0.003
0.006
0.009
MR leave−one−out sensitivity analysis for
'Major Depressive Disorder || id:ieu−a−1187' on 'Diagnoses − main ICD10: K29 Gastritis and duodenitis || id:ukb−a−547'

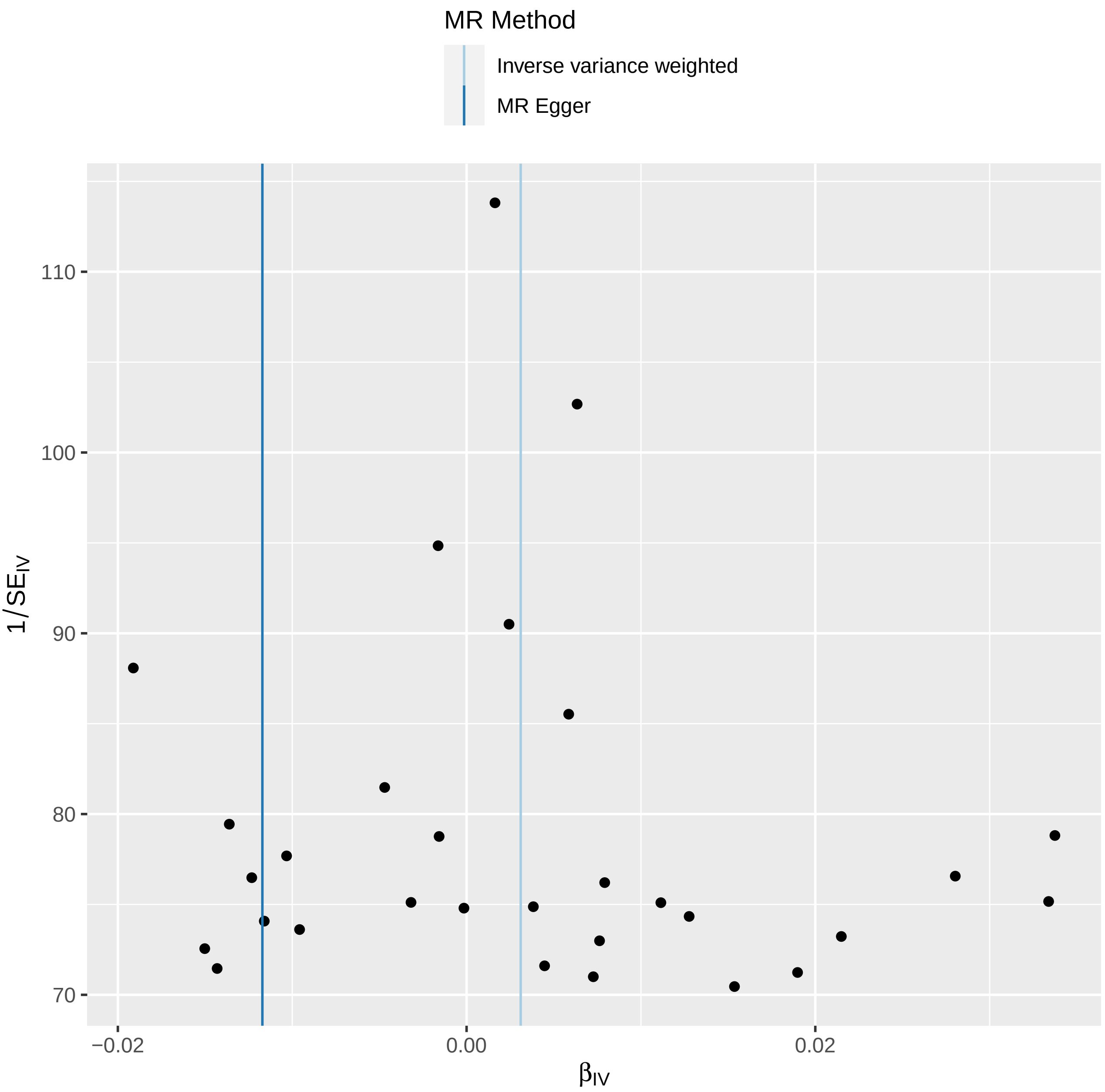

MR Method
Inverse variance weighted
MR Egger
110
100
90
80
70
1/SE$_{IV}$
−0.02
0.00
0.02
β$_{IV}$

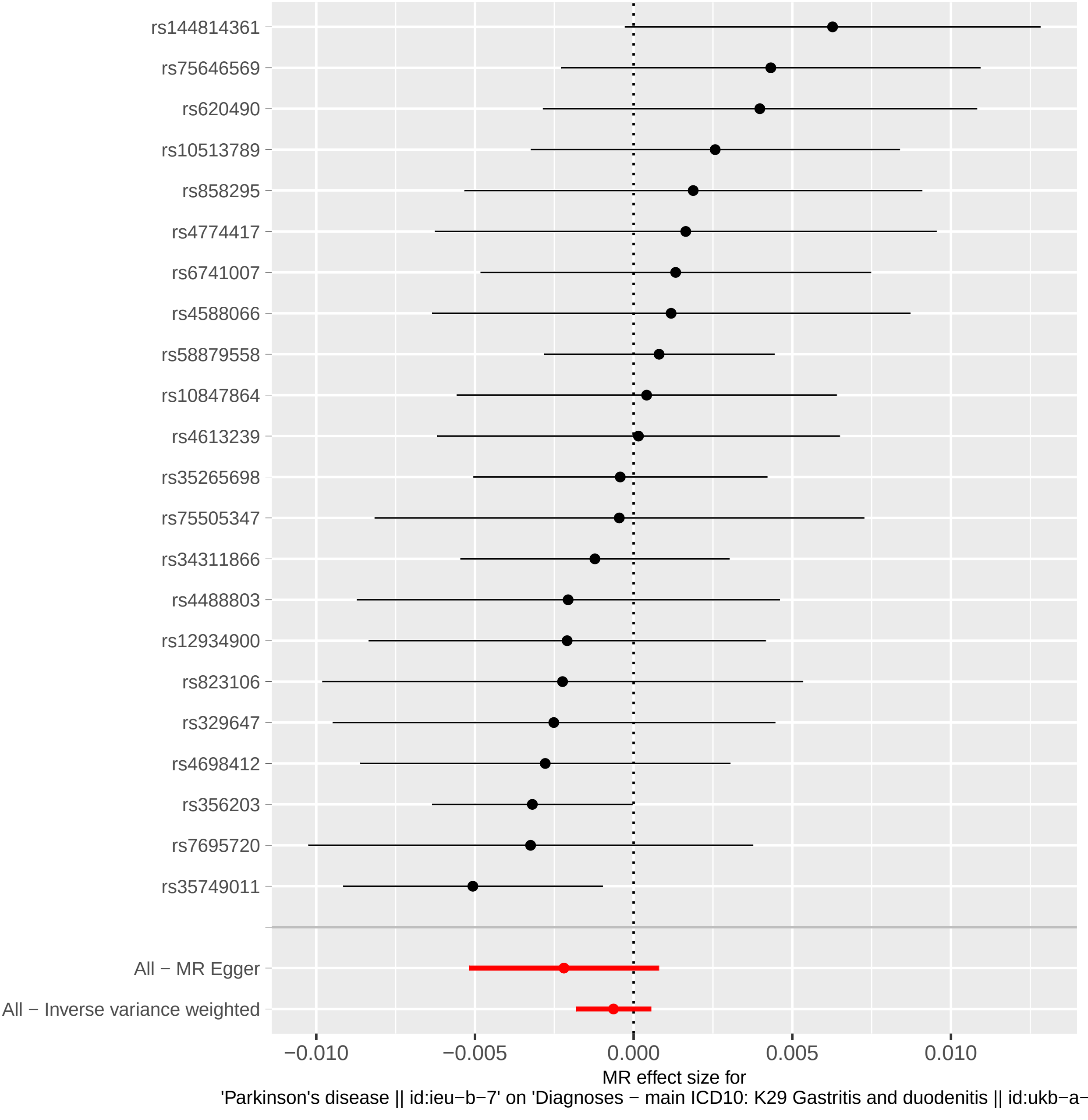
rs144814361
rs75646569
rs620490
rs10513789
rs858295
rs4774417
rs6741007
rs4588066
rs58879558
rs10847864
rs4613239
rs35265698
rs75505347
rs34311866
rs4488803
rs12934900
rs823106
rs329647
rs4698412
rs356203
rs7695720
rs35749011
All − MR Egger
All − Inverse variance weighted
−0.010
−0.005
0.000
0.005
0.010
MR effect size for
'Parkinson's disease || id:ieu−b−7' on 'Diagnoses − main ICD10: K29 Gastritis and duodenitis || id:ukb−a−

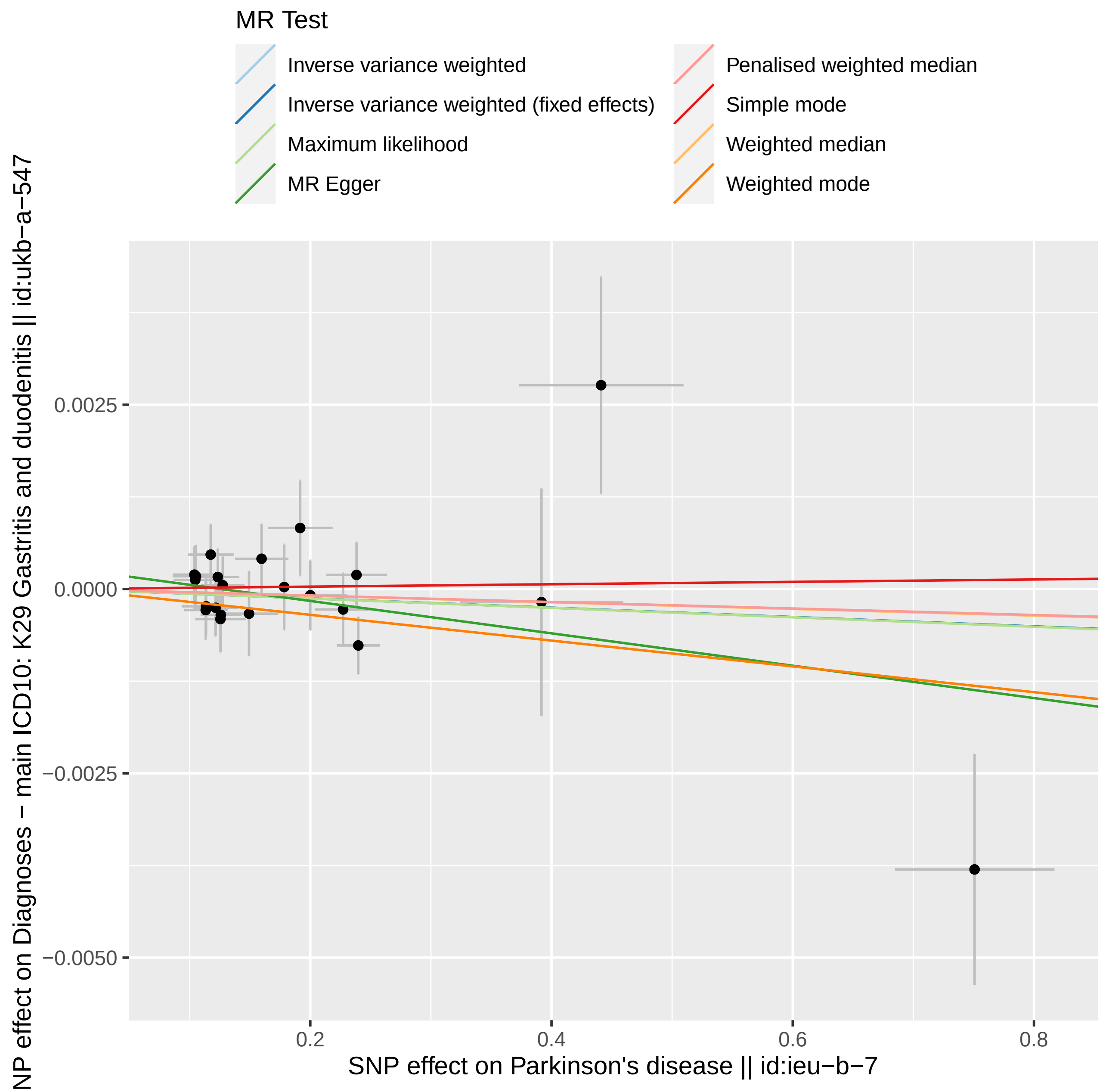
MR Test
Inverse variance weighted
Inverse variance weighted (fixed effects)
Maximum likelihood
MR Egger
Penalised weighted median
Simple mode
Weighted median
Weighted mode
0.0025
0.0000
−0.0025
−0.0050
0.2
0.4
0.6
0.8
SNP effect on Parkinson's disease || id:ieu−b−7
NP effect on Diagnoses − main ICD10: K29 Gastritis and duodenitis || id:ukb−a−547

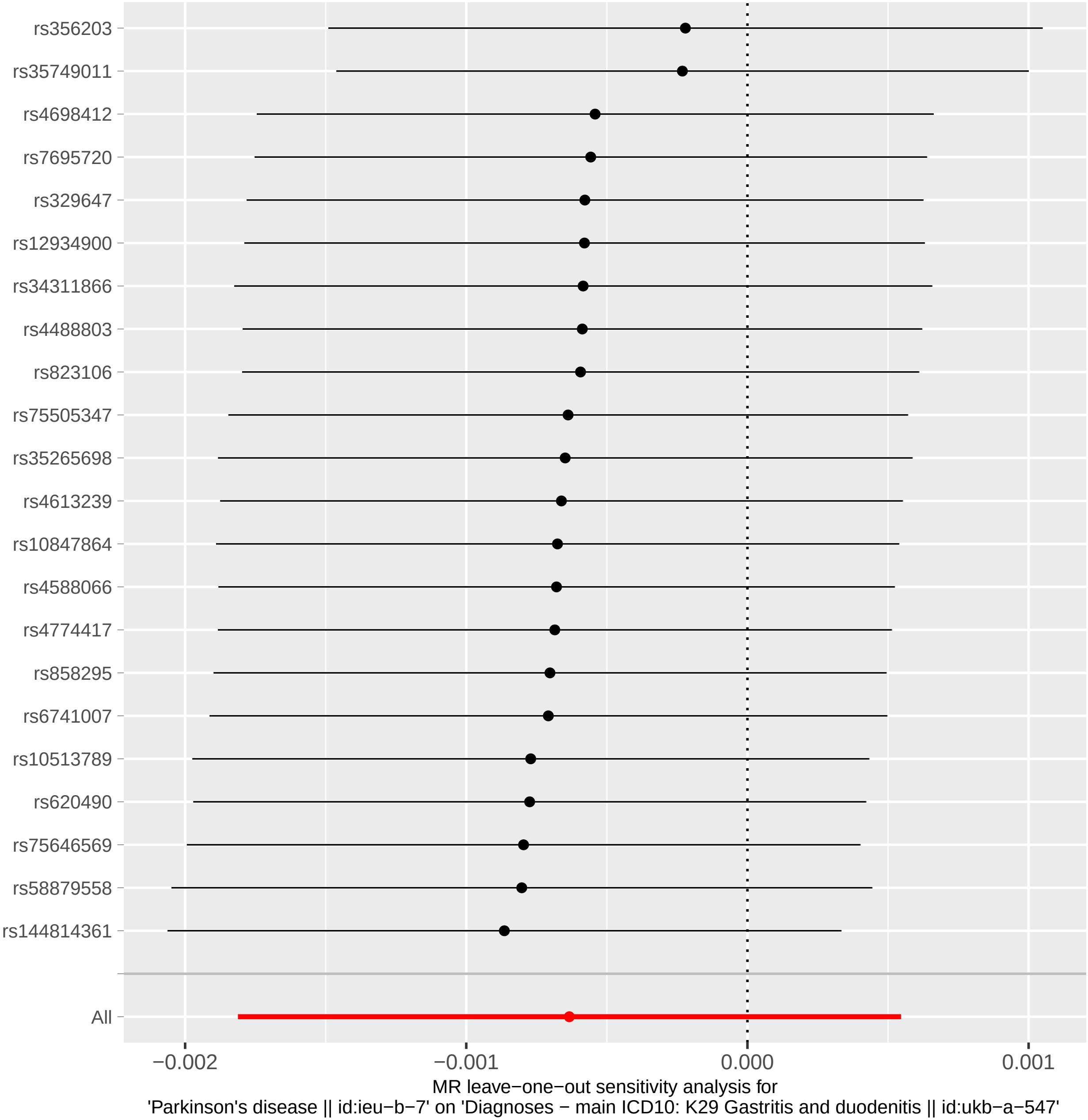
rs356203
rs35749011
rs4698412
rs7695720
rs329647
rs12934900
rs34311866
rs4488803
rs823106
rs75505347
rs35265698
rs4613239
rs10847864
rs4588066
rs4774417
rs858295
rs6741007
rs10513789
rs620490
rs75646569
rs58879558
rs144814361
All
−0.002
−0.001
0.000
0.001
MR leave−one−out sensitivity analysis for
'Parkinson's disease || id:ieu−b−7' on 'Diagnoses − main ICD10: K29 Gastritis and duodenitis || id:ukb−a−547'

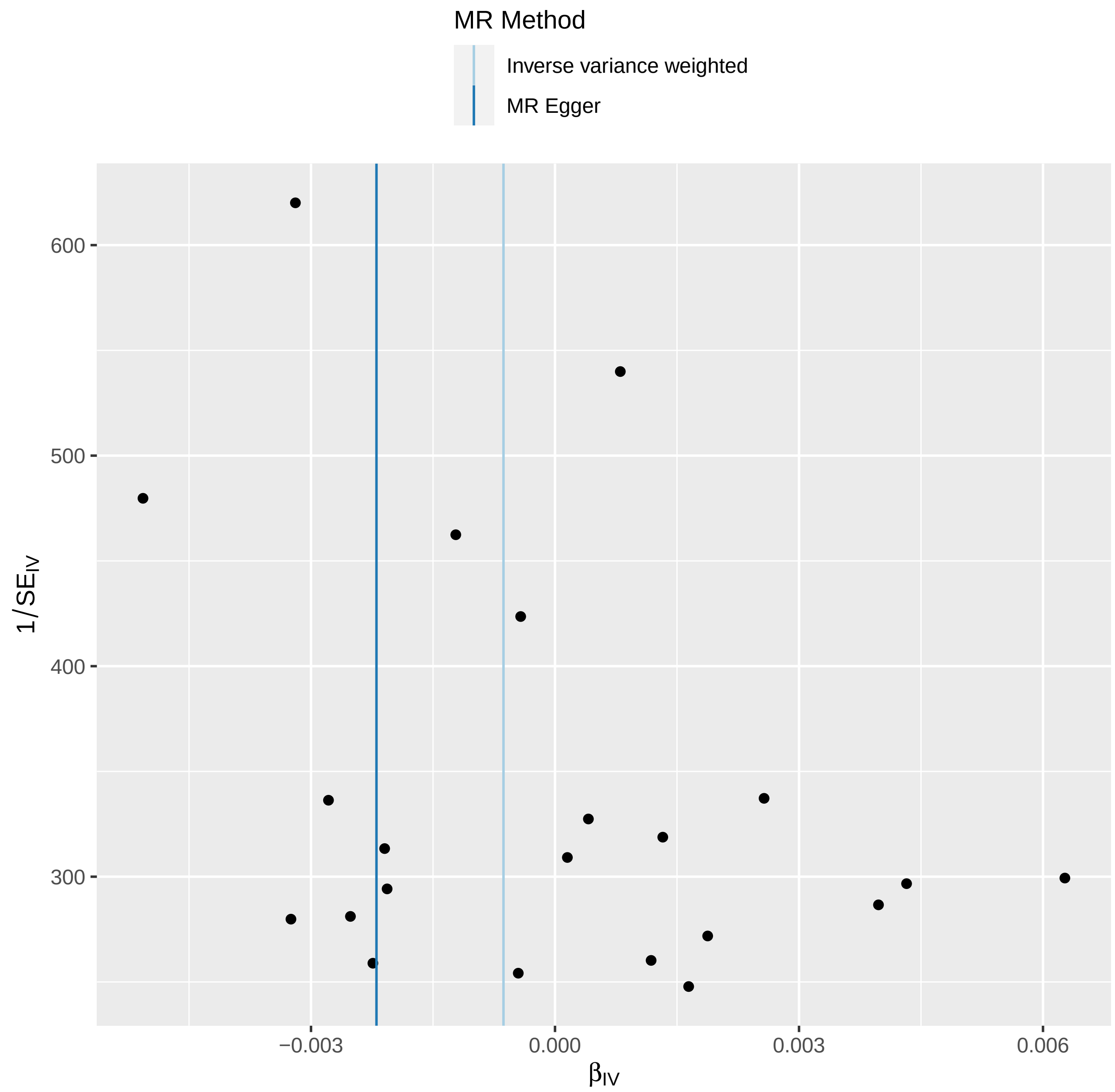

MR Method
Inverse variance weighted
MR Egger
600
500
400
300
1/SE_IV
−0.003
0.000
0.003
0.006
β_IV

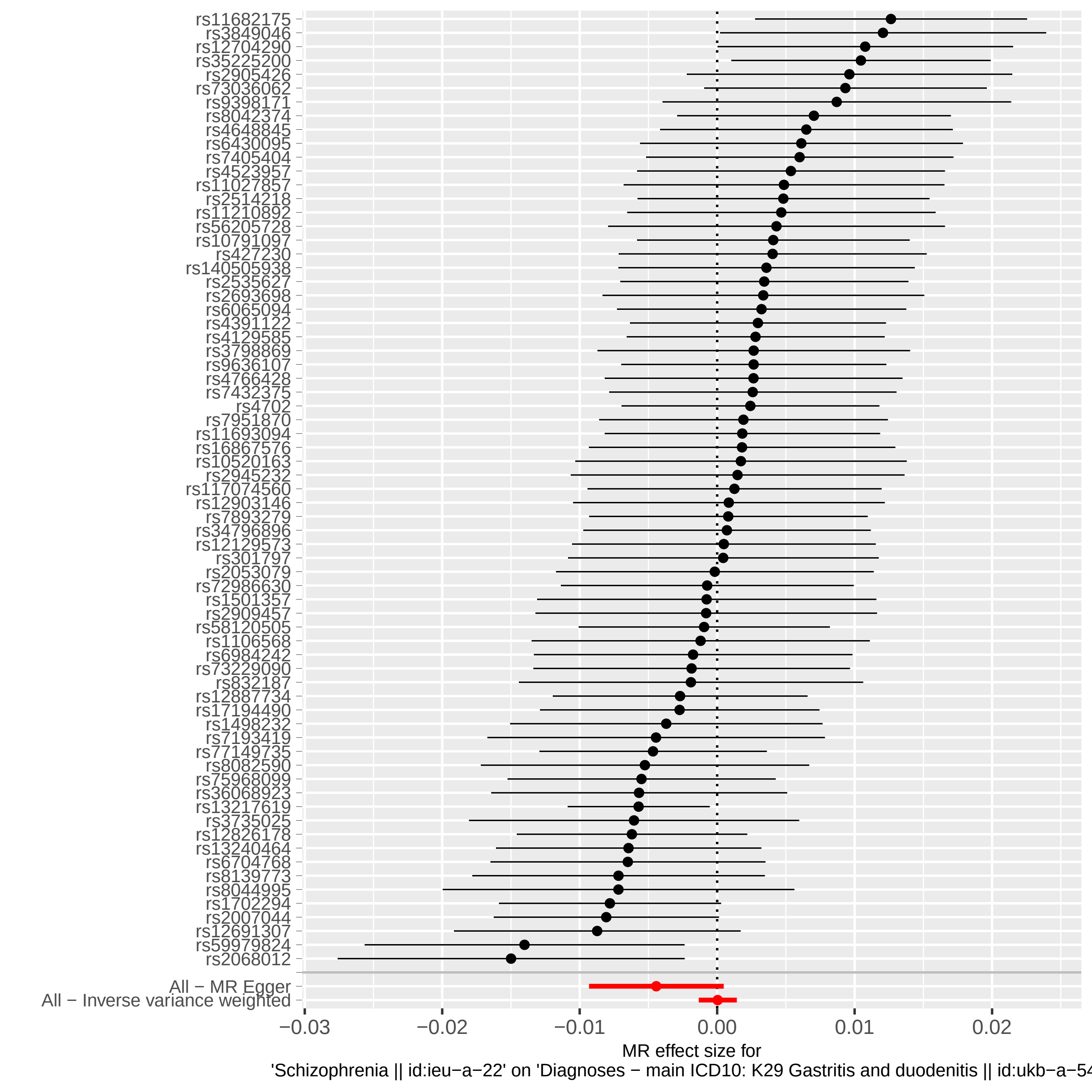

rs11682175
rs3849046
rs12704290
rs35225200
rs2905426
rs73036062
rs9398171
rs8042374
rs4648845
rs6430095
rs7405404
rs4523957
rs11027857
rs2514218
rs11210892
rs56205728
rs10791097
rs427230
rs140505938
rs2535627
rs2693698
rs6065094
rs4391122
rs4129585
rs3798869
rs9636107
rs4766428
rs7432375
rs4702
rs7951870
rs11693094
rs16867576
rs10520163
rs2945232
rs117074560
rs12903146
rs7893279
rs34796896
rs12129573
rs301797
rs2053079
rs72986630
rs1501357
rs2909457
rs58120505
rs1106568
rs6984242
rs73229090
rs832187
rs12887734
rs17194490
rs1498232
rs7193419
rs77149735
rs8082590
rs75968099
rs36068923
rs13217619
rs3735025
rs12826178
rs13240464
rs6704768
rs8139773
rs8044995
rs1702294
rs2007044
rs12691307
rs59979824
rs2068012
All − MR Egger
All − Inverse variance weighted
−0.03
−0.02
−0.01
0.00
0.01
0.02
MR effect size for
'Schizophrenia || id:ieu−a−22' on 'Diagnoses − main ICD10: K29 Gastritis and duodenitis || id:ukb−a−54

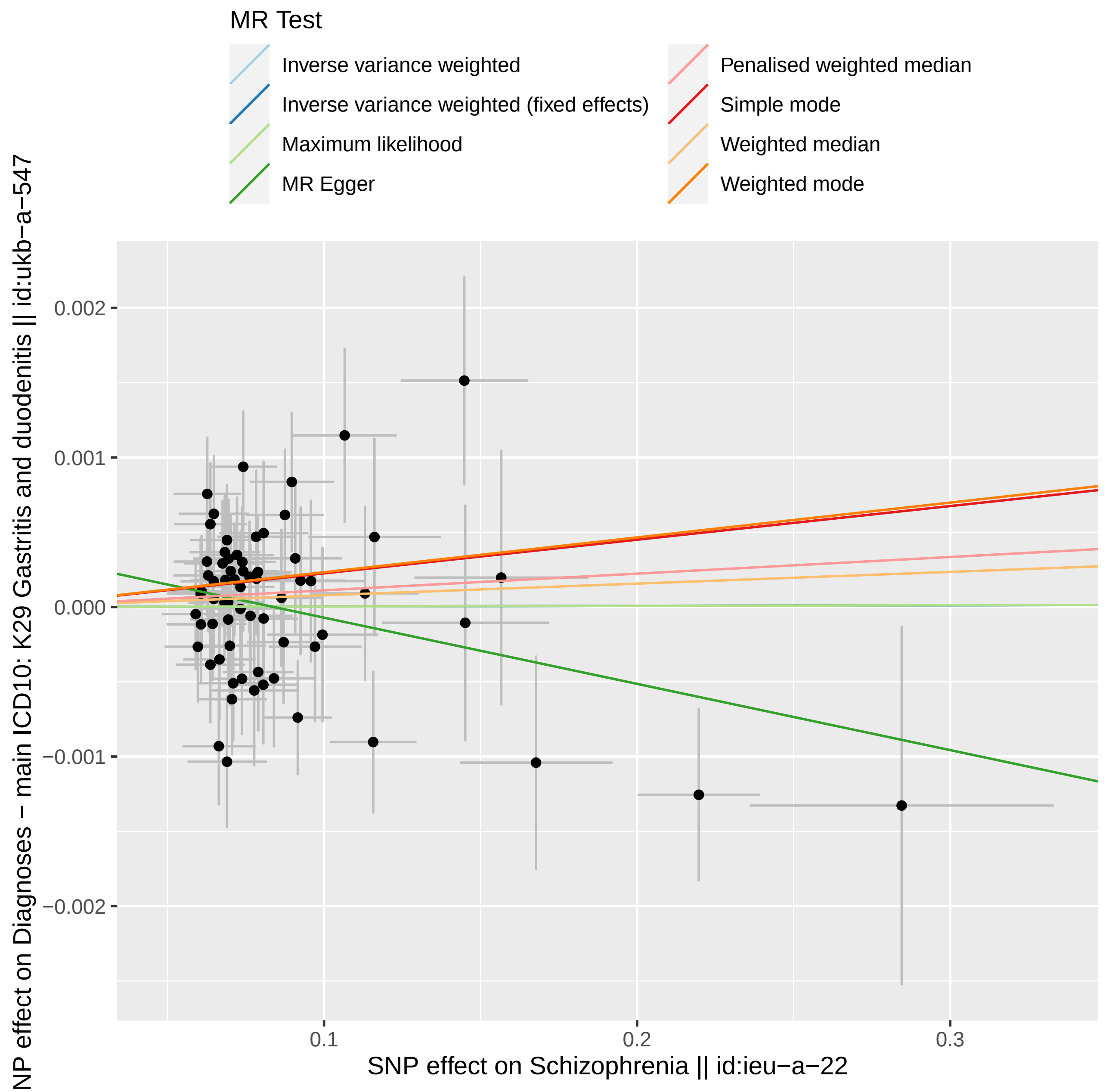
MR Test
Inverse variance weighted
Inverse variance weighted (fixed effects)
Maximum likelihood
MR Egger
Penalised weighted median
Simple mode
Weighted median
Weighted mode
0.002
0.001
0.000
−0.001
−0.002
0.1
0.2
0.3
SNP effect on Schizophrenia || id:ieu−a−22
NP effect on Diagnoses − main ICD10: K29 Gastritis and duodenitis || id:ukb−a−547

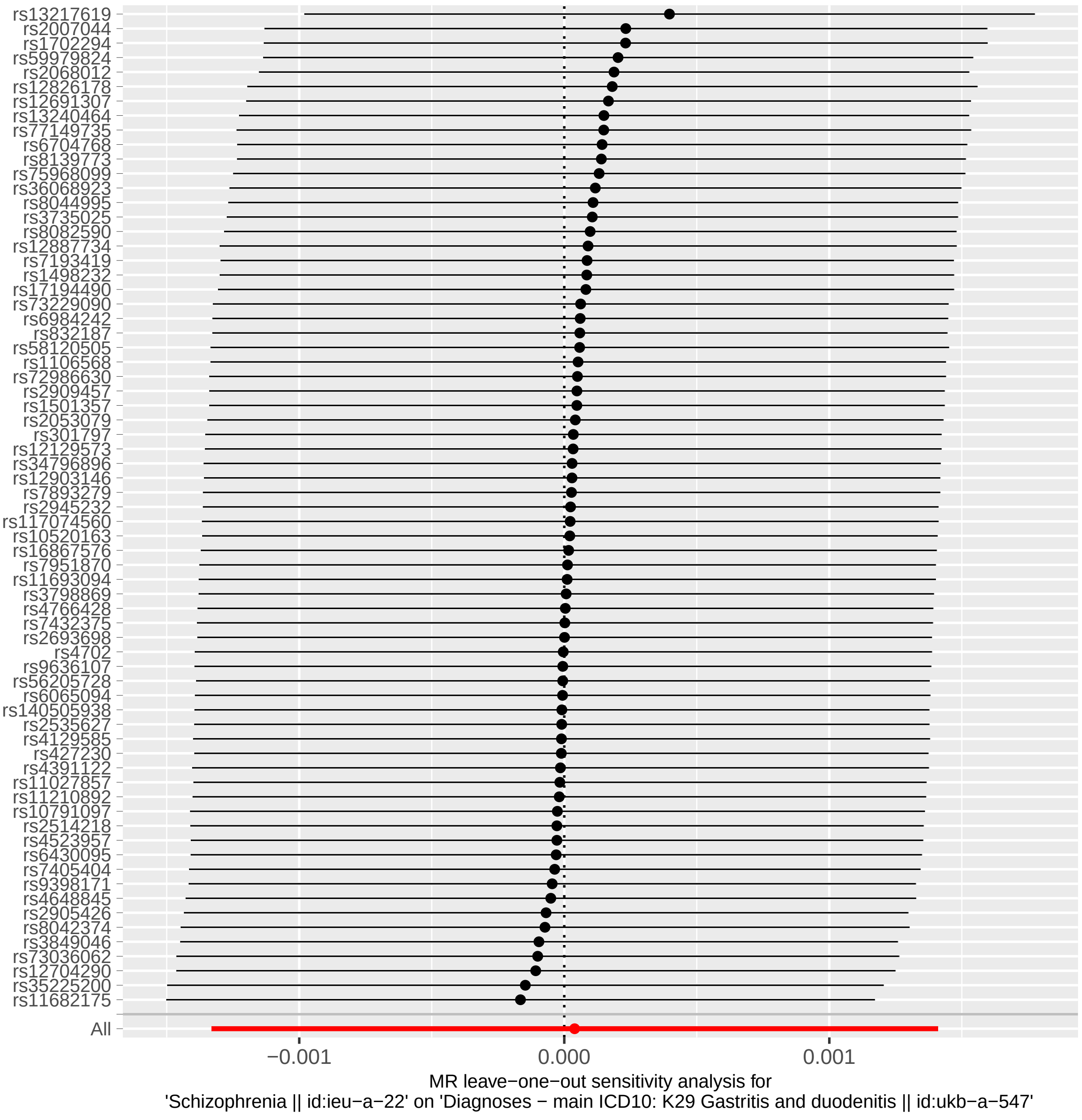

rs13217619
rs2007044
rs1702294
rs59979824
rs2068012
rs12826178
rs12691307
rs13240464
rs77149735
rs6704768
rs8139773
rs75968099
rs36068923
rs8044995
rs3735025
rs8082590
rs12887734
rs7193419
rs1498232
rs17194490
rs73229090
rs6984242
rs832187
rs58120505
rs1106568
rs72986630
rs2909457
rs1501357
rs2053079
rs301797
rs12129573
rs34796896
rs12903146
rs7893279
rs2945232
rs117074560
rs10520163
rs16867576
rs7951870
rs11693094
rs3798869
rs4766428
rs7432375
rs2693698
rs4702
rs9636107
rs56205728
rs6065094
rs140505938
rs2535627
rs4129585
rs427230
rs4391122
rs11027857
rs11210892
rs10791097
rs2514218
rs4523957
rs6430095
rs7405404
rs9398171
rs4648845
rs2905426
rs8042374
rs3849046
rs73036062
rs12704290
rs35225200
rs11682175
All
−0.001
0.000
0.001
MR leave−one−out sensitivity analysis for
'Schizophrenia || id:ieu−a−22' on 'Diagnoses − main ICD10: K29 Gastritis and duodenitis || id:ukb−a−547'

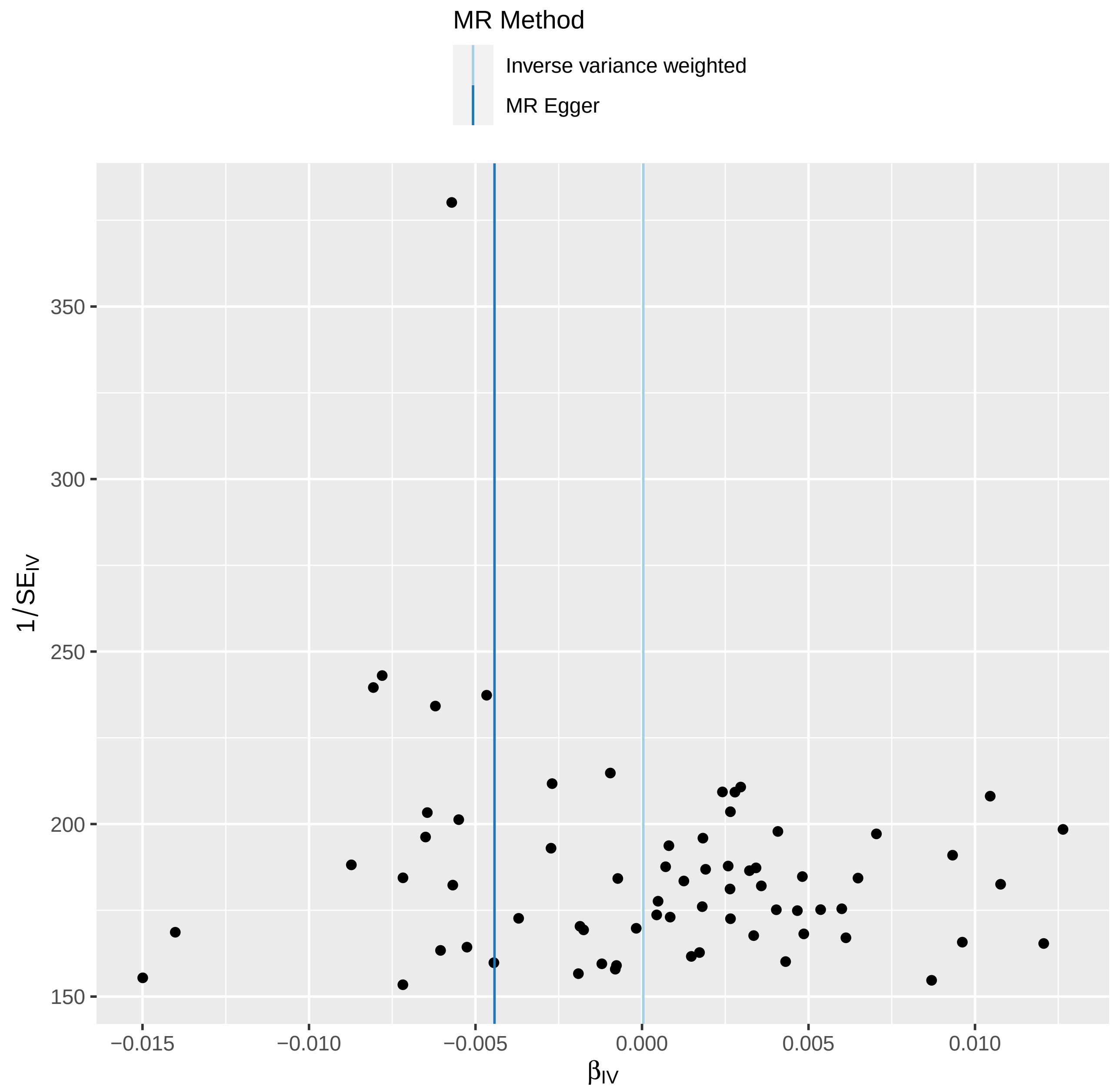

MR Method
Inverse variance weighted
MR Egger
1/SE_IV
350
300
250
200
150
−0.015
−0.010
−0.005
0.000
0.005
0.010
β_IV
