# Supplementary material for: Causal link between mental disorders and gastrointestinal diseases: a Mendelian randomization study
Source: Front Endocrinol (Lausanne). 2025 Apr 22;16:1288619. doi: 10.3389/fendo.2025.1288619 (PMC12052545; doi:10.3389/fendo.2025.1288619)
Supplement: Supplementary file 4 [file DataSheet4.pdf]

Figure S2 Forest plot, scatter plot, leave-one-out analysis and funnel plot for MDs on gastric cancer.

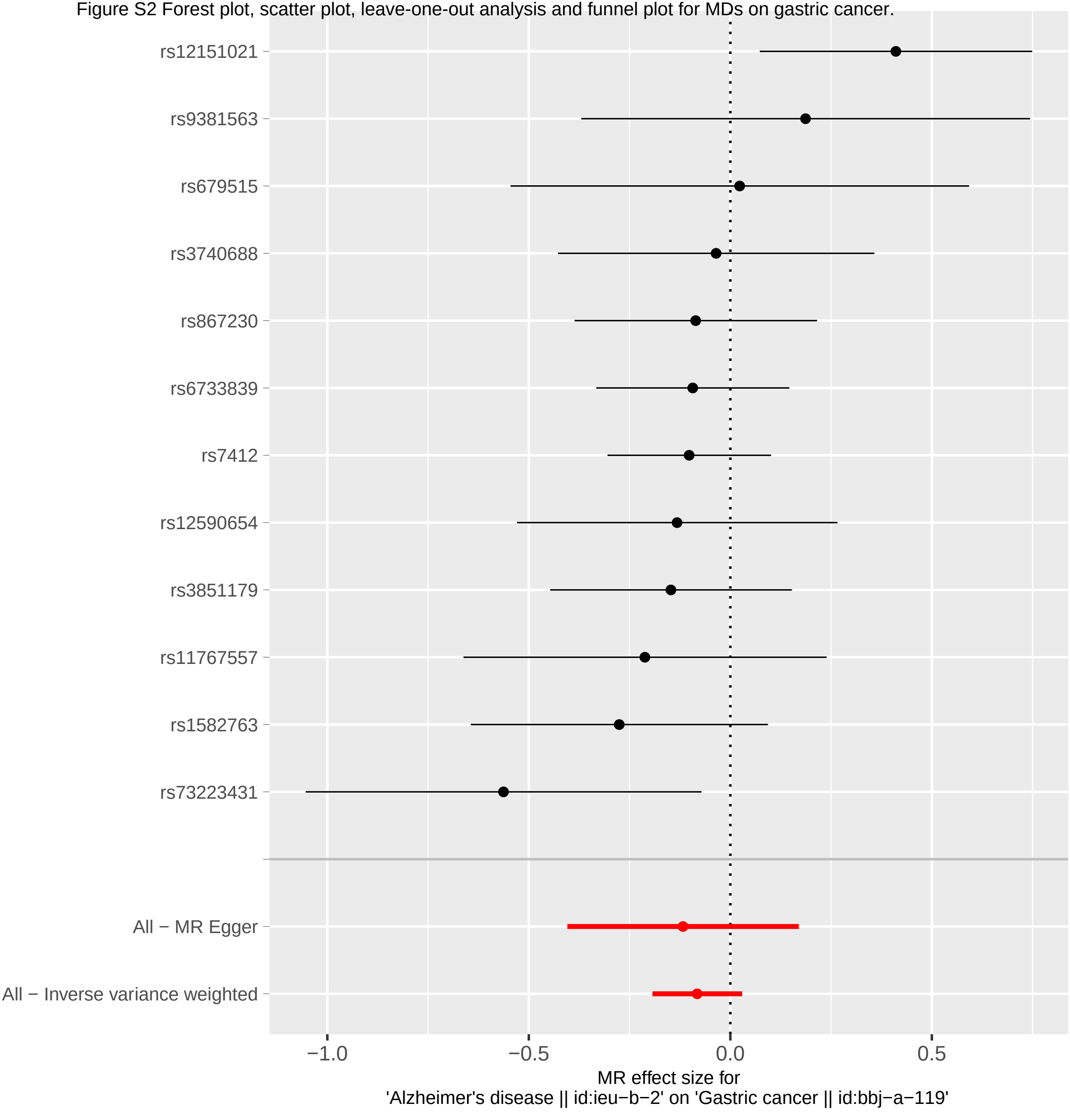

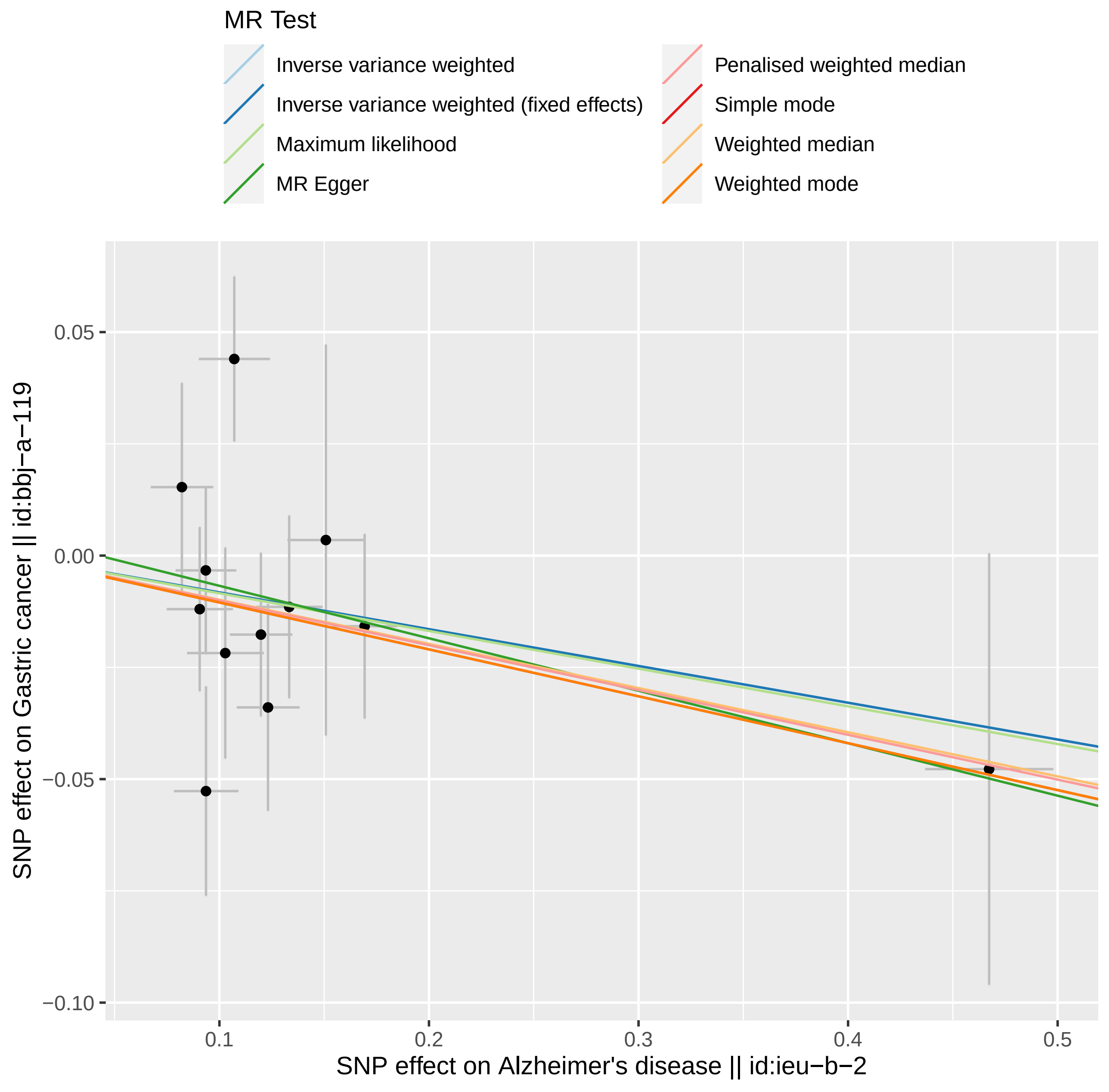

MR Test
Inverse variance weighted
Inverse variance weighted (fixed effects)
Maximum likelihood
MR Egger
Penalised weighted median
Simple mode
Weighted median
Weighted mode
SNP effect on Gastric cancer || id:bbj−a−119
0.05
0.00
−0.05
−0.10
0.1
0.2
0.3
0.4
0.5
SNP effect on Alzheimer's disease || id:ieu−b−2

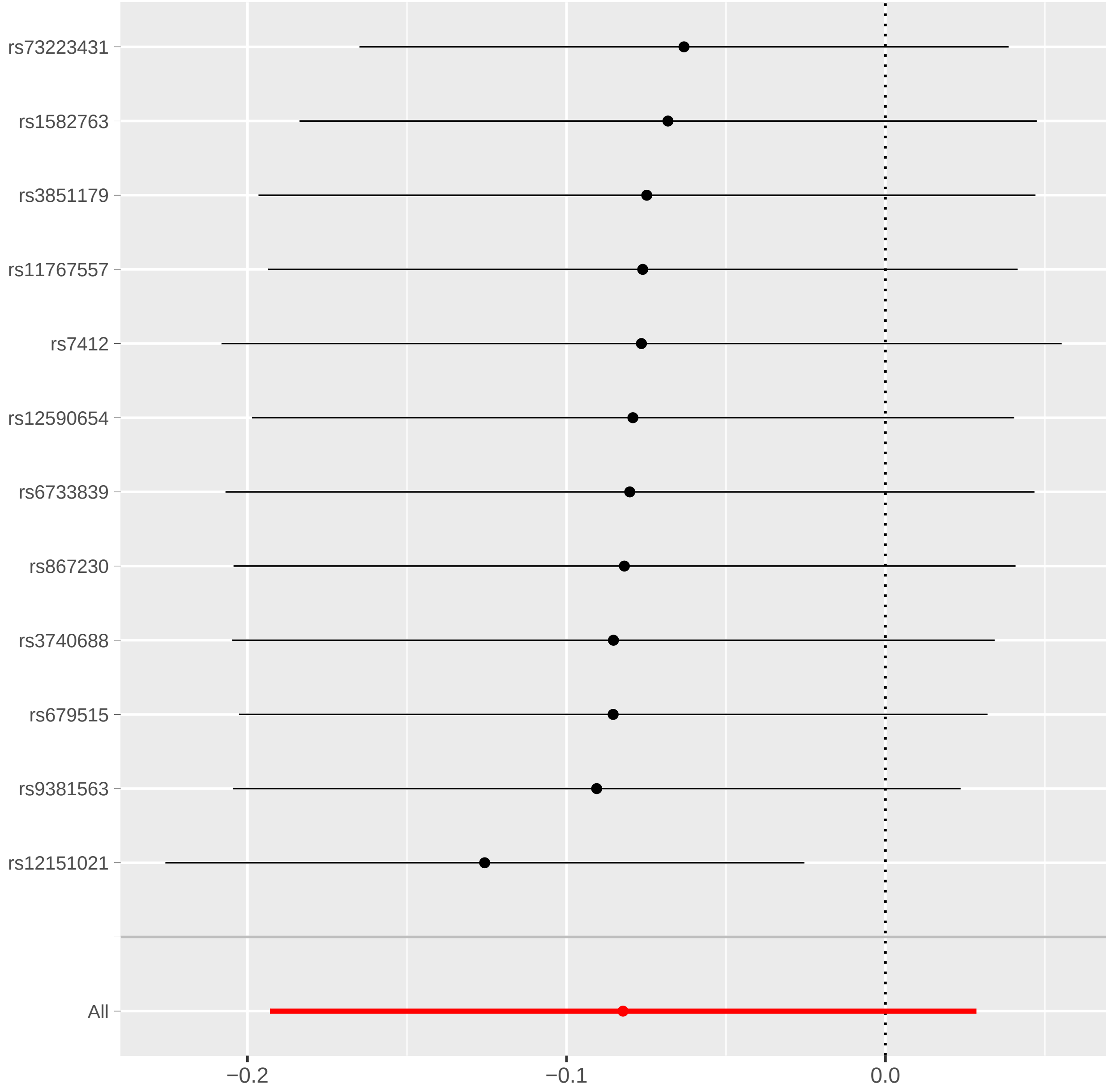

MR leave−one−out sensitivity analysis for
'Alzheimer's disease || id:ieu−b−2' on 'Gastric cancer || id:bbj−a−119'

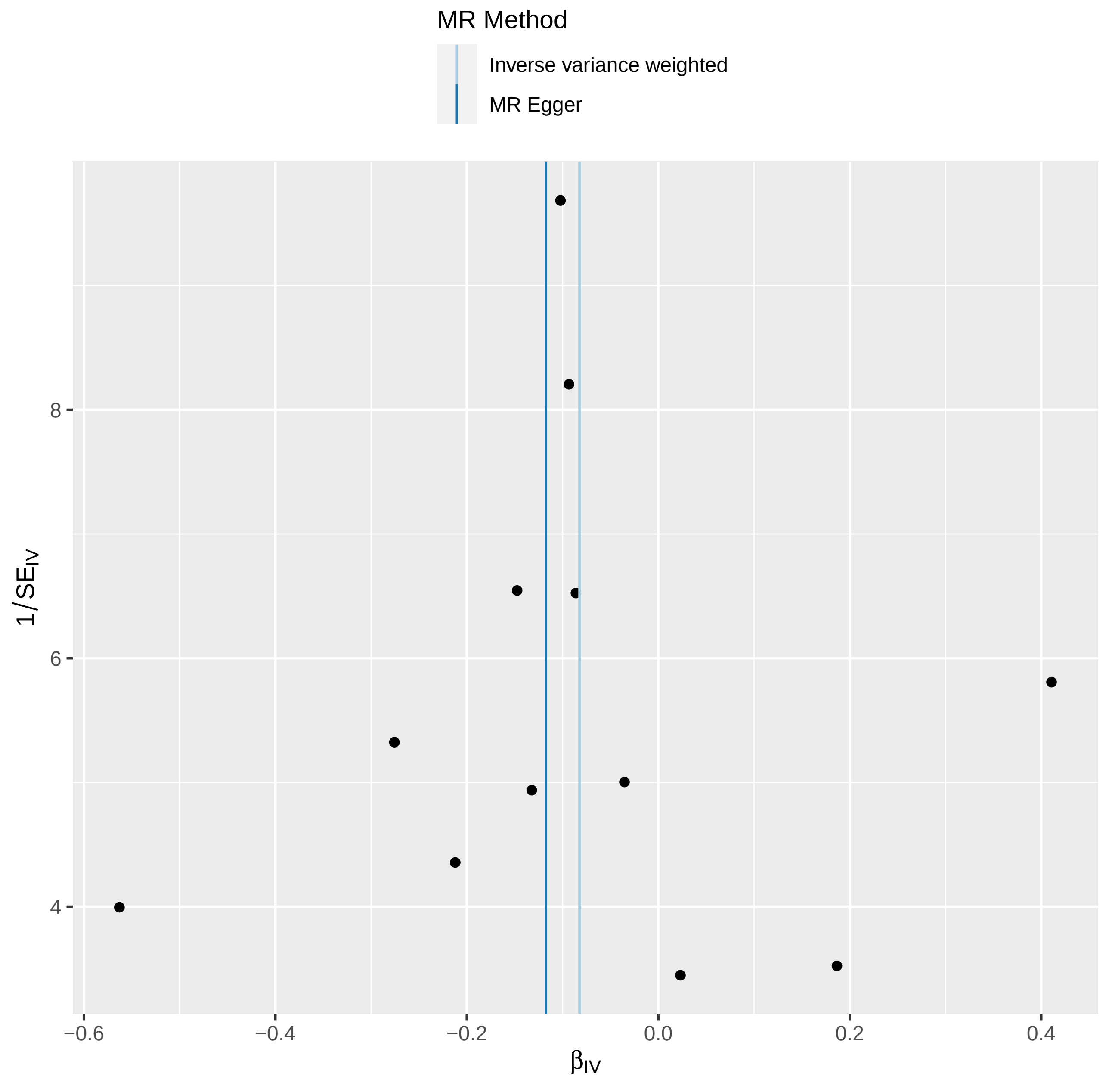

MR Method
Inverse variance weighted
MR Egger
8
6
4
1/SE_IV
−0.6
−0.4
−0.2
0.0
0.2
0.4
β_IV

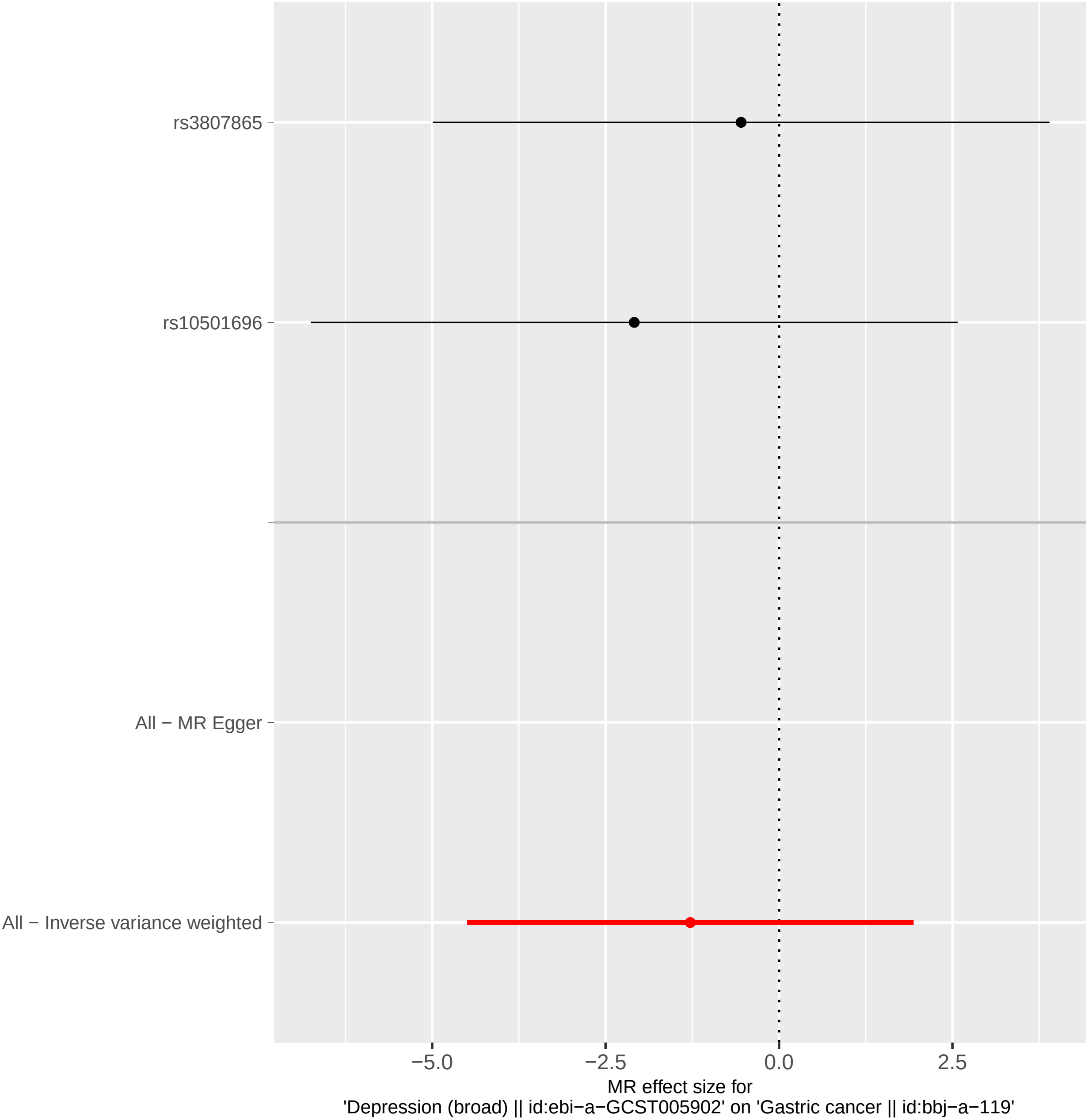
rs3807865
rs10501696
All − MR Egger
All − Inverse variance weighted
−5.0
−2.5
0.0
2.5
MR effect size for
'Depression (broad) || id:ebi−a−GCST005902' on 'Gastric cancer || id:bbj−a−119'

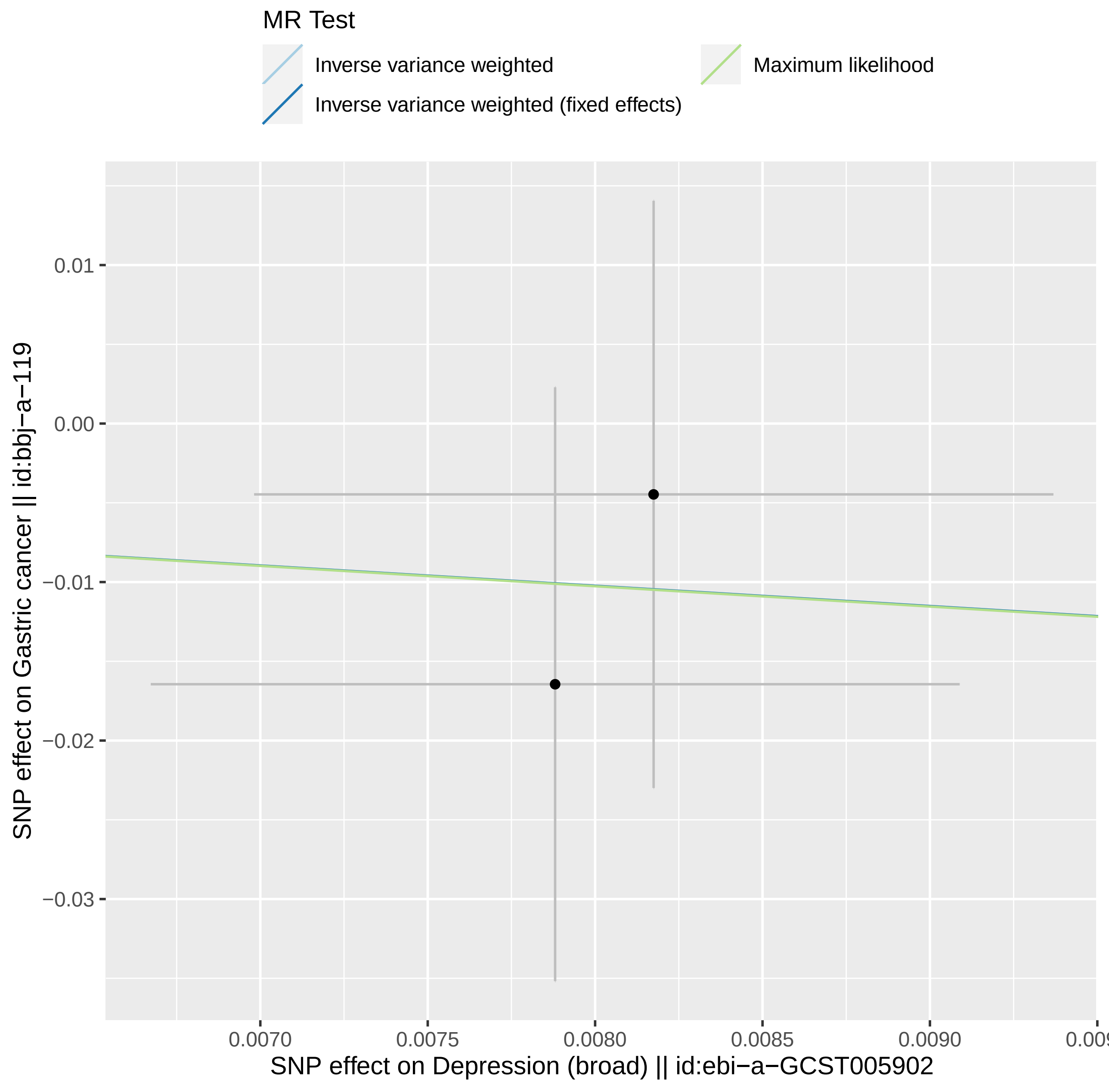

MR Test
Inverse variance weighted
Inverse variance weighted (fixed effects)
Maximum likelihood
0.01
0.00
−0.01
−0.02
−0.03
SNP effect on Gastric cancer || id:bbj−a−119
0.0070
0.0075
0.0080
0.0085
0.0090
SNP effect on Depression (broad) || id:ebi−a−GCST005902

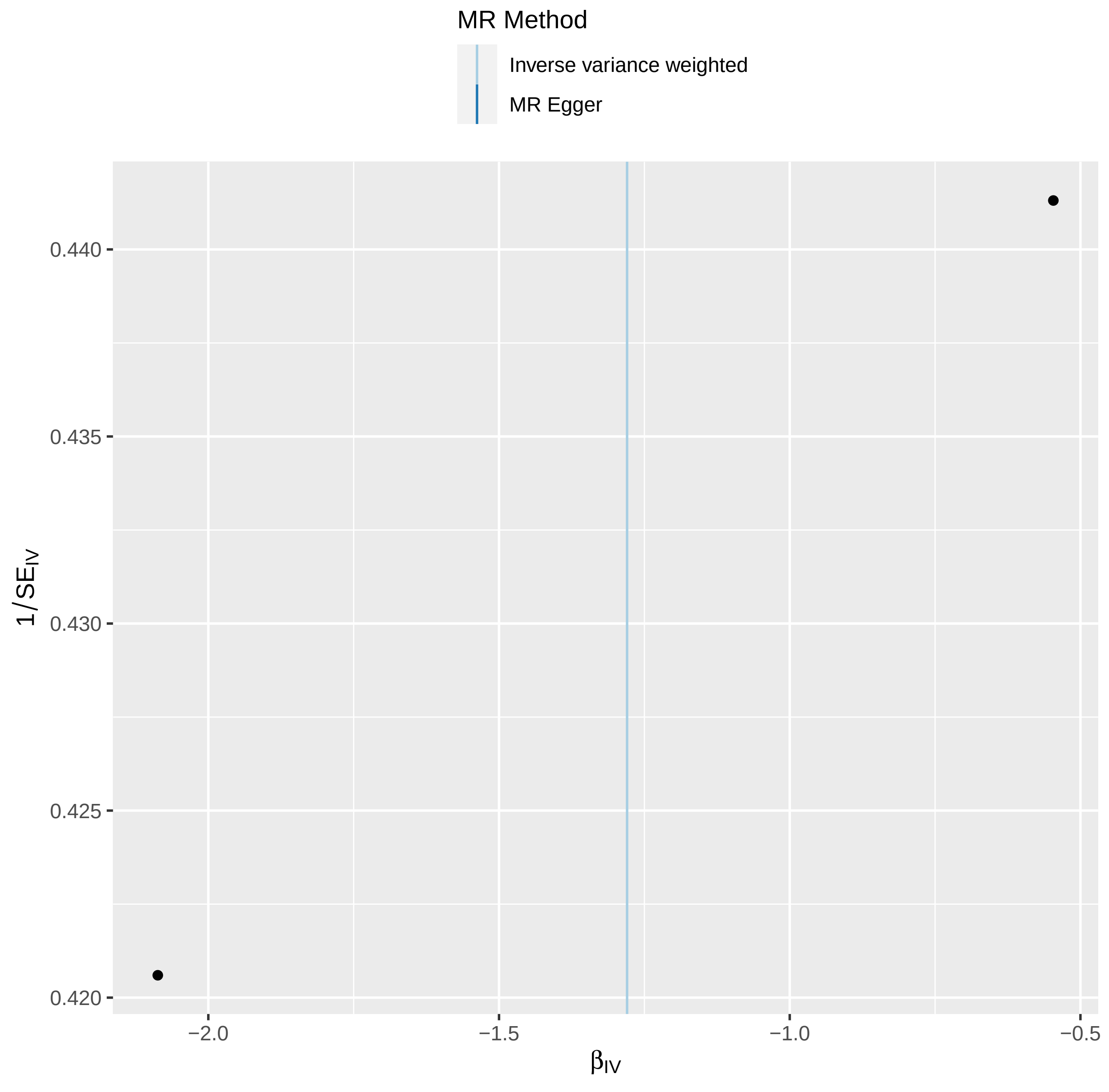
MR Method
Inverse variance weighted
MR Egger
0.440
0.435
0.430
0.425
0.420
1/SE_IV
−2.0
−1.5
−1.0
−0.5
β_IV

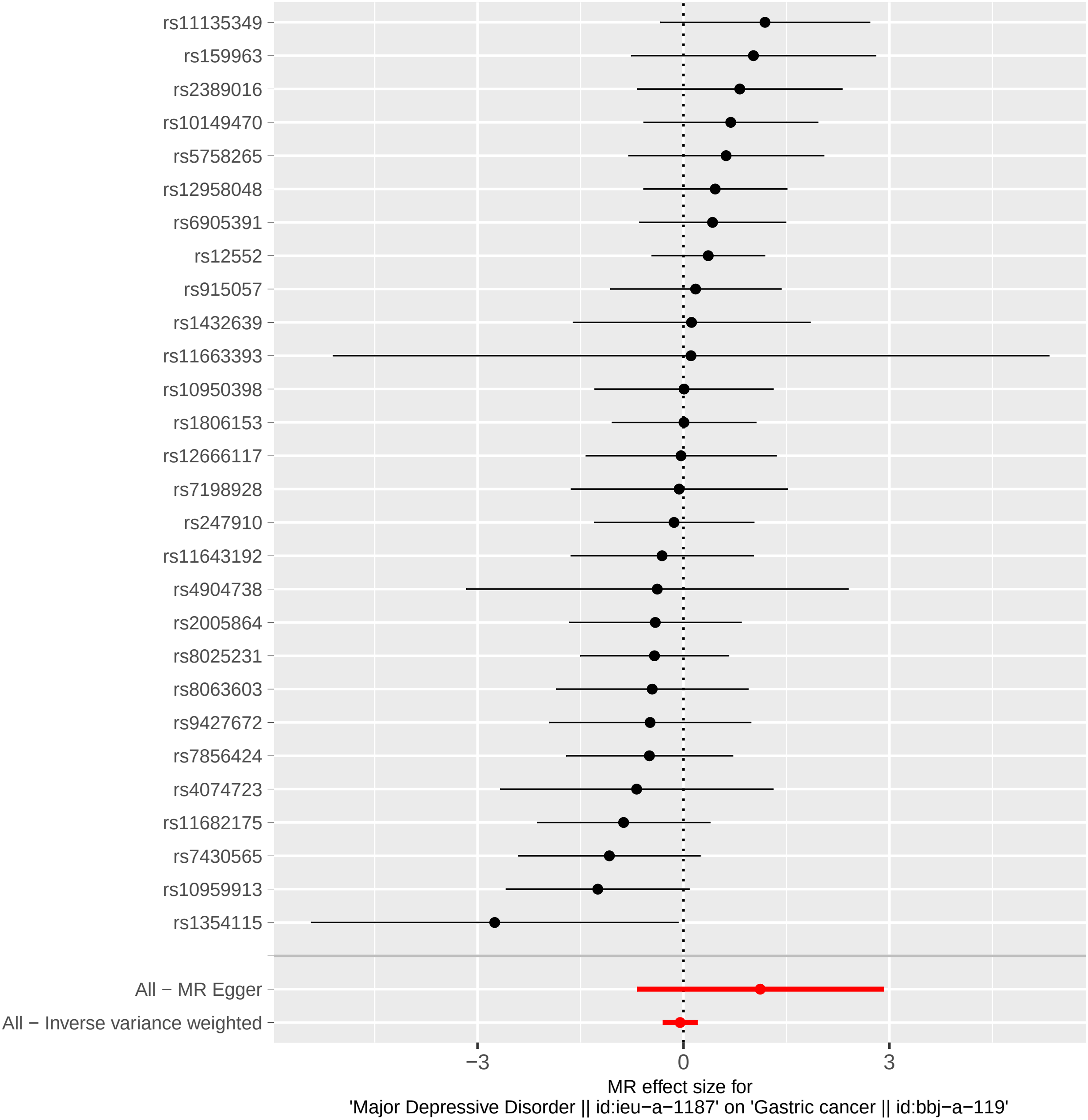
rs11135349
rs159963
rs2389016
rs10149470
rs5758265
rs12958048
rs6905391
rs12552
rs915057
rs1432639
rs11663393
rs10950398
rs1806153
rs12666117
rs7198928
rs247910
rs11643192
rs4904738
rs2005864
rs8025231
rs8063603
rs9427672
rs7856424
rs4074723
rs11682175
rs7430565
rs10959913
rs1354115
All − MR Egger
All − Inverse variance weighted
−3
0
3
MR effect size for
'Major Depressive Disorder || id:ieu−a−1187' on 'Gastric cancer || id:bbj−a−119'

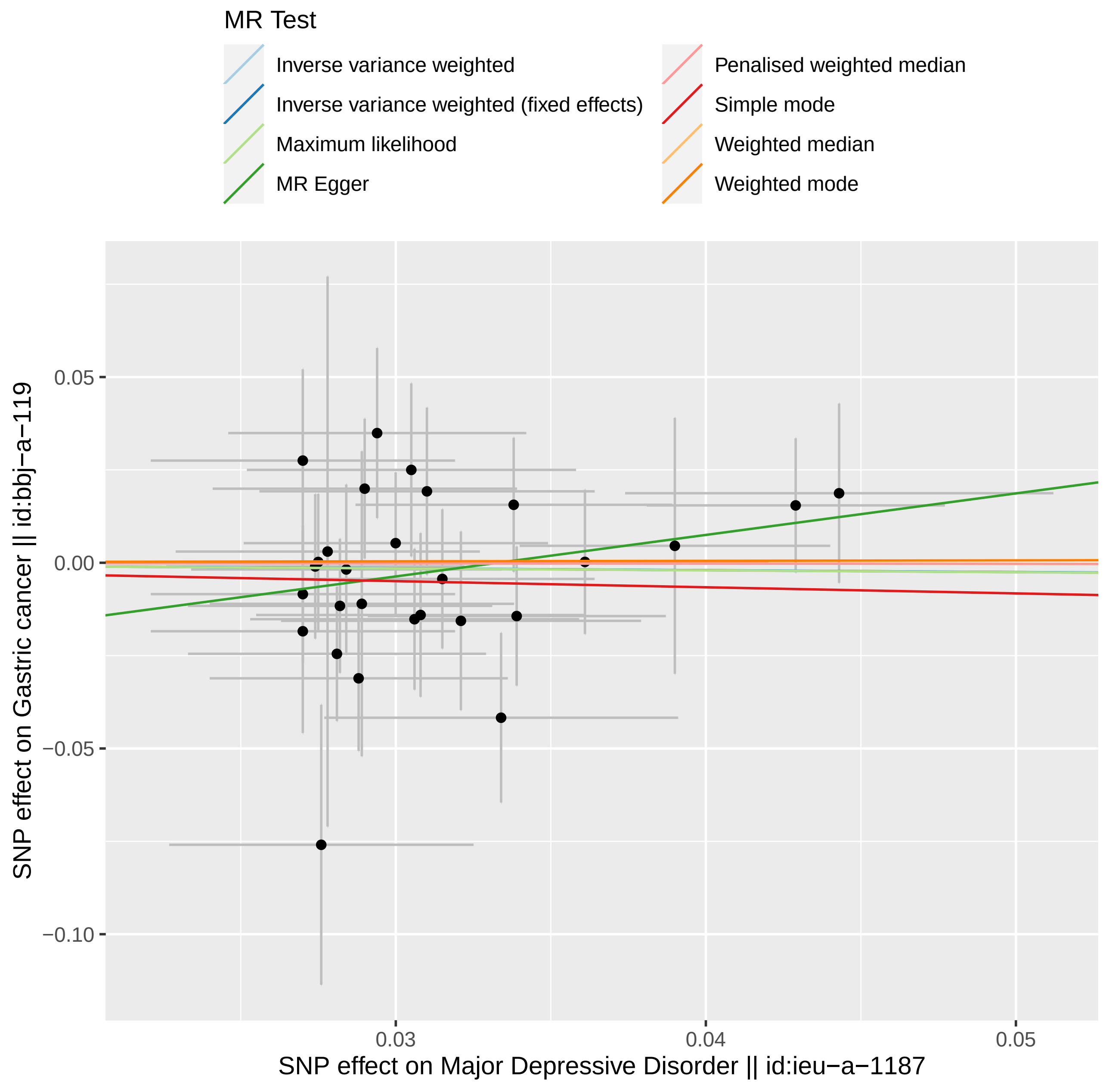

MR Test
Inverse variance weighted
Inverse variance weighted (fixed effects)
Maximum likelihood
MR Egger
Penalised weighted median
Simple mode
Weighted median
Weighted mode
0.05
0.00
−0.05
−0.10
0.03
0.04
0.05
SNP effect on Major Depressive Disorder || id:ieu−a−1187
SNP effect on Gastric cancer || id:bbj−a−119

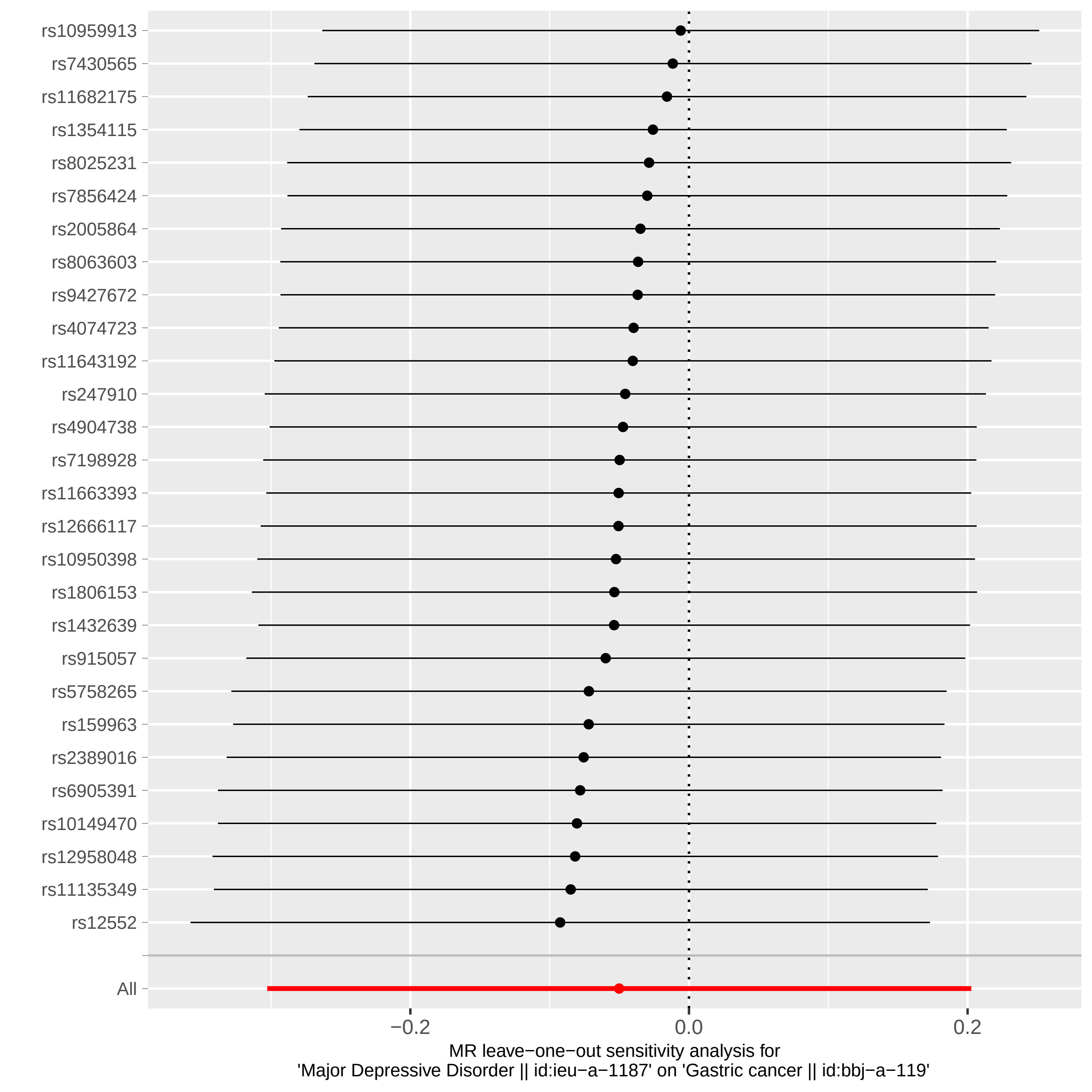
rs10959913
rs7430565
rs11682175
rs1354115
rs8025231
rs7856424
rs2005864
rs8063603
rs9427672
rs4074723
rs11643192
rs247910
rs4904738
rs7198928
rs11663393
rs12666117
rs10950398
rs1806153
rs1432639
rs915057
rs5758265
rs159963
rs2389016
rs6905391
rs10149470
rs12958048
rs11135349
rs12552
All
−0.2
0.0
0.2
MR leave−one−out sensitivity analysis for
'Major Depressive Disorder || id:ieu−a−1187' on 'Gastric cancer || id:bbj−a−119'

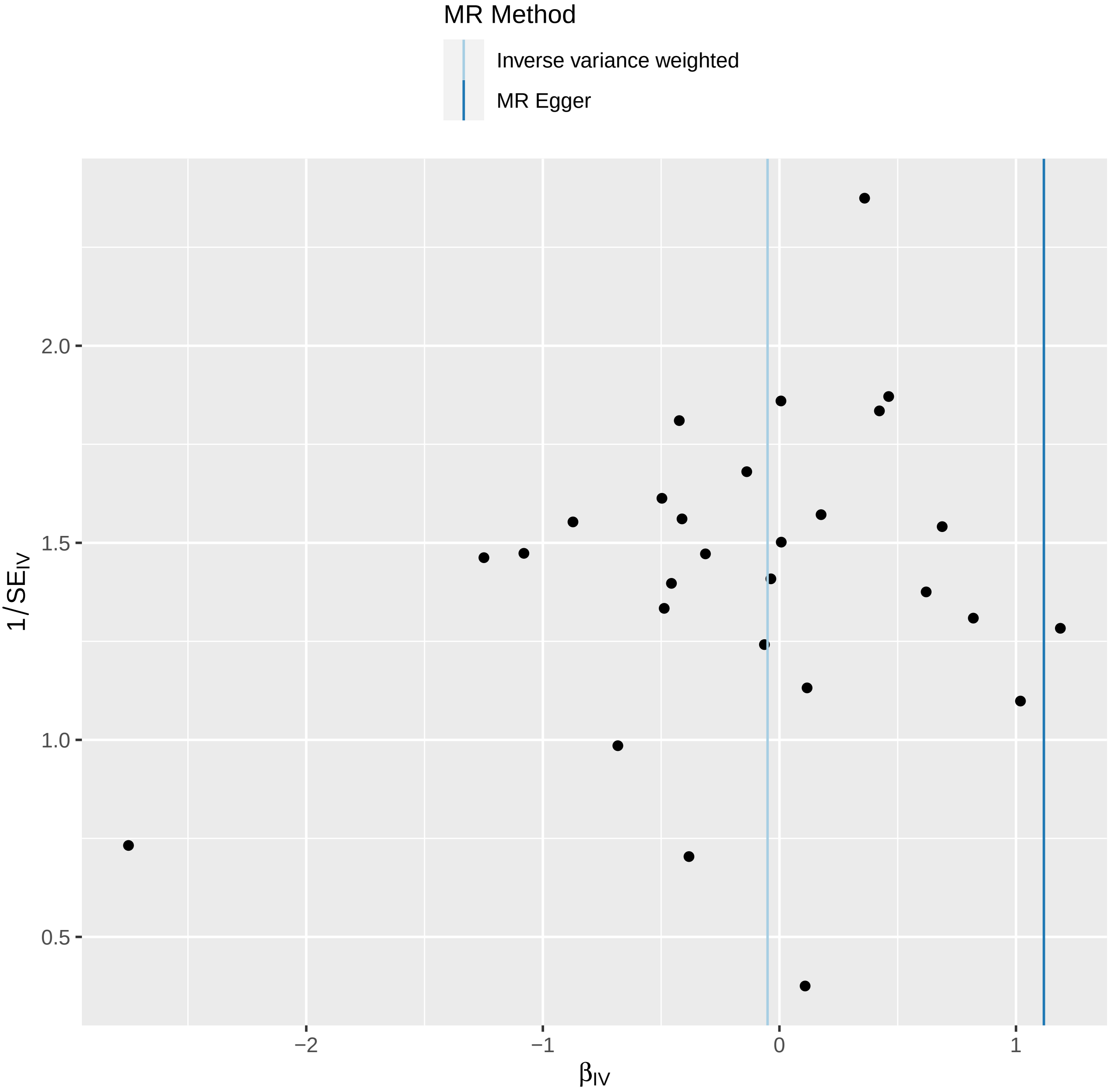

MR Method
Inverse variance weighted
MR Egger
2.0
1.5
1.0
0.5
1/SE$_{IV}$
−2
−1
0
1
β$_{IV}$

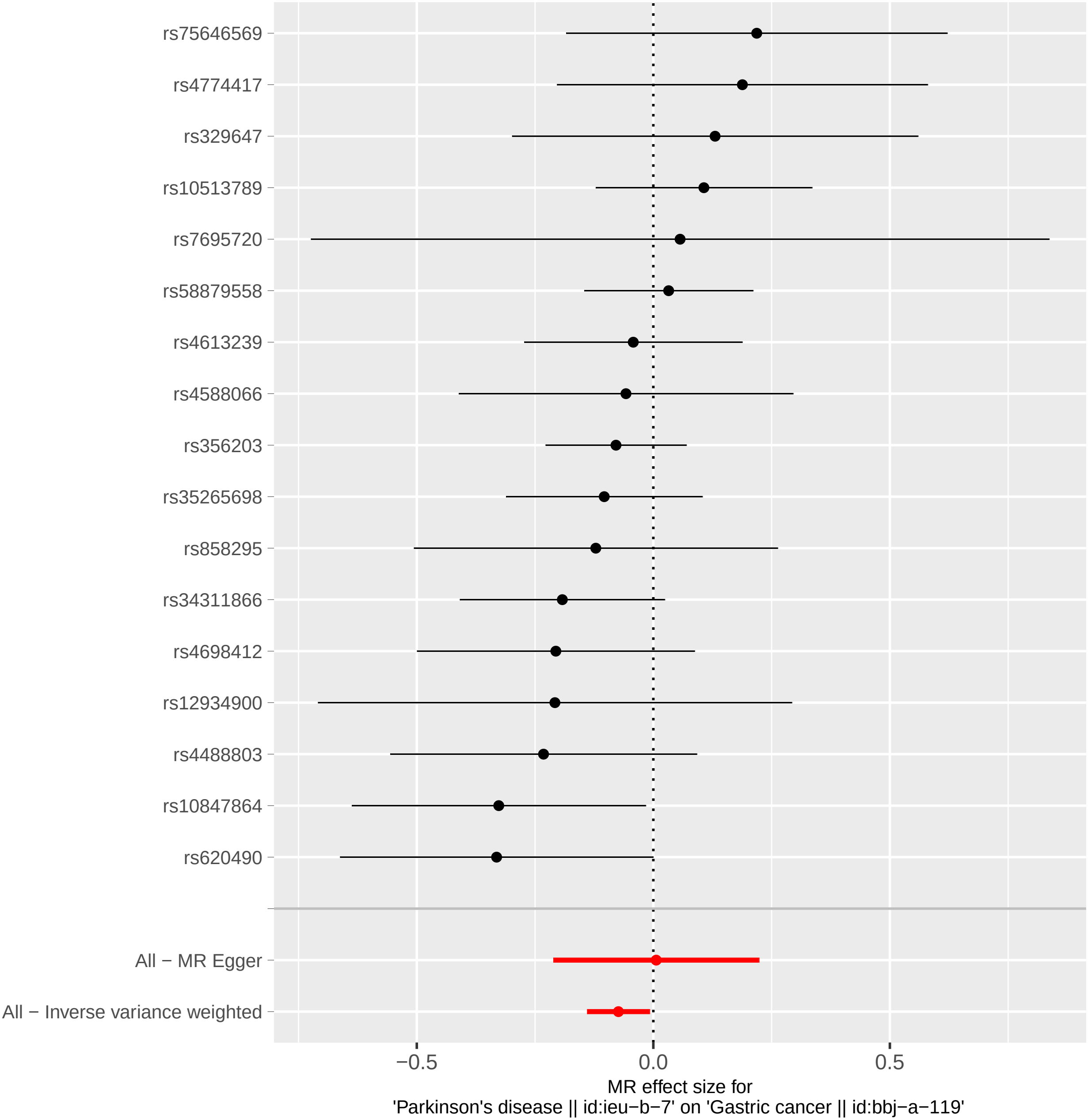
rs75646569
rs4774417
rs329647
rs10513789
rs7695720
rs58879558
rs4613239
rs4588066
rs356203
rs35265698
rs858295
rs34311866
rs4698412
rs12934900
rs4488803
rs10847864
rs620490
All − MR Egger
All − Inverse variance weighted
−0.5
0.0
0.5
MR effect size for
'Parkinson's disease || id:ieu−b−7' on 'Gastric cancer || id:bbj−a−119'

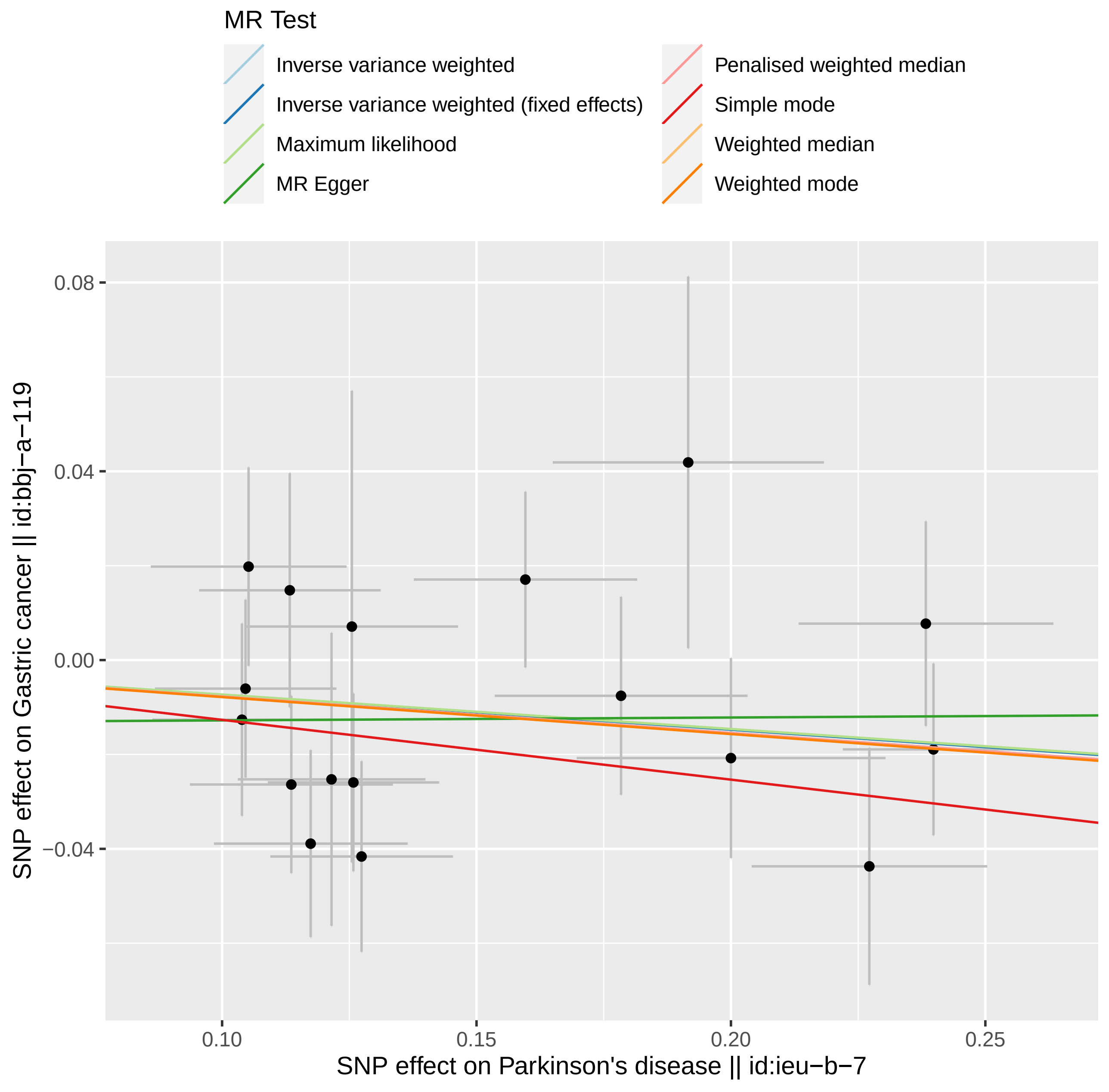

MR Test
Inverse variance weighted
Inverse variance weighted (fixed effects)
Maximum likelihood
MR Egger
Penalised weighted median
Simple mode
Weighted median
Weighted mode
0.08
0.04
0.00
−0.04
0.10
0.15
0.20
0.25
SNP effect on Gastric cancer || id:bbj−a−119
SNP effect on Parkinson's disease || id:ieu−b−7

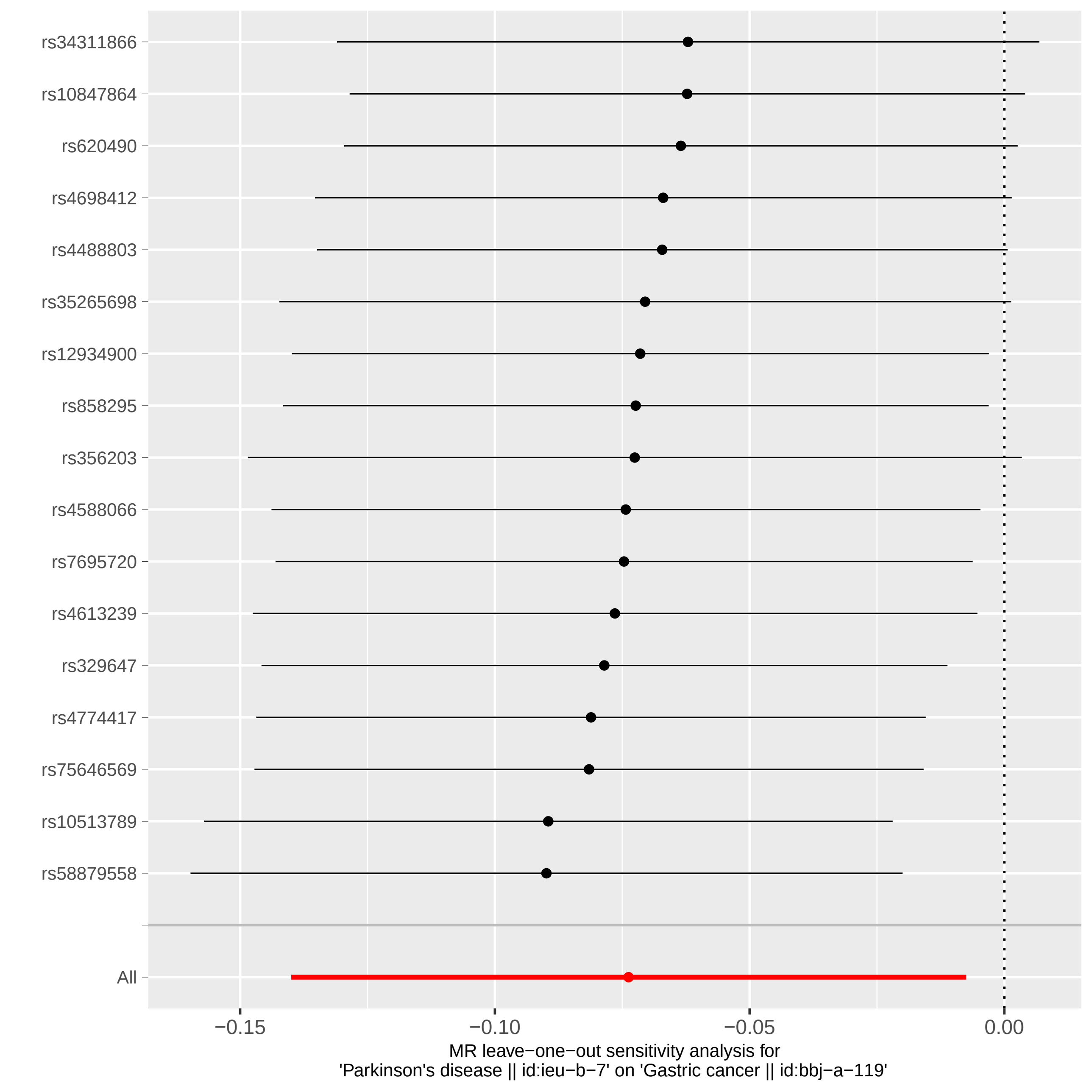
rs34311866
rs10847864
rs620490
rs4698412
rs4488803
rs35265698
rs12934900
rs858295
rs356203
rs4588066
rs7695720
rs4613239
rs329647
rs4774417
rs75646569
rs10513789
rs58879558
All
−0.15
−0.10
−0.05
0.00
MR leave−one−out sensitivity analysis for
'Parkinson's disease || id:ieu−b−7' on 'Gastric cancer || id:bbj−a−119'

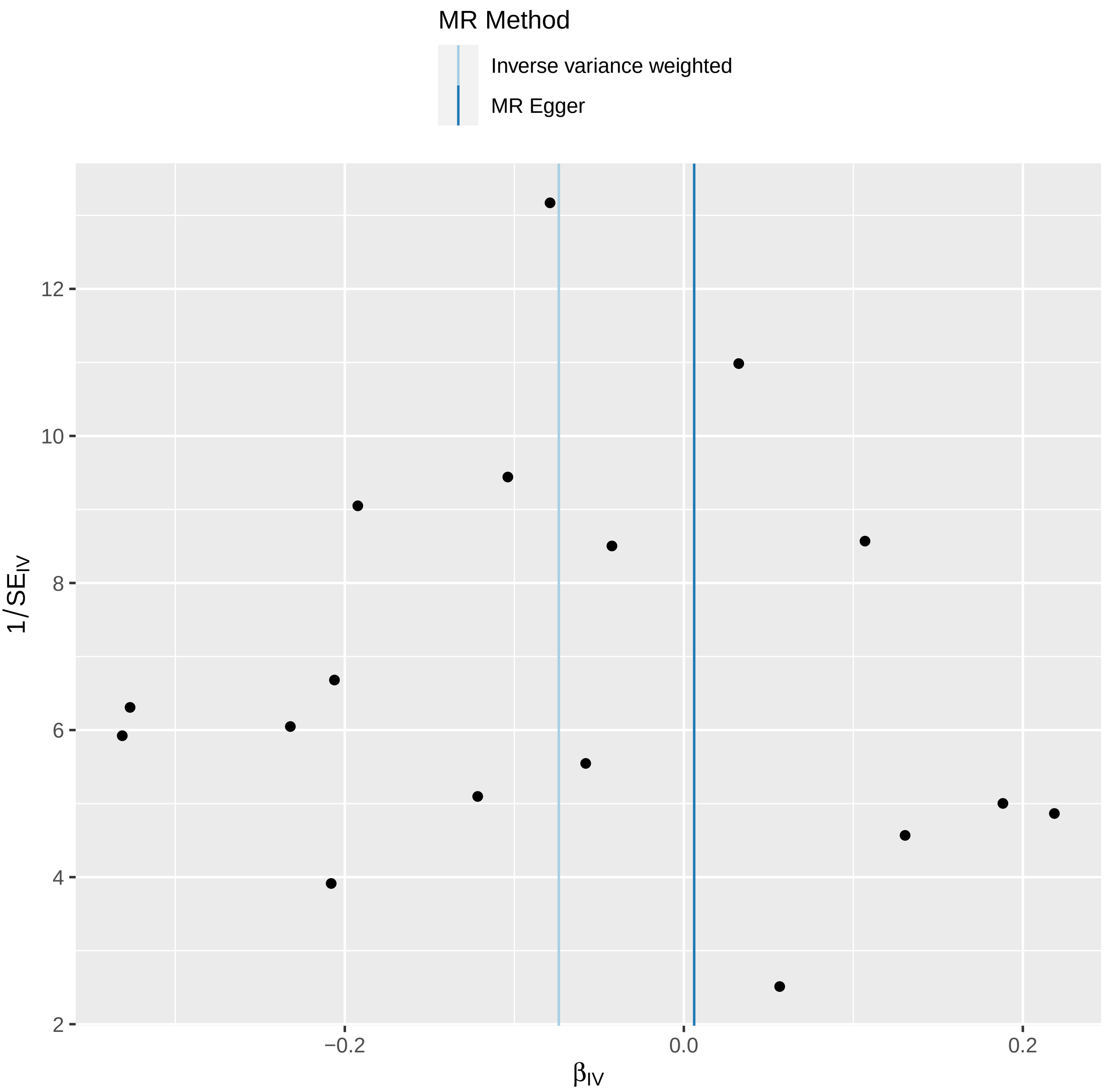

MR Method
Inverse variance weighted
MR Egger
1/SE_IV
12
10
8
6
4
2
−0.2
0.0
0.2
β_IV

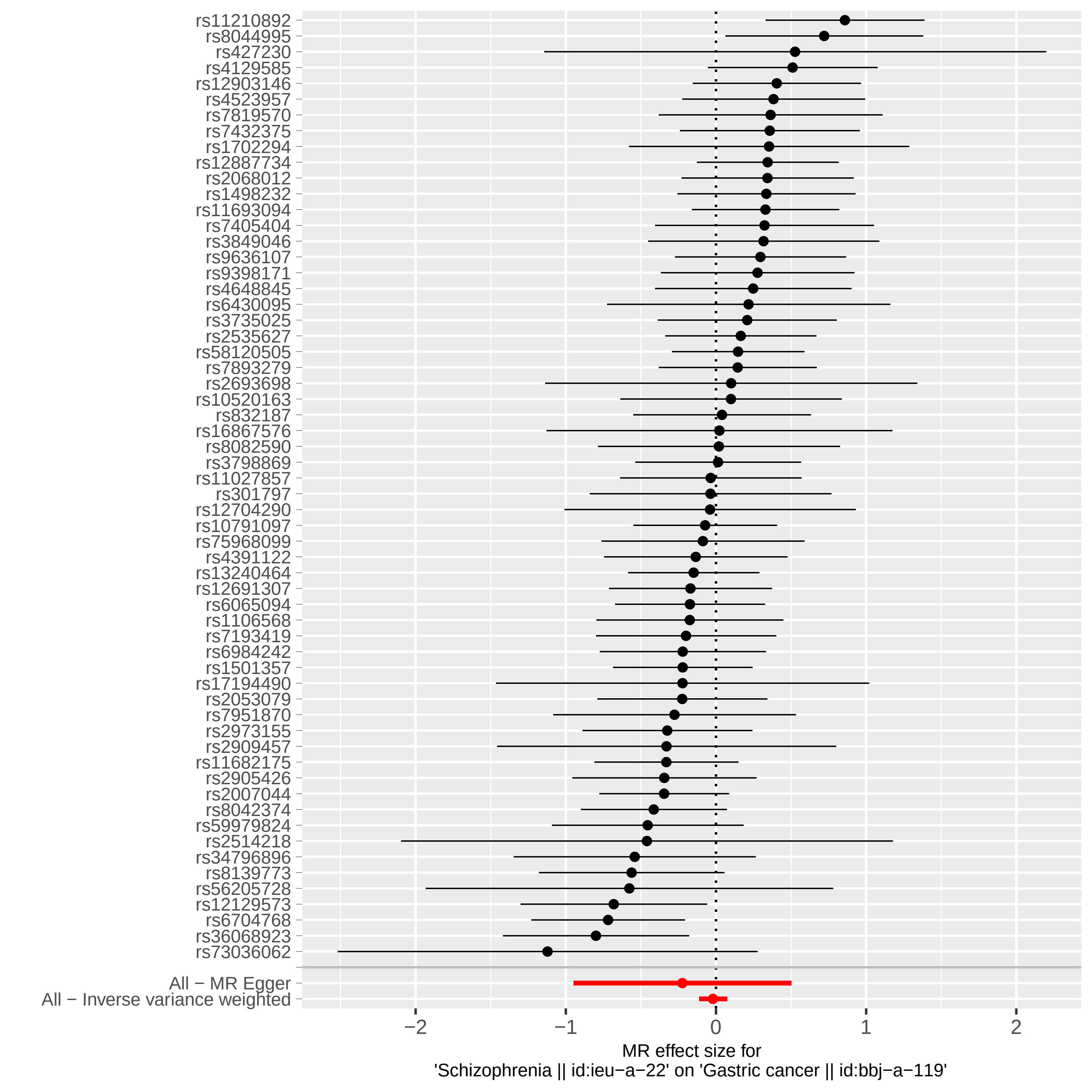

rs11210892
rs8044995
rs427230
rs4129585
rs12903146
rs4523957
rs7819570
rs7432375
rs1702294
rs12887734
rs2068012
rs1498232
rs11693094
rs7405404
rs3849046
rs9636107
rs9398171
rs4648845
rs6430095
rs3735025
rs2535627
rs58120505
rs7893279
rs2693698
rs10520163
rs832187
rs16867576
rs8082590
rs3798869
rs11027857
rs301797
rs12704290
rs10791097
rs75968099
rs4391122
rs13240464
rs12691307
rs6065094
rs1106568
rs7193419
rs6984242
rs1501357
rs17194490
rs2053079
rs7951870
rs2973155
rs2909457
rs11682175
rs2905426
rs2007044
rs8042374
rs59979824
rs2514218
rs34796896
rs8139773
rs56205728
rs12129573
rs6704768
rs36068923
rs73036062
All − MR Egger
All − Inverse variance weighted
−2
−1
0
1
2
MR effect size for
'Schizophrenia || id:ieu−a−22' on 'Gastric cancer || id:bbj−a−119'

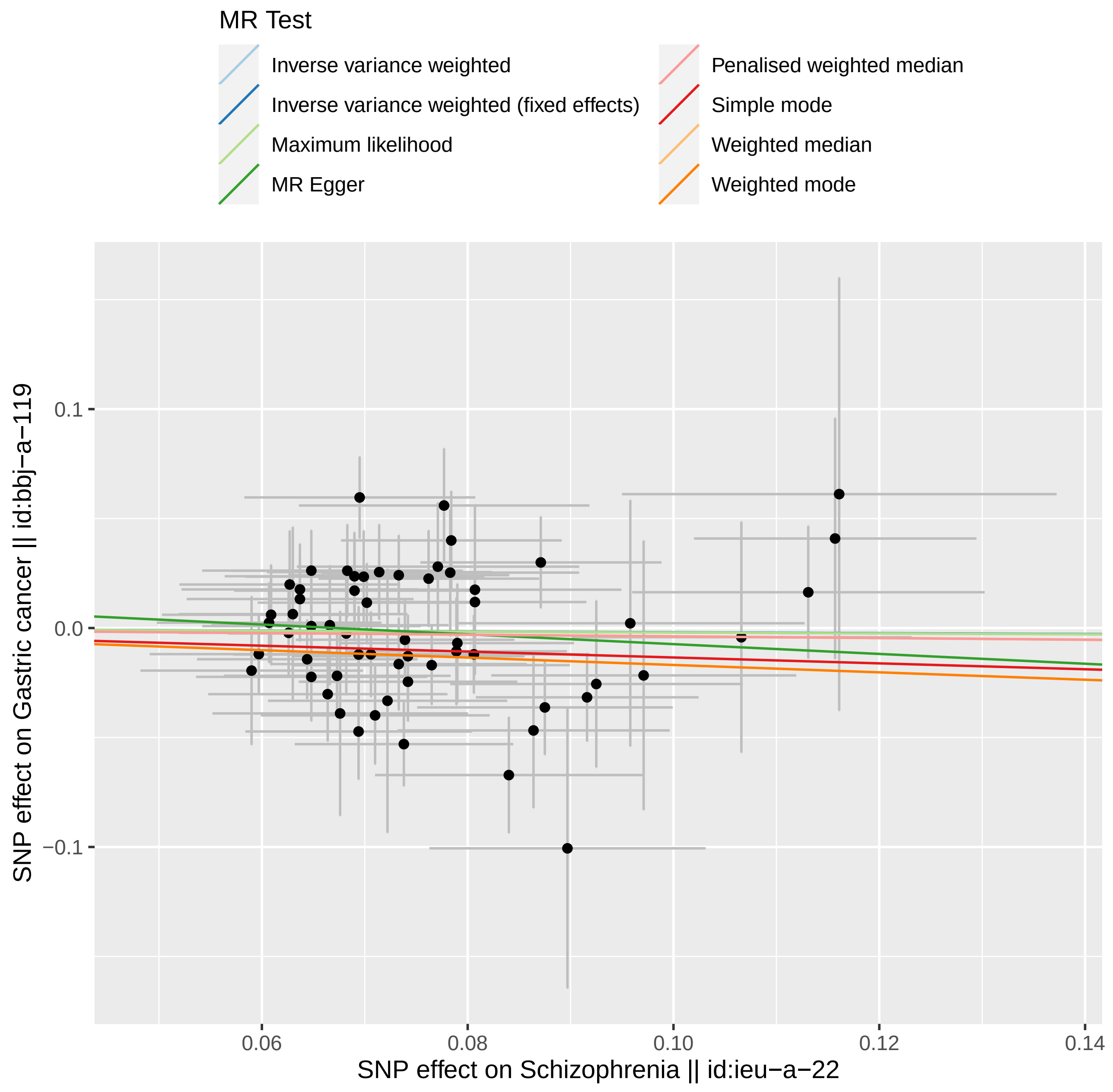
MR Test
Inverse variance weighted
Inverse variance weighted (fixed effects)
Maximum likelihood
MR Egger
Penalised weighted median
Simple mode
Weighted median
Weighted mode
0.1
0.0
−0.1
0.06
0.08
0.10
0.12
0.14
SNP effect on Gastric cancer || id:bbj−a−119
SNP effect on Schizophrenia || id:ieu−a−22

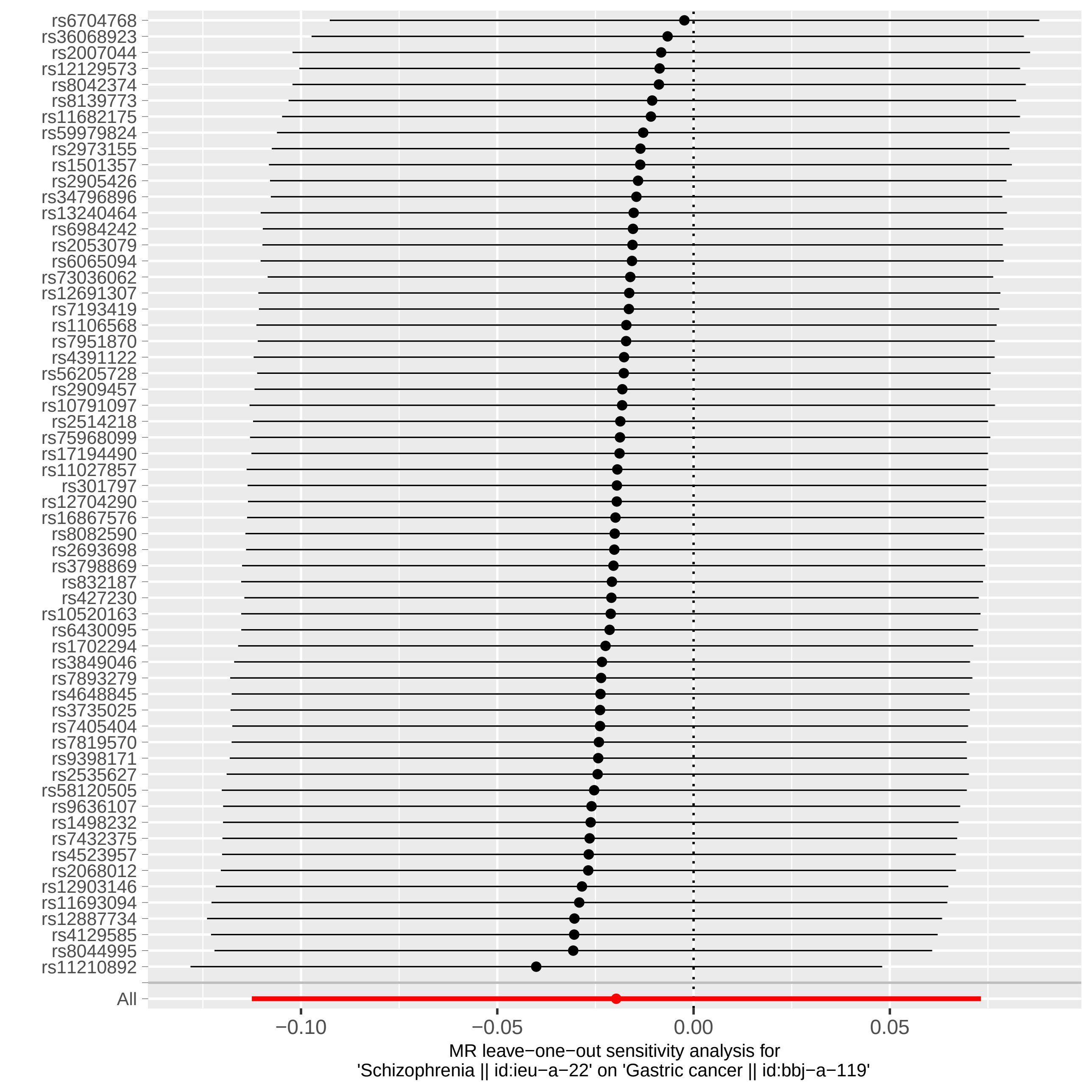
rs6704768
rs36068923
rs2007044
rs12129573
rs8042374
rs8139773
rs11682175
rs59979824
rs2973155
rs1501357
rs2905426
rs34796896
rs13240464
rs6984242
rs2053079
rs6065094
rs73036062
rs12691307
rs7193419
rs1106568
rs7951870
rs4391122
rs56205728
rs2909457
rs10791097
rs2514218
rs75968099
rs17194490
rs11027857
rs301797
rs12704290
rs16867576
rs8082590
rs2693698
rs3798869
rs832187
rs427230
rs10520163
rs6430095
rs1702294
rs3849046
rs7893279
rs4648845
rs3735025
rs7405404
rs7819570
rs9398171
rs2535627
rs58120505
rs9636107
rs1498232
rs7432375
rs4523957
rs2068012
rs12903146
rs11693094
rs12887734
rs4129585
rs8044995
rs11210892
All
−0.10
−0.05
0.00
0.05
MR leave−one−out sensitivity analysis for
'Schizophrenia || id:ieu−a−22' on 'Gastric cancer || id:bbj−a−119'

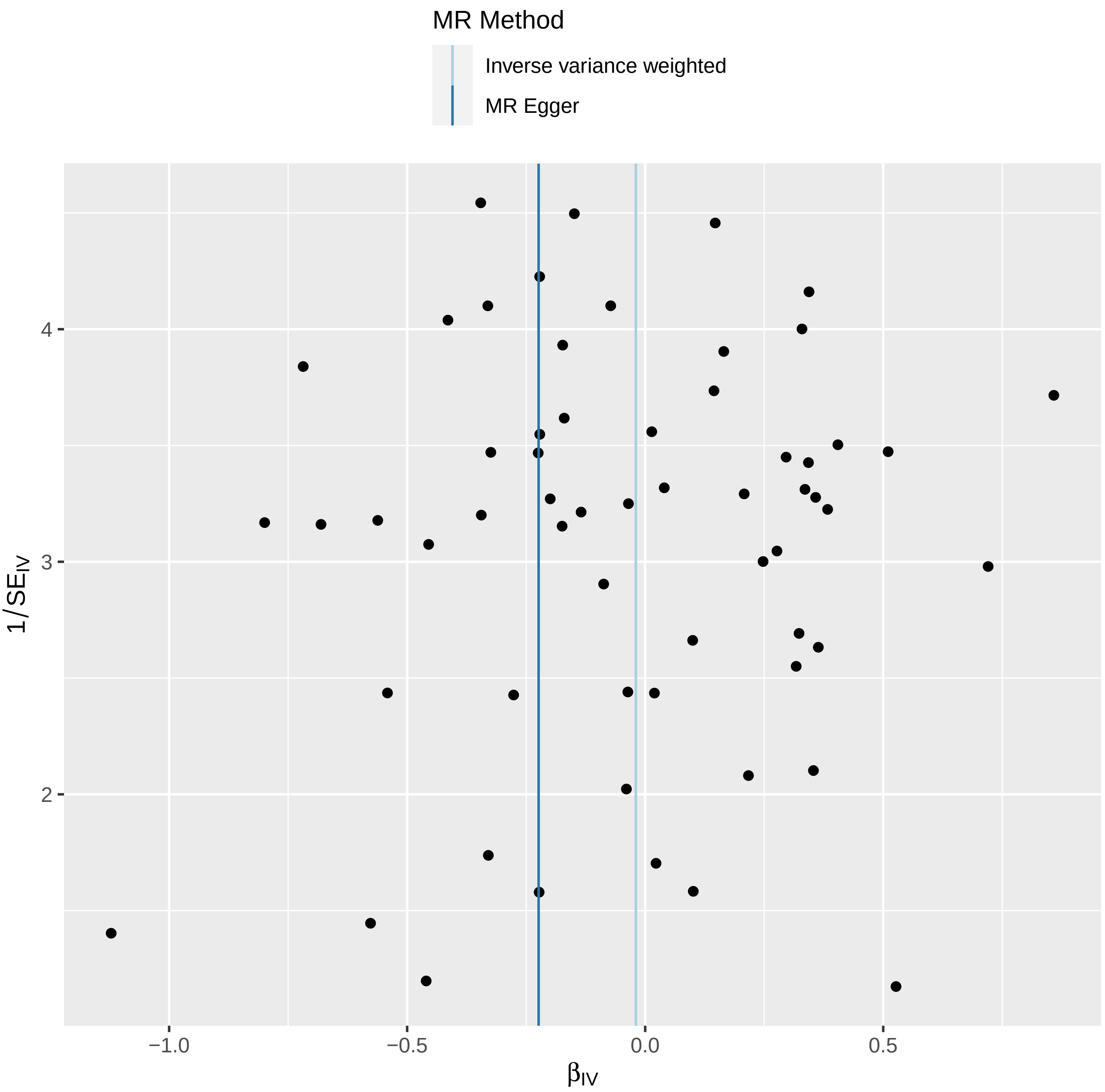

MR Method
Inverse variance weighted
MR Egger
4
3
2
1/SE_IV
−1.0
−0.5
0.0
0.5
β_IV
