# Supplementary material for: Causal link between mental disorders and gastrointestinal diseases: a Mendelian randomization study
Source: Front Endocrinol (Lausanne). 2025 Apr 22;16:1288619. doi: 10.3389/fendo.2025.1288619 (PMC12052545; doi:10.3389/fendo.2025.1288619)
Supplement: Supplementary file 5 [file DataSheet5.docx]

library(TwoSampleMR)

library(MRPRESSO)

#exposure_data

exposure_dat <- extract_instruments(outcomes = 'ieu-a-22',

clump = TRUE,

r2 = 0.001,

kb = 10000,

access_token = NULL)

#gastric_cancer

outcome_dat <- extract_outcome_data(snps = exposure_dat_1$SNP,

outcomes ='bbj-a-119',

proxies = FALSE,

maf_threshold = 0.01,

access_token = NULL)

Mydata <- harmonise_data(exposure_dat = exposure_dat_1,

outcome_dat = outcome_dat,

action = 2)

res <- mr(Mydata, method_list=c("mr_ivw",

"mr_ivw_fe",

"mr_two_sample_ml",

"mr_egger_regression",

"mr_weighted_median",

"mr_penalised_weighted_median",

"mr_simple_mode",

"mr_weighted_mode"))

OR <- generate_odds_ratios(res)

mr_presso(BetaOutcome = "beta.outcome",

BetaExposure ="beta.exposure",

SdOutcome = "se.outcome",

SdExposure = "se.exposure",

OUTLIERtest = TRUE,

DISTORTIONtest = TRUE,

data = Mydata,

NbDistribution = 1000,

SignifThreshold = 0.05,

seed = 1234)

mr_heterogeneity(Mydata,

method_list = c("mr_egger_regression",

"mr_ivw"))

pleio <- mr_pleiotropy_test(Mydata)

#leaveoneout

single <- mr_leaveoneout(Mydata)

mr_leaveoneout_plot(single)

#scatter_plot

mr_scatter_plot(res, Mydata)

#forest_plot

res_single<- mr_singlesnp(Mydata)

mr_forest_plot(res_single)

#funnel_plot

mr_funnel_plot(res_single)
